# Genome-wide analysis of the *NF-Y* gene family in peach (*Prunus persica* L.)

**DOI:** 10.1186/s12864-019-5968-7

**Published:** 2019-07-26

**Authors:** Miao Li, Guixiang Li, Wei Liu, Xiaomin Dong, Anning Zhang

**Affiliations:** Shandong Institute of Pomology, Taian, Shandong China

**Keywords:** Peach, Nuclear Factor Y, Genomics, Drought stress, Transcription factors

## Abstract

**Background:**

Nuclear Factor Y (NF-Y) is a heterotrimeric complex composed of three unique subunits: NF-YA, NF-YB, and NF-YC. The NF-Y transcription factor complex binds to the *CCAAT* box of eukaryotic promoters, playing a vital role in various biological processes in plants. However, the *NF-Y* gene family has not yet been reported from the peach genome. The current study identified and classified candidate peach *NF-Y* genes for further functional analysis of this family.

**Results:**

The current study identified 24 Nuclear Factor Y (NF-Y) transcription factor subunits (6 NF-YA, 12 NF-YB, and 6 NF-YC subunits) in peach. These NF-Y subunits were described with respect to basic physicochemical characteristics, chromosome locations, gene structures, and conserved domains. Based on an analysis of the phylogenetic relationships among peach *NF-Ys*, six pairs of paralogous *NF-Ys* were detected. The expansion of the peach *NF-Y* family occurred by segmental and tandem duplication. Phylogenetic gene synteny of NF-Y proteins was observed between peach and *Arabidopsis*, and five pairs of paralogous NF-Y proteins from peach and *Arabidopsis* were identified. Twenty-four peach *NF-Y*s displayed a diversity of tissue expression patterns. In addition, drought-responsive *cis*-elements were observed in peach *NF-Y* promoters, and 9 peach *NF-Y* genes were shown to distinctly increase their transcript abundances under drought stress.

**Conclusions:**

This study identified 24 *NF-Y* genes in the peach genome and analysed their properties at different levels, providing a foundation for researchers to understand this gene family in peach. The up-regulation of 9 *NF-Y* genes under drought stress indicates that they can serve as candidate functional genes to further study drought resistance in peach.

**Electronic supplementary material:**

The online version of this article (10.1186/s12864-019-5968-7) contains supplementary material, which is available to authorized users.

## Background

Transcription factors (TFs) play an important role in regulating multiple physiological processes in all living organisms. In general, TFs can be divided into different families by their recognizable conserved domains, such as MYB [17], GRAS [38], NAC [51, 52] and BZIP [44, 45]. Similar protein sequences generally indicate that TF family members have similar biological functions, which facilitates the identification and analysis of unknown proteins in other species. For example, *AtNF-YB1* can enhance resistance in *Arabidopsis* under drought conditions and an orthologous maize *NF-Y* factor, *ZmNF-YB2*, was shown to have an equivalent activity in maize [28]. NUCLEAR FACTOR Y (NF-Y), also called heme-activated protein (HAP) or *CCAAT* binding factor (CBF), is a heterotrimeric complex composed of three unique subunits: NF-YA, NF-YB, and NF-YC [25]. In the promoter regions, the NF-Y TF binds to *cis*-elements with the conserved core sequence *CCAAT* to activate or inhibit the expression of related functional genes in metabolism [25]. Three subfamilies (NF-YA, NF-YB, and NF-YC) can be characterized by their conserved domains and sequence lengths. In general, NF-YA sequences are longer than those of NF-YB and NF-YC. NF-YA has a core region composed of 53 amino acids, which contains two conserved domains, A1 and A2 [27]. NF-YB sequences are shorter than those of NF-YC. The protein structures of NF-YB and NF-YC are similar to those of H2B and H2A histones, respectively [27]. Initially, NF-YB and NF-YC form a dimer in the cytoplasm. This dimer is transferred into the nucleus, where it interacts with NF-YA to complete the assembly of the heterotrimeric complex [39].

In recent years, the role of members of the *NF-Y* gene family under drought stress has been a popular research topic. In several species, *NF-Y* gene family members have been identified across their genomes and analysed under drought stress. In *Citrus*, a total of 22 *CsNF-Y* genes have been identified and the candidate gene *CsNF-YA5* was shown to exerted distinct effects on the dehydration tolerance of transgenic tobacco [33]. In castor bean, 25 *RcNF-Y* genes have been identified across the genome and their expression changes were investigated under four types of abiotic stresses (drought, cold, heat and salt stresses) [44, 45]. In chickpea, a total of 40 *CaNF-Y* genes have been identified and some *CaNF-Y* genes were found to be responsive to dehydration and abscisic acid treatments [9].

Peach (*Prunus persica* L.) is one of the most popular fruits worldwide. Because of its small genome size, economic and nutritional importance, and short reproductive cycle [7], peach has become a model tree species for plant physiology and genetics research. With the release of the whole genome sequence of peach [15], it has become convenient to analyse entire gene families. The *NF-Y* gene family plays vital roles in various physiological processes and regulatory networks. However, a detailed analysis of the peach *NF-Y* (designated *PpNF-Y*) gene family has not yet been performed. Considering all these points, the aim of this study was to understand the members of the *NF-Y* gene family in peach and explore the roles of these genes under drought stress. In this study, based on the sequence information derived from public databases, *PpNF-Y* members were identified and classified. Gene duplication was analysed to identify the evolutionary origins of *PpNF-Y* gene family. In addition, the current study analysed the phylogenetic relationships of NF-Y proteins in peach and *Arabidopsis* to further elucidate the biological functions of *PpNF-Y* genes. Tissue expression profiles were examined to further investigate the roles of *PpNF-Y* genes during the development of different organs. Moreover, expression analysis during drought stress treatment indicated that some *PpNF-Y* genes responded to drought stress and these genes served as candidate genes for drought resistance in peach.

## Methods

### Plant materials and drought treatments

*Prunus davidiana* seeds (Pd) were collected from the Jinniu Mountain Scientific Research base of Shandong Institute of Pomology, Shandong, China, located at 35°38′N and 116°20′E. The seeds were grown in plastic pots (upper diameter 7.0cm, lower diameter 5.0cm, height 7.8cm) filled with mixed soil (vermiculite:humus = 1:1) (without chemical fertilizer application) and were well watered for 30d under greenhouse conditions with 24/18°C day/night, 16/8 h light/dark and 70% relative humidity [32]. In the experiment, there were two groups, a control group and a treated group. Each of them contained 10 peach seedlings. The drought stress treatments were carried out on ten Pd seedlings with similar stem lengths and leaf areas for ten days by withholding water until distinct wrinkling was observed in the top three leaves [29]. The control pot contained another ten similar Pd seedlings that were slightly watered every three days to keep the soil moist. Leaf samples from the treated and control groups were collected, immediately frozen in liquid nitrogen and stored at -70°C [1]. In the tissue expression pattern experiment, samples of five tissues including roots, stems, leaves, flowers and fruits were collected from the cultivar Sunagowase, immediately frozen in liquid nitrogen and stored at -70°C [1].

### Identification and analysis of *PpNF-Y*s

Siefers et al. [37] described the conserved regions of the three NF-Y subfamilies: the NF-YA conserved region contains the amino acid sequence f-V-N-A-K-Q-Y-h-x-I-l-r-R-R-q-x-R-A-k-l-E-a-x-x-K-l-i-k-x- R-K-P-Y-l-H-E-S-R-H-x-H-A-x-r-R-p-R-G-s-G-G-R-E, the NF-YB conserved region contains the amino acid sequence r-e-q-D-r-x-L-P-I-A- N-v-x-R-I-M-K-x-x-L-P-x-x-n-x-k-i-s-k-D-A-K-e-t-x-Q-E-C-v-s-E-F-I-S-F-v-T-s-E-A-s-d-k-C-q-x-E-k-R-K-T-I-n-g-d-D-x-L-w-A-m-x-t-L-G-F-x-d-Y-x-e-p-L-x-k-x-Y-x-L-x-k-y-R-e-x-x-e-g-e, and the NF-YC conserved region contains the amino acid sequence l-P-l-a-R-I-K-K-I-M- K-x-D-e-D-V-x-m-I-s-a-e-A-P-x-l-f-a-K-A-c-E-M-F-I-x-e-L-T-x-R-s-W-x-h-t-e-e-n-k-R-r-T-l-q-k-x-d-i-a-a-A-v-x-r-x-d-x-x-f-D-F-L-x-x-D-x-V-P, where the uppercase letters represent completely conserved sites, the lowercase letters except x represent relatively conserved sites, and the lowercase x represents non-conserved sites. This study characterized 24 NF-Y family members in peach by using the above three conserved regions to blast the peach protein database (version 2.0, https://www.rosaceae.org/blast/protein/protein) [16]. The candidate *NF-Y* genes were considered to be *PpNF-Y*s. The online program Conserved Domains (https://www.ncbi.nlm.nih.gov/Structure/) was used to ensure the conserved domains of the candidate NF-Ys [24]. PpNF-Y conserved motifs were identified using the MEME program (http://www. meme. sdsc.edu/meme/meme.html) [42].

### *PpNF-Y* sequence structure and genome distribution

The distribution of gene exons and introns was analysed using Gene Structure Display Server 2.0 software (http://gsds.cbi.pku.edu.cn/index.php) [14]. The detailed structures of the genes were drawn with Illustrator for Biological Sequences software (Version 1.0.3; http://ibs.biocuckoo.org/index.php) [22]. The locations of the *PpNF-Y*s on the genome were collected from a database (*Prunus persica* v2.1) in JGI (https://phytozome.jgi.doe.gov/jbrowse/index.html) [31]. Their genome distribution was displayed using the MapInspect tool (http://mapinspect.software.informer.com) [10].

### Calculating the parameters of *NF-Y* gene duplication events

The paralogous nucleotide sequences were pairwise aligned by MEGA 5.0 [40]. The parameters Ks (synonymous substitution rate) and Ka (nonsynonymous substitution rate) were calculated by the program DNASP 6.0 [34]. The date (*T*) of the duplication events was estimated by the formula *T* = Ks/2λ, where λ represents the estimated clock-like rate of synonymous substitution, which was 1.5 × 10^−8^ substitutions/synonymous site/year in dicots [4, 6].

### PpNF-Y sequence alignment and phylogenetic analysis

The PpNF-Y protein sequence alignments were analysed using MEGA 5.0 software (https://www.megasoftware.net) [40] and the conserved domains were marked using GeneDoc 2.7.000 software [30]. A phylogenetic tree of PpNF-Y family proteins was constructed using the neighbour-joining (NJ) method [35] of MEGA 5.0 software with a bootstrap test (*n*=1000).

### NF-Y protein sequences of *Arabidopsis*

The *Arabidopsis NF-Y* gene family was identified and numbered in a previous study [37]. The gene sequences and the corresponding protein sequences were collected by searching the gene names such as *NF-YA1* from the *Arabidopsis* Information Resource (TAIR) database (https://www.arabidopsis.org/) [3]. *Arabidopsis* NF-Y proteins were designated AtNF-Ys.

### Drought-responsive *cis*-elements of the *PpNF-Y* promoter

The promoter sequences (length, 1.5 kb) of *PpNF-Y*s were collected from the Genome Database for Rosaceae (https://www.rosaceae.org/). Drought-responsive *cis*-elements were analysed in the online software PlantCARE (http://bioinformatics.psb.ugent.be/webtools/plantcare/html/).

The models of *cis*-elements in the promoters were made with Illustrator for Biological Sequences software [22].

### RNA isolation and expression analysis

Total RNA from different tissues of *Prunus davidiana* (Pd) was extracted using TRIzol® reagent (Invitrogen Inc., USA). According to the supplier’s protocol, the extracted RNA was used as a template with M-MLV reverse transcriptase (Toyobo, Japan) to synthesize complementary DNA (cDNA). Real-time PCR was performed using a 7500 Fast Real Time PCR System (Applied Biosystems, NY, USA). One microlitre cDNA template, 10 μL SYBR Premix Ex Taq (Takara, Kyoto, Japan), 0.8 μL gene-specific primers, and 8.2 μL ddH_2_O were mixed to compose 20 μL reaction systems. The PCR thermal cycle was as follows: 95°C for 30 s; 40 cycles of 95°C for 5 s, 52°C for 30 s, and 72°C for 20 s. *Translation elongation factor 2* (*TEF2*) was used as an internal control to normalize gene expression [2, 43]. Each sample was analysed with three biological replicates.

### Statistical analysis

All data were subjected to analysis of variance according to Student’s *t* test using SPSS statistical software 17.0 (SPSS Inc., USA) [21].

## Result

### Identification of PpNF-Y family members

After removing different transcripts of the same gene, a total of 24 non-redundant protein sequences (6 PpNF-YAs, 12 PpNF-YBs and 6 PpNF-YCs) representing the primary transcript were identified. All 24 PpNF-Ys contained one highly conserved domain of the three NF-Y subfamilies that were also confirmed by the online database of Conserved Domains. To distinguish these newly identified genes, the current study renamed these genes based on subfamily branch and chromosomal distribution (*PpNF-YA1*-*PpNF-YA6*, *PpNF-YB1*-*PpNF-YB12* and *PpNF-YC1*- *PpNF-YC6*). The protein lengths of the 24 PpNF-Ys ranged from 121 AA to 398 AA (Table 1), showing a wide distribution of PpNF-Y lengths. Among the three subfamilies of PpNF-Y, the PpNF-YA lengths (range from 202 AA to 398 AA, average length 322.5 AA) were generally longest, the PpNF-YC lengths (range from 121 AA to 280 AA, average length 228.5 AA) were shorter, and the PpNF-YB lengths (range from 167 AA to 254 AA, average length 203.2 AA) were shortest. The predicted molecular weights (*Mw*) of the 24 PpNF-Ys ranged from 13.26 (PpNF-YC4) to 44.05 (PpNF-YA4), and the predicted theoretical isoelectric points (*pI*) of the 24 PpNF-Ys ranged from 5.27 (PpNF-YB2) to 9.43 (PpNF-YA5).

**Table 1:** Information of the *NF-Y* genes family in peach

Name	Gene	Chromosome localization	CDS	Length (AA)	Mw (kD)	PI	Homologs in *Arabidopsis*	Functional annotation in *Arabidopsis*
*PpNF-YA1*	*Prupe.2G014500*	Pp02:1285333..1293724+	1035	344	37.66	8.32	*AtNF-YA1,7*	Salt Stress [21]
*PpNF-YA2*	*Prupe.3G043900*	Pp03:3121596..3127378+	1032	343	38.00	9.20	*AtNF-YA3,8*	Embryo development [48]
*PpNF-YA3*	*Prupe.4G090200*	Pp04:4494268..4499893-	609	202	22.13	7.90	*AtNF-YA4,7*	Flower [11]
*PpNF-YA4*	*Prupe.4G249900*	Pp04:16710014..16714553+	1197	398	44.05	5.89	*AtNF-YA1,9*	Seed development [50]
*PpNF-YA5*	*Prupe.7G093100*	Pp07:12566137..12572248+	966	321	35.35	9.43	*AtNF-YA2*,*10*	Seed germination [41]
*PpNF-YA6*	*Prupe.7G203500*	Pp07:18875767..18879196+	984	327	36.37	8.81	*AtNF-YA3,8*	Embryo development [48]
*PpNF-YB1*	*Prupe.1G138500*	Pp01:10829440..10830284+	492	163	18.55	5.62	*AtNF-YB5,7*	Unknown
*PpNF-YB2*	*Prupe.2G219500*	Pp02:24828695..24832311+	543	180	19.19	5.27	*AtNF-YB1*	Drought stress [25]
*PpNF-YB3*	*Prupe.3G197300*	Pp03:20770866..20771306-	546	181	19.11	5.38	*AtNF-YB3*	Flowering, root growth [47]
*PpNF-YB4*	*Prupe.4G003800*	Pp04:258372..261155-	738	245	26.87	6.29	*AtNF-YB6*	Embryonic desiccation-intolerance [18]
*PpNF-YB5*	*Prupe.4G004200*	Pp04:271278..272705-	765	254	29.78	9.00	*AtNF-YB3*	Flowering, root growth [47]
*PpNF-YB6*	*Prupe.4G242700*	Pp04:16019863..16021262+	600	199	21.41	6.66	*AtNF-YB3*	Flowering, root growth [47]
*PpNF-YB7*	*Prupe.4G243500*	Pp04:16087429..16090365-	750	249	26.72	6.44	*AtNF-YB9*	Embryonic desiccation-intolerance [18]
*PpNF-YB8*	*Prupe.4G273800*	Pp04:22774061..22774753-	693	230	25.92	6.20	*AtNF-YB6*	Embryonic desiccation-intolerance [18]
*PpNF-YB9*	*Prupe.6G175100*	Pp06:17959164..17964486-	651	216	24.17	5.77	*AtNF-YB3*	Flowering, root growth [47]
*PpNF-YB10*	*Prupe.6G241800*	Pp06:24020199..24022272-	522	173	18.69	6.15	*AtNF-YB8*,*10*	Unknown
*PpNF-YB11*	*Prupe.6G323400*	Pp06:28504283..28505052+	519	172	19.11	5.41	*AtNF-YB4,5*	Unknown
*PpNF-YB12*	*Prupe.8G256000*	Pp08:21852595..21853514+	531	176	19.91	6.66	*AtNF-YB5*	Unknown
*PpNF-YC1*	*Prupe.1G152500*	Pp01:12042456..12046186+	783	260	28.87	5.89	*AtNF-YC9*	Flowering [37]
*PpNF-YC2*	*Prupe.3G149300*	Pp03:16308590..16312026+	636	211	24.53	5.37	*AtNF-YC2*,*3*	Flowering [37]
*PpNF-YC3*	*Prupe.4G132200*	Pp04:7318206..7321121+	843	280	31.40	5.76	*AtNF-YC2*,*3*	Flowering [37]
*PpNF-YC4*	*Prupe.5G096000*	Pp05:10523596..10524073+	366	121	13.26	7.82	*AtNF-YC3,9*	Flowering [37]
*PpNF-YC5*	*Prupe.5G150400*	Pp05:13652222..13654960+	714	237	26.04	5.31	*AtNF-YC4*	Floral induction [45]
*PpNF-YC6*	*Prupe.6G328500*	Pp06:28759728..28761956-	789	262	29.16	5.96	*AtNF-YC3,9*	Flowering [37]

### Phylogenetic relationships, genome distribution and gene structure of *PpNF-Y*s

**Fig. 1:**
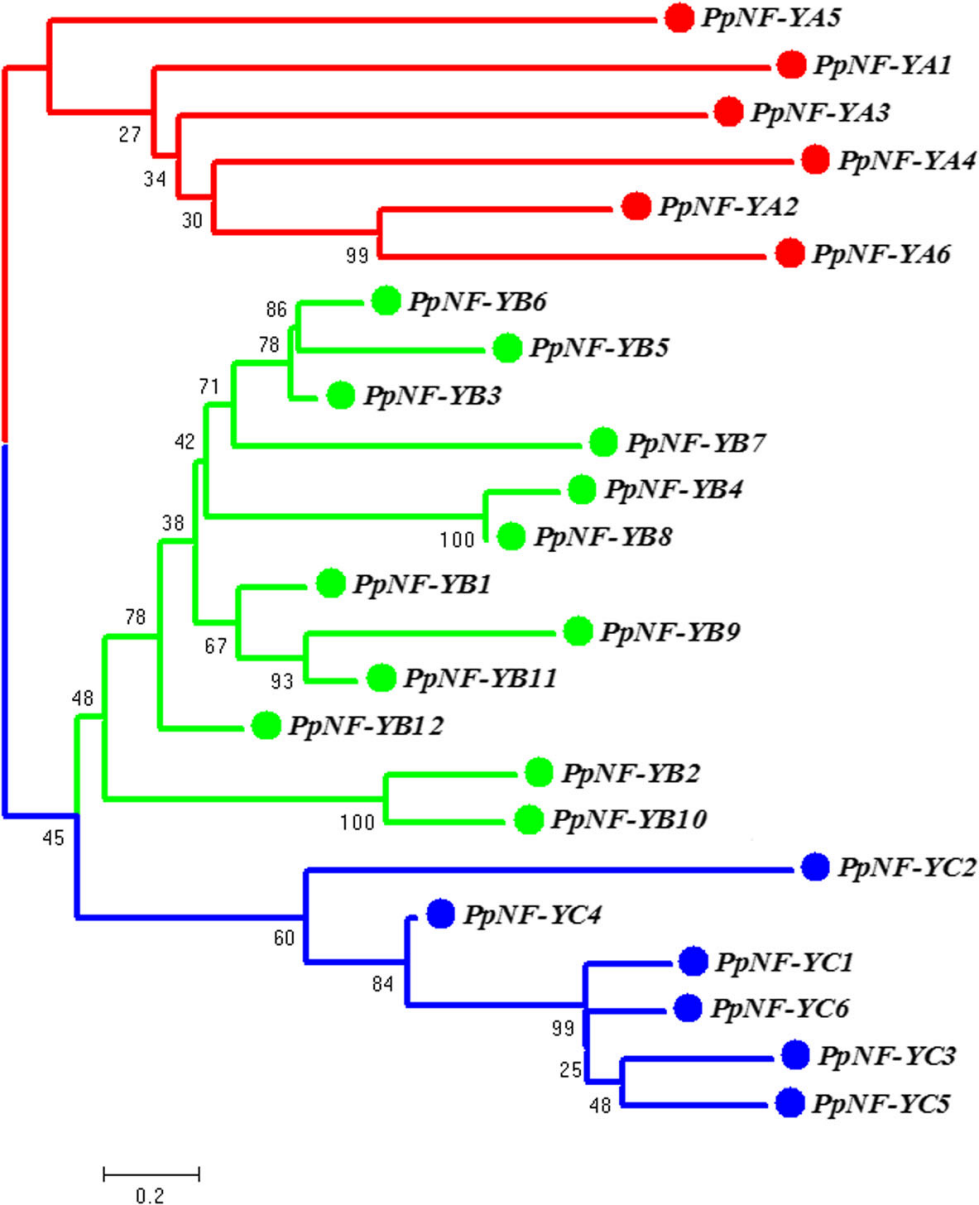
The phylogenetic relationship of the *PpNF-Y* gene family

To investigate the phylogenetic relationships among the *PpNF-Y*s, three phylogenetic trees were constructed based on an alignment of the *PpNF-Y* nucleotide sequences (Additional file 1) using MEGA 5.0 (Fig. 1). The neighbour-joining (NJ) phylogenetic trees were constructed to show the structural classification of the *PpNF-Y*s. As shown in Fig. 1, the NJ tree was distinctly divided into three subgroups, marked by three different colours (*PpNF-YA*s, red; *PpNF-YB*s, green; *PpNF-YC*s, blue), which were consistent with our identification results. The *PpNF-YA* branch had a simple structure, and a pair of paralogous *PpNF-YA*s (*PpNF-YA2* and *6*) was detected. In the *PpNF-YB* branch, there were four major groups. A group containing *PpNF-YB2* and *10* was located outside the three other groups. The presence of a bifurcation put *PpNF-YB12* outside a secondary subgroup formed by *PpNF-YB1, 9* and *11.* The third group was composed of *PpNF-YB4* and *8*. In the fourth group, there were also two bifurcations separating *PpNF-YB3* and *7* from a second subgroup composed of *PpNF-YB5* and *6.* The branch structure of the *PpNF-YC*s was similar to that of the *PpNF-YA*s. A pair of paralogous *PpNF-YC*s (*PpNF-YC3* and *5*) was located on the innermost side, and the remaining *PpNF-YC*s were located around the outside.

**Fig. 2:**
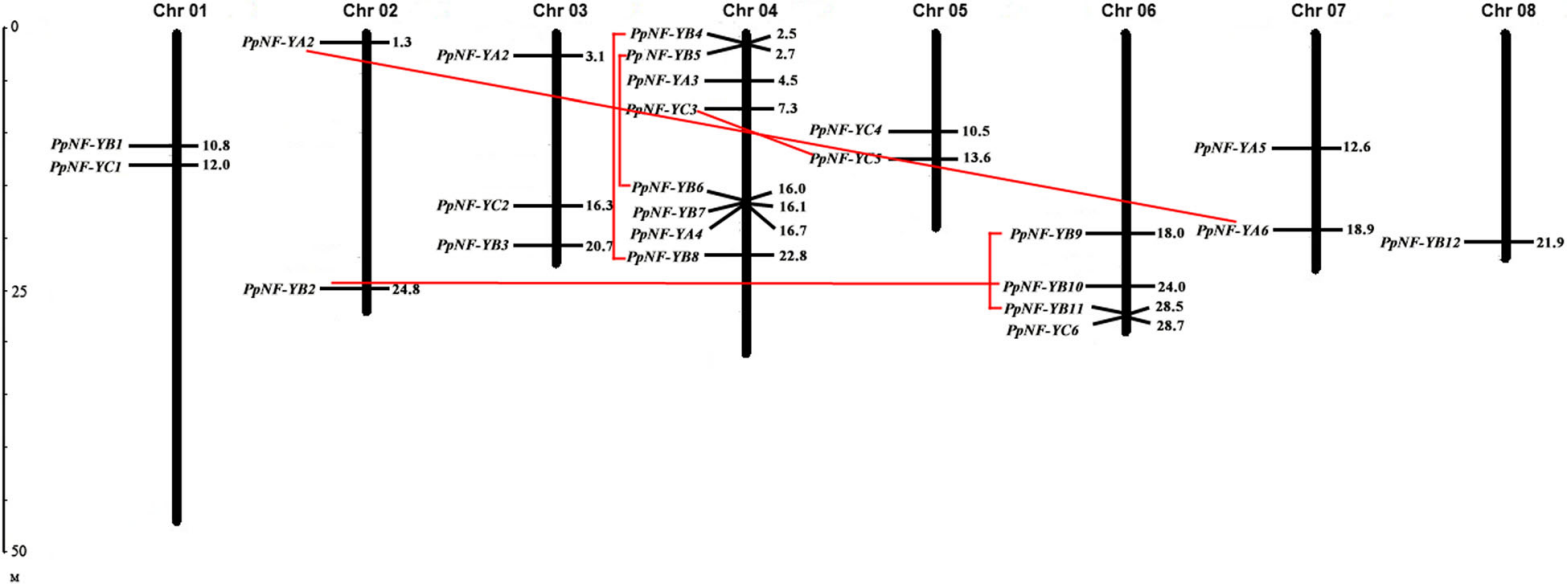
The distribution and duplication events of 24 *PpNF-Y*s in peach genome. The physical gene locations are drawn on the right side of the chromosome. The red lines connect the corresponding 6 pairs of paralogous genes

**Fig. 3:**
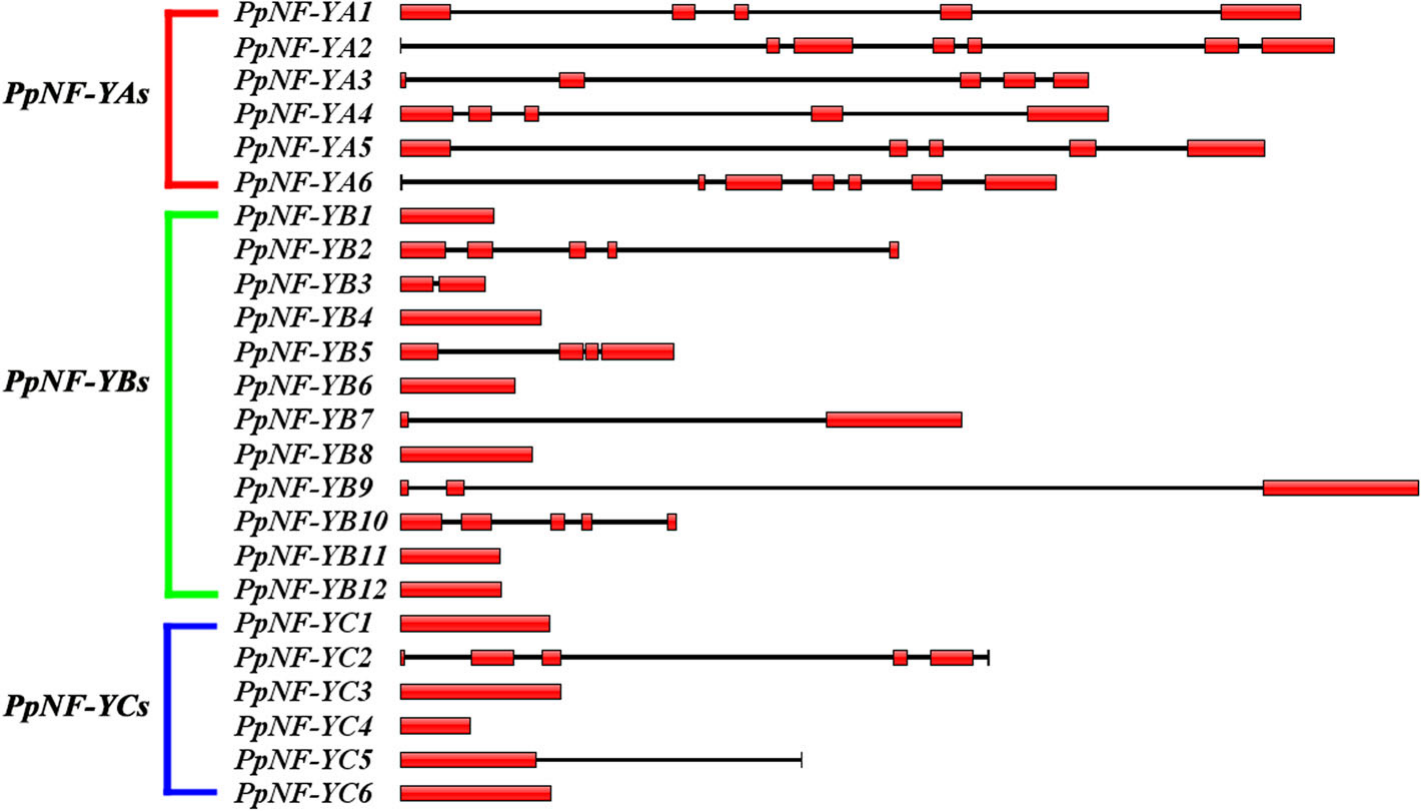
Gene structures of 24 *PpNF-Y*s. The red box represents exon and black line represents intron. The lengths of *PpNF-Y*s were shown in proportion according to their length

Twenty-four *PpNF-Y*s were distributed on all eight chromosomes (Fig. 2). Most *PpNF-Y*s with eight members were distributed on the 4th chromosome. The fewest of *PpNF-Y*s were located on the 8th chromosome, with only one member. The other six chromosomes contained 2-4 *PpNF-Y*s. The exons and introns of the *PpNF-Y*s were drawn with Illustrator for Biological Sequences (IBS, version 1.0.3) software using the genomic DNA sequences of the *PpNF-Y*s and the corresponding coding sequence (Fig. 3). All *PpNF-YA*s contained 4-6 introns. Some *PpNF-YB*s (*PpNF-YB1*, *4*, *6*, *8*, *11*, *12*) and *PpNF-YC*s (*PpNF-YC1*, *3*, *4*, *6*) were composed of only one exon.

### Duplication events of the *NF-Y* genes in the peach genome

In the phylogenetic analysis of the *NF-Y* gene family, 6 pairs of paralogous *NF-Y* genes were detected (Fig. 2). Among them, 3 pairs (*PpNF-YA2* and *PpNF-YA6*, *PpNF-YB2* and *PpNF-YB10*, *PpNF-YC3* and *PpNF-YC5*) were randomly scattered on different chromosomes, and the other three pairs (*PpNF-YB5* and *PpNF-YB6*, *PpNF-YB4* and *PpNF-YB8*, *PpNF-YB9* and *PpNF-YB11*) were located on the same chromosomes. To further trace the dates of the duplication events, the parameters Ks and Ka and the Ka/Ks ratio were estimated using DNASP 6.0 software [34]. The Ka/Ks ratios of *PpNF-YB9* and *PpNF-YB11* were greater than 1 and those of the other five pairs of paralogous *PpNF-Y*s were less than 1. The approximate dates of the duplication events are shown in Table 2. The origin dates of the three pairs of paralogous *PpNF-Ys* on the different chromosomes ranged from 20.6 to 59.67 million years ago. The dates of the other three pairs on the same chromosomes ranged from 13.33 to 68.33 million years ago.

**Table 2:** The parameters and dates of the duplication evens in the paralogous *PpNF-Y*s.

The paralogous gene		KS	KA	Ka/Ks ratio	Date (million year ago)
*PpNF-YA2*	*PpNF-YA6*	0.66	0.55	0.83	22.00
*PpNF-YB5*	*PpNF-YB6*	1.08	0.21	0.19	36.00
*PpNF-YB4*	*PpNF-YB8*	2.05	0.43	0.21	68.33
*PpNF-YB9*	*PpNF-YB11*	0.4	0.55	1.38	13.33
*PpNF-YB2*	*PpNF-YB10*	0.62	0.28	0.45	20.67
*PpNF-YC3*	*PpNF-YC5*	1.79	0.22	0.12	59.67

Ks and Ka were calculated using the program DNASP 6.0 [34]. The date of the duplication events in the paralogous *PpNF-Y*s were analzyzed by the formula *T* = Ks/2λ, λ representing the estimated clock-like rates of synonymous substitution, which was 1.5 × 10^−8^ substitutions/synonymous site/year in dicots [4]

### Conserved regions of PpNF-Ys

**Fig. 4:**
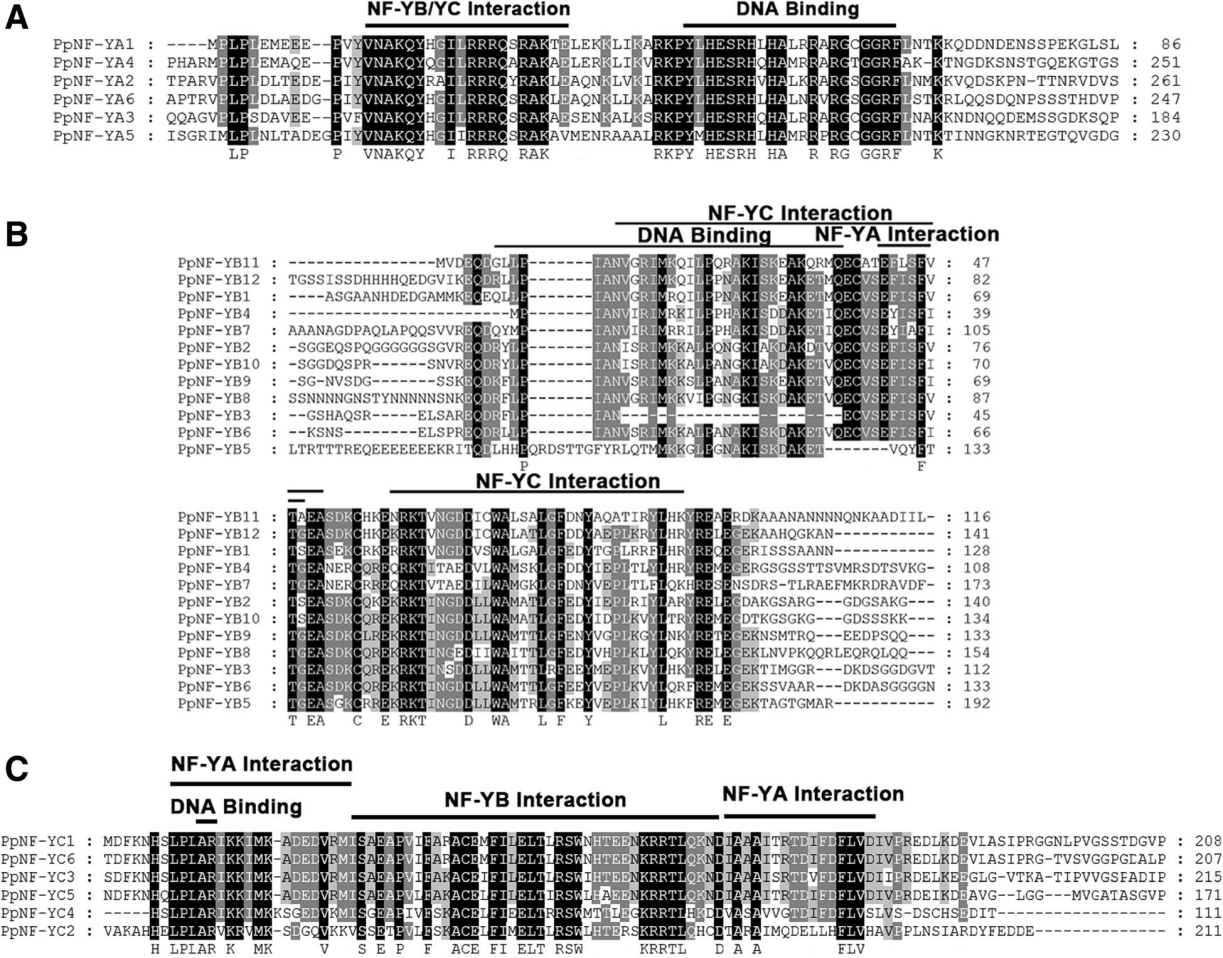
The details of conserved domians in the three subfamilies of PpNF-Ys Subunit interaction and DNA binding regions of conserved domians are marked. The completely conserved amino acids are colored by black boxes and labelled in the last line. The relatively conserved amino acids (80%-100%) are colored by brown boxes. The last number of each line represents position of the last amino acid in its whole protein. **a**. PpNF-YA subfamily; **b**. PpNF-YB subfamily; **c**. PpNF-YC subfamily

To further investigate the conserved regions of the three subfamilies in peach, multiple protein sequence alignments of the PpNF-YAs, PpNF-YBs, and PpNF-YCs were analysed using MEGA 5.0. All three subfamilies contained conserved regions. Multiple alignment of the PpNF-YA proteins indicated that there was a conserved region composed of approximately 50 amino acids (Fig. 4a). As with the PpNF-YAs, two conserved regions of PpNF-YB (approximately 102 amino acids) and PpNF-YC (approximately 81 amino acids) were identified by protein sequence alignment (Fig. 4b and c).

**Fig. 5:**
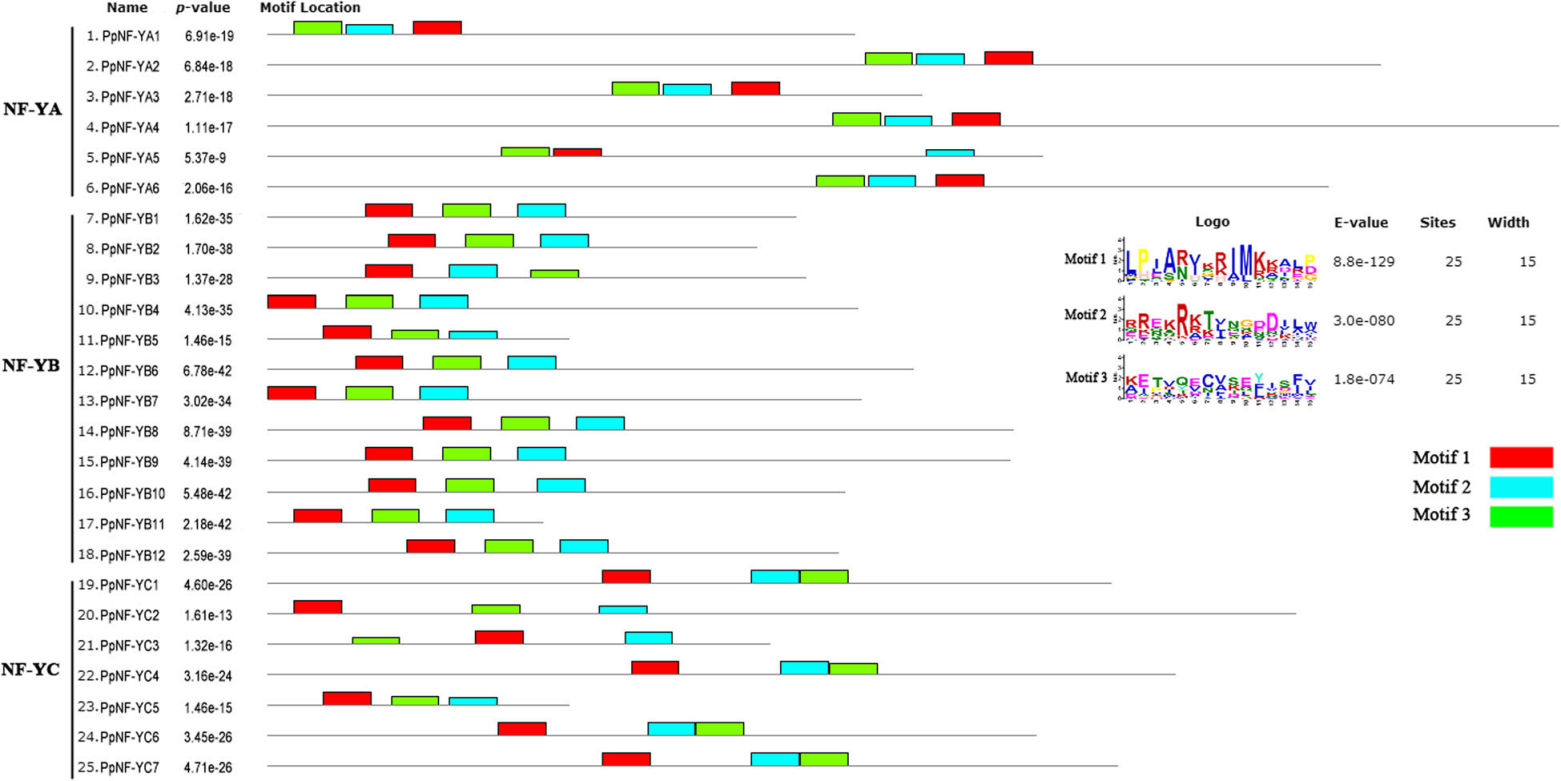
Schematic diagram of motif structure in the peach *NF-Y* gene family using MEME Three conserved motifs are shaded in different colors. Three subfamilies are distinguished by the motif distribution

To reveal the putative motifs of the *NF-Y* family in peach, 24 *PpNF-Y*s were analysed using the program MEME. All members contained three distinct motifs (Fig. 5), which was consistent with the fact that they belonged to the same gene family.

### Expression patterns of *PpNF-Y*s in different peach organs

**Fig. 6:**
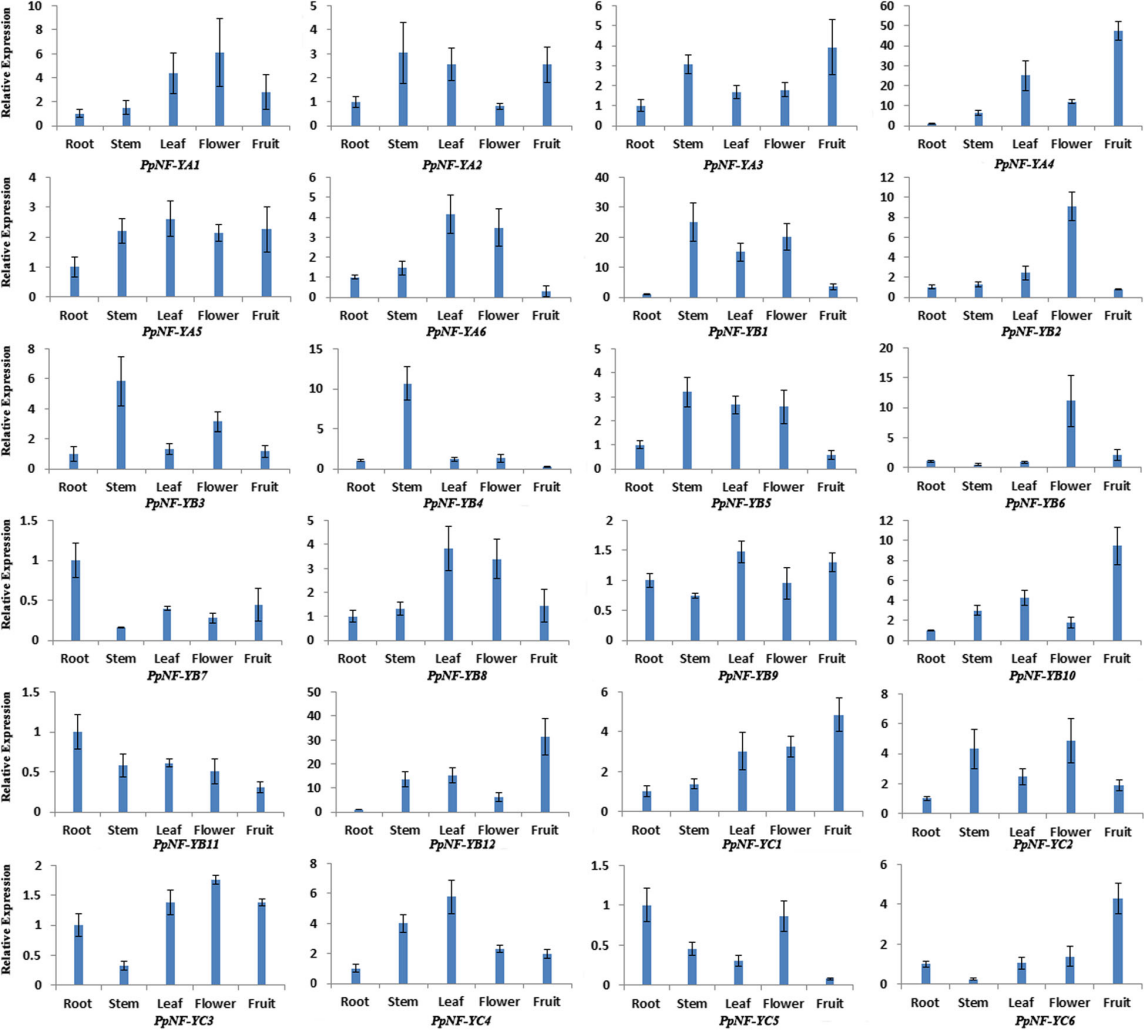
Expression patterns of *PpNF-Y* genes in five peach organs. The x-axis represents different peach organs. The y-axis represents the relative expression levels of *PpNF-****Y*** genes compared with actin. The expressions of *PpNF-****Y*** genes in root were normalized to 1. Error bars represent standard deviations for three replicates

The expression patterns of *PpNF-Y*s in the five organs (roots, stems, leaves, flowers and fruits) were analysed (Fig. 6). The *PpNF-Y*s showed different expression patterns. Some were constitutively expressed in every organ such as *PpNF-YB5*, while others showed specific high expression in one or two peach organs, such as *PpNF-YA4* (leaves and fruits), *PpNF-YB6* (flowers) and *PpNF-YB4* (stems). This diversity of expression patterns of *PpNF-Y*s suggested a divergence in the biological functions of *PpNF-Y*s during peach growth and development.

### Evolutionary relationships of the *NF-Y* family in peach and *Arabidopsis*

**Fig. 7:**
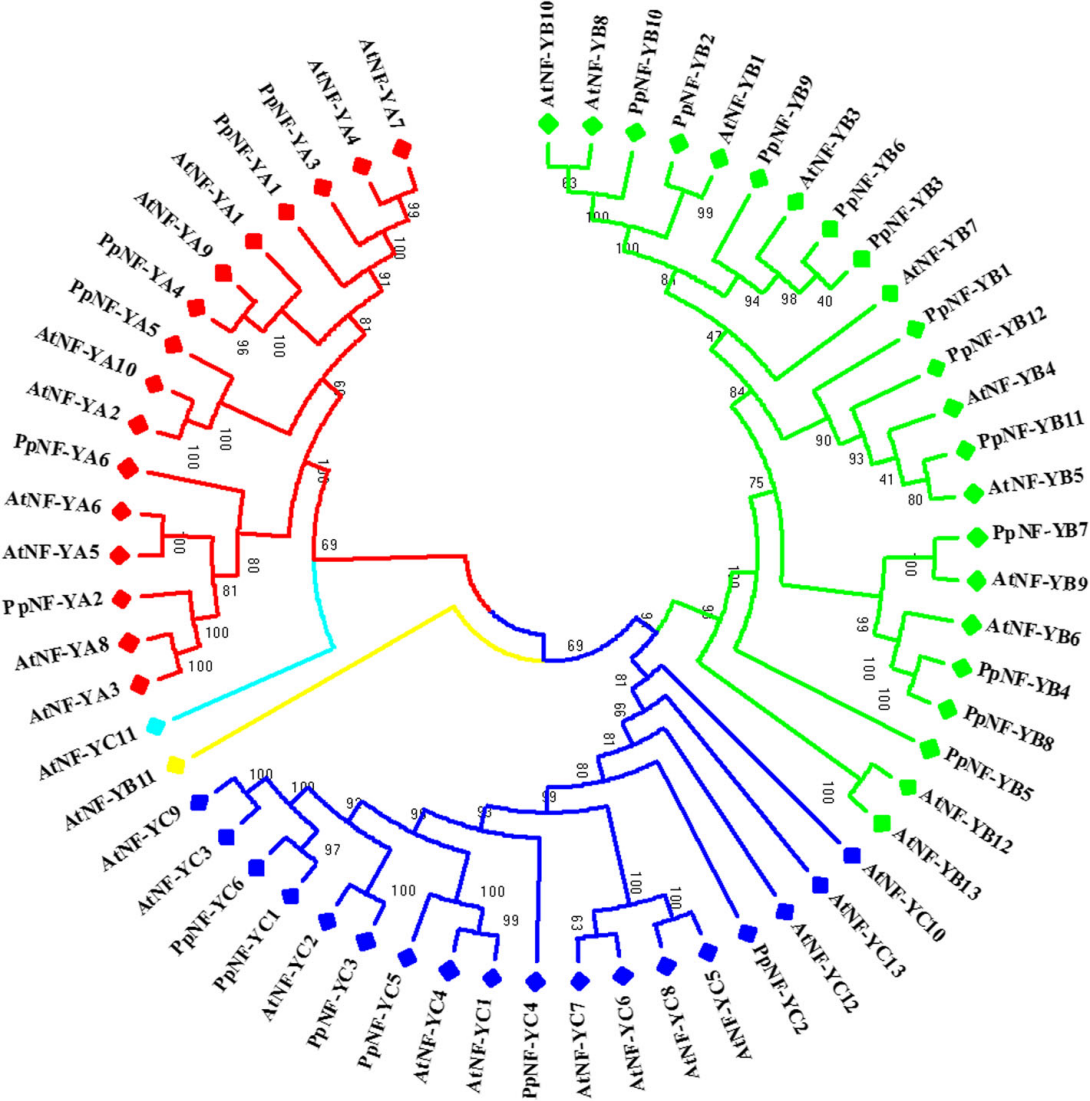
Phylogenetic relationships of NF-Y proteins in peach and Arabidopsis Three subfamilies are shown in different colored

The functions of some *AtNF-Y* family members have been identified in *Arabidopsis*. However, the biological functions of PpNF-Y proteins are unknown. To predict the functions of PpNF-Y members, we explored the phylogenetic relationships of the NF-Y members from *Arabidopsis* and peach using 60 NF-Y protein sequences (Fig. 7; Additional files 2 and 3). The NJ phylogenetic tree showed that 58 NF-Y members (excluding AtNF-YB11 and AtNF-YC11) could be classified into three main groups distinguished by three different colours (red, green and blue), which were consistent with the subfamily classifications of the PpNF-Ys. In each group, we found that some pairs of paralogous NF-Y proteins were composed of one PpNF-Y and one AtNF-Y, such as PpNF-YA4 and AtNF-YA9, and this close evolutionary relationship generally suggested the similarity of their biological functions. Both the NF-YA and NF-YC groups contained one pair of paralogous NF-Y proteins, and the NF-YB group contained three pairs.

### Analysis of five drought-responsive *cis*-elements in the promoter sequences of *PpNF-Y* genes

**Fig. 8:**
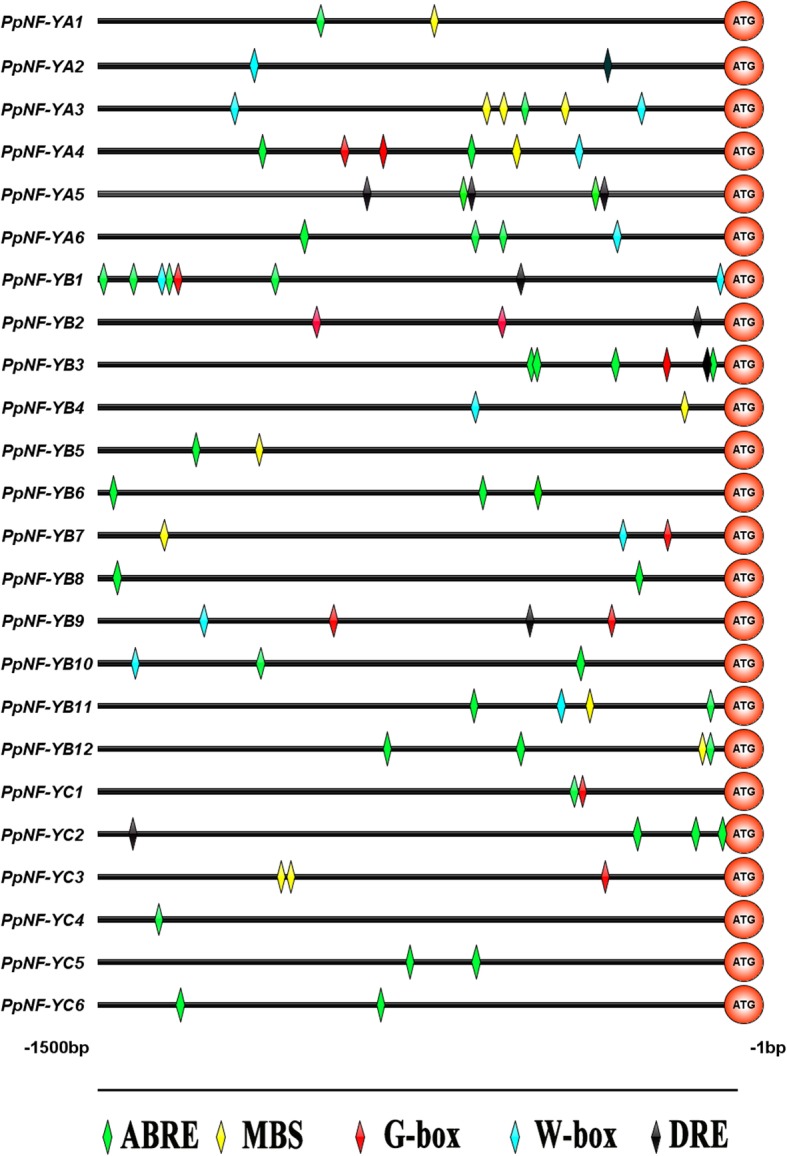
Schematic model of five drought-responsive *ci*s-elements in the promoter sequences of *PpNF-Y* genes. MBS represents MYB binding site involved in drought-inducibility *ci*s-element; ABRE represents *ci*s-acting element involved in the abscisic acid responsiveness; DRE represents dehydration responsive element

To explore the involvement of the *PpNF-Y* genes in drought tolerance, their promoter sequences were analysed using PlantCARE software [19]. It is generally known that five *cis*-elements, ABREs, MBSs, G-boxs, W-boxs and DREs, respond to drought-induced signalling and regulation of downstream gene expression [18, 26, 36]. All five *cis*-elements in the promoter regions of the *PpNF-Y* gene family members are shown in Fig. 8. The results indicated that all 24 *PpNF-Y* promoter regions contained one or more drought-responsive *ci*s-elements. ABREs, MBSs, G-boxes, W-boxes and DREs were distributed within 17, 9, 8, 10 and 7 *PpNF-Y* promoter regions, respectively. The numbers of drought-responsive *ci*s-elements in the 24 *PpNF-Y* promoter regions ranged from 1 (*PpNF-YC4*) to 8 (*PpNF-YB1*), and the types ranged from 1 (*PpNF-YC4*) to 4 (*PpNF-YB1*).

### Expression analysis of *PpNF-Y*s under drought stress

**Fig. 9:**
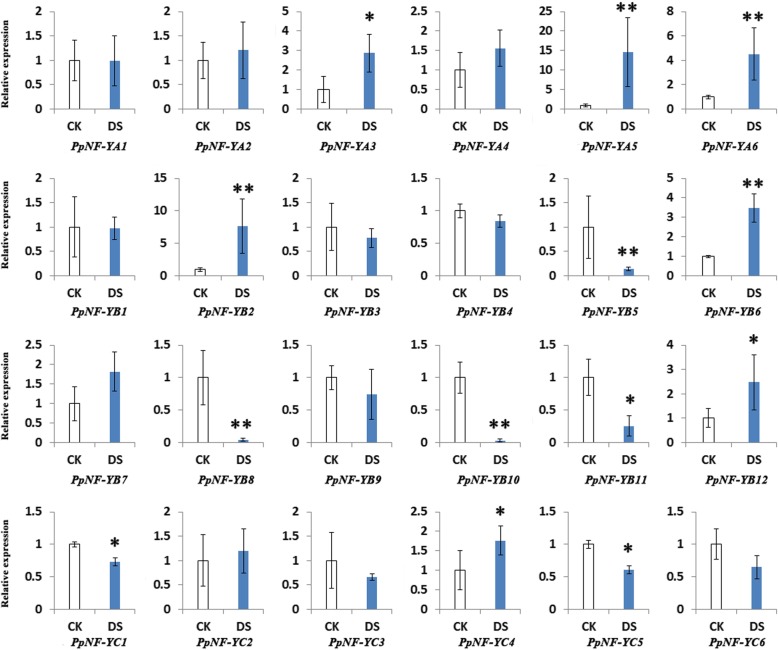
Relative expression analysis of the 24 *PpNF-Y*s from Pd (*Prunus davidiana*) seedlings under drought stress was performed. Data presented are the means±SD of three replicates. CK represents seedlings without drought stress. DS represents seedlings under drought stress

Nelson et al. [28], Chen et al. [8] and Pereira et al. [33] have reported that the *NF-Y* gene family is closely related to drought tolerance in some species, including *Arabidopsis*, *Citrus*, maize, and Bermuda grass. In peach, it is possible that one or more *PpNF-Y*s are involved in tolerance to drought stress. To investigate drought-responsive *PpNF-Y*s, an expression analysis of the 24 *PpNF-Y*s from Pd seedlings under drought stress was performed (Fig. 9; Additional file 4). The results showed that 4 *PpNF-YA*s (*PpNF-YA*3, 4, 5, and 6), 4 *PpNF-YB*s (*PpNF-YB*2, 6, 7, and 12) and *PpNF-YC4* were upregulated under drought stress. Among these members, the expression level of *PpNF-YA5* increased the most, to approximately fifteen times that of the control sample. The upregulated gene *PpNF-YA4* and *PpNF-YB7* showed the smallest increases in transcript abundance, less than two times that of the control. The increased expression of the other six upregulated *PpNF-Y*s ranged from two to eight times that of the control.

## Discussion

### Peach contains only 24 diverse *NF-Ys*

The *NF-Y* TFs are found in all sequenced eukaryotes. This gene family has been studied in some higher plant species, such as maize, rice, tomato, and banana [20, 46, 47, 51, 52]. Mantovani [23] has revealed some properties of this family, including relatively conserved binding and interaction domains, three types of subunits, and diverse biological functions, which were beneficial to our research on the *NF-Y* gene family in peach. A total of 24 *NF-Y* members were identified in peach, fewer in *Arabidopsis* (36 *NF-Y* members), tomato (59 *NF-Y* members), rice (34 *NF-Y* members), and wheat (39 *NF-Y* members). The small number of peach *NF-Y* members may be associated with the small size of the peach genome. The 24 *PpNF-Y*s demonstrated diversity in several aspects, including protein length, gene structure, molecular weight, theoretical isoelectric point and expression pattern, which suggested a diversity of biological functions among *PpNF-Y*s.

### Putative segmental and tandem duplication events in the peach genome

The paralogous genes distributed on different chromosomes are generally designated as segmental duplication events, and those co-located on the same chromosome are considered tandem duplication events [6]. In this study, the locations of 6 paralogous *NF-Y* genes were either on the same chromosome or on different chromosomes, implying that both tandem and segmental duplication events contributed to the expansion of the *NF-Y* genes in peach. When the Ka/Ks ratios of the five paralogous *PpNF-Y*s were less than 1, purifying selection of duplication events occurred and the corresponding paralogous PpNF-Y proteins were considered to be unchanged. In contrast, the Ka/Ks ratios of *PpNF-YB9* and *PpNF-YB11* were greater than 1, indicating that distinct variation between the PpNF-YB9 and PpNF-YB11 proteins occurred during the duplication event. As the initial point of peach evolution was still unclear, we could not determine whether the duplication events in this gene family predated the formation of the peach species. However, the oldest date of the duplication events among the 6 pairs of paralogous *PpNF-Y*s could be 68.33 million years ago, suggesting that this is an ancient gene family. At a minimum, it could indicate traces of peach evolution.

### Potential conserved domains in three NF-Y subfamilies

In general, proteins can be characterized and classified by their conserved regions, which play vital roles in heterodimerization, heterotrimerization, and DNA interactions at *CCAAT* sites (Zambelli and Pavesi 2017). Previous studies have shown that all three subfamilies (NF-YA, NF-YB and NF-YC) are recognized by interaction domains that interact with NF-Y members and DNA-binding domains that bind to downstream targeted *CCAAT* sites [25]. Based on the recognized DNA-binding domain of NF-YA in plants, mammals and yeasts, the similar 17-amino-acid sequence Y-L-H-E-… -G-G-R-F in the C-terminus of the PpNF-YA conserved region was considered to interact with DNA at *CCAAT* sites (Fig. 4a). It was inferred that the 21-amino-acid sequence Y-V-N-A- … -A-K-L-E in the N-terminus of the PpNF-YA conserved region interacted with the other two subunits (PpNF-YB and PpNF-YC) (Fig. 4a).

The structure and amino acid composition of the NF-YB conserved regions were similar to those of H2B histone fold motifs [12]. Based on the conserved regions of NF-YB members in *Arabidopsis*, a 31-amino-acid sequence R-x-L-P-… -E-T-x-Q was considered to be the DNA-binding domain of PpNF-YB. Similarly, two core regions, the 40-amino-acid sequence A-N-V-x-… -T-x-E-A and the 32-amino acid sequence x-R-K-T-… Y-L-x-x, were considered to interact with the other two subunits (PpNF-YA and PpNF-YC) (Fig. 4b).

In the alignment analysis of 7 PpNF-YC members, the conserved 74-amino-acid sequence L-P-L-A-… -D-F-L-V had similarities to an *Arabidopsis* subunit interaction domain (Fig. 4c). Thus, we inferred that this fragment was the core region by which PpNF-YC associated PpNF-YA/PpNF-YB. In addition, the DNA- binding domain of PpNF-YC was composed of only two residues in series “A” and “R”, which were necessary for the formation of a complex between heterotrimeric NF-Y and DNA.

### Similar core protein regions between PpNF-Ys and AtNF-Ys imply similar biological functions

Based on the analysis of the protein alignment, the conserved core amino acids of the PpNF-YA interaction and DNA-binding domains were almost highly consistent with those of AtNF-YAs, RnCFB-B (Rn, *Rattus norvegicus*), ScHAP2 (Sc, *Saccharomyces cerevisiae*), indicating that there may be similar biological functions of several *NF-YA* members among plants, mammals and yeasts. For example, most *AtNF-YA*s increased their expression under drought stress in *Arabidopsis* [13], and two-thirds of *PpNF-YA*s were also upregulated under drought stress in this study.

Boulard et al. [5] showed that the substitution of a required amino acid residue (from Lys to Asp) caused LEC1 (AtNF-YB9) and LEC1-like (AtNF-YB6) to differ from plant and animal NF-YB proteins, and this variation caused LEC1 to fail to rescue the *lec1* embryonic desiccation-intolerant phenotype. The alternating distribution of PpNF-Y and AtNF-Y proteins in the NJ tree implied that some NF-Y members from these two species might originate from common ancestors, which might be reflected in their protein sequences. For example, we found that two PpNF-YB proteins, PpNF-YB4 and PpNF-YB7, were closely related to AtNF-YB9 and AtNF-YB6, and these four members together formed a subgroup in the NF-YB branch, which might suggest a similar biological function for them. Specifically, a substitution of required amino-acid residues (from Lys to Asp; the column marked by *) emerged in PpNF-YB4 and PpNF-YB7 (Fig. 4b), indicating that these two PpNF-YB members may have similar biological functions to those of AtNF-YB9 and AtNF-YB6. In *Arabidopsis*, AtNF-YB members have been shown to have biological functions in various developmental processes, such as stimulating cell division and expansion, promoting flowering, and synthetizing chloroplasts [39]. Based on the phylogenetic relationships of NF-Y proteins between *Arabidopsis* and peach, more biological functions of PpNF-YB members could be identified in various developmental processes.

### Diverse expression patterns of *PpNF-Y*s indicated a diversity of biological functions

As gene expression patterns can provide important clues for gene function, the current study analysed the expression patterns of *PpNF-Y*s in five peach organs. Some *PpNF-Y*s such as *PpNF-YB* and *PpNF-YB*7, were highly expressed in vegetative tissues, indicating that they might be involved in vegetative growth. Some *PpNF-Y*s such as *PpNF-YC1*, were highly expressed in both vegetative tissues and reproductive tissues, showing that they might play multiple roles in the developmental process. However, some *PpNF-Y*s such as *PpNF-YB10* and *PpNF-YC6*, were specifically expressed in reproductive tissues, implying that they might participate in the development of floral organs and fruits. The expression patterns of *PpNF-Y*s can reveal the behaviours of *PpNF-Y*s during peach growth and development, providing useful information for further identification of the biological functions of *PpNF-Y*s.

### *PpNF-Y*s as candidate drought-tolerance genes

The *NF-Y* TFs are closely related to drought stress tolerance. *NF-Y* members, including *Arabidopsis NF-YA5* and *NF-YB1*, maize *NF-YB2*, soybean *NF-YA3*, poplar *NF-YB7*, and bermudagrass *NF-YC1*, have been identified to participate in tolerance to drought stress [8, 29, 49]. Moreover, the transcriptional expression of drought-tolerance genes is generally induced by drought stress. This character could be used for identifying unknown drought-tolerance genes in peach.

The analysis of drought-responsive *cis*-elements in the *PpNF-Y* promoter sequences implied that some members might be involved in the drought-responsive pathway. The *PpNF-Y* promoter regions containing multiple types of drought-responsive cis-elements suggested that *PpNF-Y*s might be involved in different drought-responsive pathways. Based on an analysis of gene expression under drought stress, 9 upregulated *PpNF-Y*s could serve as candidate genes to analyse drought tolerance in peach. The NJ tree of PpNF-Y and AtNF-Y proteins showed a close relationship between AtNF-YB1 and PpNF-YB2, which was consistent with our inference that *PpNF-YB2* might be a drought-resistance gene, similar to *AtNF-YB1*. *PpNF-YA5*, another candidate drought-resistance gene, also showed a close relationship with the drought-resistance gene *AtNF-YA5*, supporting our analysis and prediction. This study provided the possibility to further research the novel drought-tolerance pathway related to the *NF-Y* genes in peach.

### The *NF-Y* gene family is a popular research topic

The strong correlation between the *NF-Y* gene family and drought resistance has become a popular research topic and has been demonstrated in many species. Recently, in *Citrus*, castor bean and chickpea, *NF-Y* gene family has been identified and candidate drought resistance genes members were analysed. Compared with recent published studies, this study contained some unique insights. First, the analysis of the *PpNF-Y* gene duplication indicated that most gene duplication events occurred long ago and generated similar duplicate genes, providing important clues into the evolutionary origins of the *PpNF-Y* gene family. Second, the details of drought-responsive *cis*-elements in the *PpNF-Y* promoters were shown, which is useful for exploring the upstream elements of the *PpNF-Y* genes involved in the drought resistance pathway. Third, the results of the phylogenetic relationships of *NF-Y* proteins between *Arabidopsis* and peach suggested that a continued targeted functional analysis of the *PpNF-Y* genes could be performed, such as for *PpNF-YB2*-*AtNF-YB1* (drought resistance) and *PpNF-YB4*-*AtNF-YB9* (desiccation-intolerant phenotype).

## Conclusions

Since the *NF-Y* gene family was first recognized and classified, many studies have focused on this gene family. Recently, published studies have identified the *NF-Y* gene family in various plant species including maize, tomato, rice, banana and analysed the functions of this gene family members in some processes such as fruit ripening and abiotic stress. The current study found that *NF-Y* gene family members were frequently identified in field crop and vegetable crop species, and this research trend has been extended to fruit trees. Our study, carried out in peach, closely followed the current research focus. Twenty-four *PpNF-Y*s were first identified in the peach genome in the current study. Each subfamily of the *PpNF-Y*s contained typical characteristics. The analysis of the duplication events clearly displayed the expansion of *PpNF-Y*s across the genome. The current study indicated that there were some structural similarities of *NF-Y*s between peach and *Arabidopsis*. The reported *AtNF-Y*s were useful to predict and analyse the biological functions of the peach *NF-Y* gene family. In particular, the current study predicted the functions of some *PpNF-Y*s such as *PpNF-YB4*-*AtNF-YB9*, *PpNF-YB2*-*AtNF-YB1*, with the help of similar conserved domains and close evolutionary relationships to the reported *AtNF-Y*s, which allowed detailed research into those *PpNF-Y*s. In addition, the identification of drought-responsive *cis*-elements in the promoter regions of *PpNF-Y*s was useful to analyse and extend the drought-resistance pathway in peach. This study explored the performance of *NF-Y*s under drought stress in a notable current research area. Moreover, the current study identified two *PpNF-Y*s (*PpNF-YB2* and *PpNF-YA5*) as candidate genes for drought resistance, providing a foundation for further investigating the functions of *PpNF-Y*s and the molecular mechanism of peach drought-resistance.

## Additional files

Additional file 1: Peach NF-Ys gene sequence. (DOCX 32 kb)

Additional file 2: Peach NF-Ys protein sequence. (DOCX 18 kb)

Additional file 3: Arabidopsis protein sequence. (XLSX 11 kb)

Additional file 4: Primer of Peach NF-Y family. (DOCX 19 kb)

## Data Availability

Additional file 1. Peach NF-Ys gene sequence Additional file 2. Peach NF-Ys protein sequence Additional file 3. Arabidopsis protein sequence Additional file 4. Primer of Peach NF-Y family

## Abbreviations

μL: Microlitre
ABRE: Abscisic Acid Responsive Element
AtNF-Y: *Arabidopsis* NF-Y
CaNF-Y: *Cicer arietinum* NF-Y
cDNA: Complementary DNA
CsNF-Y: *Citrus sinensis* NF-Y
DNA: Deoxyribonucleic acid
IBS: Illustrator for Biological Sequences
Kb: Kilobase
MBS: MYB binding site involved in drought-inducibility
mRNA: Messenger RNA
Mw: Molecular weight
NF-Y: Nuclear Factor Y
NJ: Neighbour-joining
Pd: *Prunus davidiana*
Pi: Isoelectric point
PpNF-Y: Peach NF-Y
RcNF-Y: *Ricinus communis* NF-Y
RNA: Ribonucleic acid
