# Supplementary material for: Genome-wide analysis of the NF-Y gene family in peach (Prunus persica L.)
Source: BMC Genomics. 2019 Jul 26;20:612. doi: 10.1186/s12864-019-5968-7 (PMC6660701; doi:10.1186/s12864-019-5968-7)
Supplement: Supplementary file 1 — Peach NF-Ys gene sequence. (DOCX 32 kb) [file 12864_2019_5968_MOESM1_ESM.docx]

>PpNF-YA1

ATGATGCCAGCTAAATCTAAAGATGAAGATCCACATATAGAACACGGTGCCCAGACTGTGCTGGAATCAGCTATCTACGCCCAACCTTGGTGGCGTGGAGTGGGGAACAATTCATCGTCAGGTGAAAGTGCTCCAGCATCATCTTTGGTGGATCATCGTGATAGTATGGTCATGAATGGAGCTATGCAGTCACAAGCTAATGCTAGGCTGGATGGTGGGGCCAACTTCAACAAAGAATTGCGGACTACAGGAGGATCACAGTCTGGTAGTTACTTTTGCATTATCATTAAAATTTCATGTGAACTTCTTTTTCTGAATTGGAAATTTTCACACTTGCTACTTTAGAAATTAACTATAAAGTAATCATTCTGATAAACCCTTTTAAAATCTTATTTTATTTTATTTATAATTATTTACTAAGCCTTTGTCTTCTTTGTGAGATTGAAGGACCGAATGCATCATCAATTTGACATCCTGACCAAGACCTTTCAATCTGTGAAATAAAAGGAAATTTGAAGGTTTTTATAATTGTGATAGTGCTTATGATCTAAACTGAATAGAACTTGATACAACTAAGACTAGCATTTGTTGGAGAAAAAAGAATTAAAATAGGCCTTAGACACCTGTGGTTAGTTATTACAATCTTTCTCTTACTAGCTTCTTGTGTGGTGGGCTAATTTGTATATGGAAAACTAAATCTAGTTGAGAAAAAAGGAACTGCATTAAGAAGGTGAGAATCCTTGTTACTTTCCCTGTGTGTGTTTTGGGTGGGGTAGGGTGGGGAACTAGAAGTTGTGCAATTGCTATGGATTTTATCTTGCTCTTATTTCAATTCTGTAGTATGGATTTGATTTCTAGAAAAAGAGACCTGAAACCAAGCAAATTCACTTGTAAAATTCCCTGAACCATCCTTGACACCTTGCTCAGCAAACCCAAATTAACCCTTCCCTGATAGACATAGTGCTGAATCTGAAGCACCTGTATCAGATTTTCCCAGAGCAATCTACTTTATGTCAAAAGGTCTCCTAAAATCGACACTCGCTCCAATGGACTTATTGTTTTTCTTCTTCTTGACAATGCTGCCACTCTGCTTGTTTTCTGCCATGAGCATCTAAGCTGGCGACGTTGGTGTCTCGGGGCAGCATGCAGAAGAAAATATAATTTTTTAATATTAAATGTATAAATGCCTGCAGCCTAAGATATGTGAGCTTCAAAATAAATCTTGACGTAAGCTCCTATTGTACATGGAATGCTTCAATTCTTTTAAGTGGATTGGTTATTATATCTGGTTGATAATACTTGTTAGCACTATGGAATATGTCTGCTCCAAGTCTTCCCTGTAACATTTCCAGTGTTATATCTTCTTTCATATATTAACTCAACTGCCCATATACTTATTAATGCAAGTTTCCTTTTATTCAGACCAGAAAAATGGATGCAAAAACCAGCAAATCAAAAGCGTTTCTTCCTCGGTGGTTCCTACAATGGGTGAACACCTCGATCCAAATTCACAAATGGAACTTGTTGGTCACTCAATCGTAAGTACTTTCTCTTTCCCTCTTGCATTCTACATATATAAACCTTCAATACATGGAAACAATCACAGAAATTTGAACAGCAAGAATTTGTATCCATTACTATTAAAGACATGCAAATTTCTTGTGGAAAAAGTTCTGTTGTTCAACTTATGGTTTTCATTCGCTGTATGGGGTTATGCATAGACAGTTTACAAGTCTCATTAAATTTTGCAGGTTTTGACATCATATCCATATTCGGATCCACAATATGGCGGGATGTTGACTCCTTATGGGGCACAAGCTATGGTATGCAATCTGCAGTAATATATATTGTTGCCGTAGTATATGTTGTATCCAAAACTTCCCCCCAAGTTAGAGGATAGAGGATTATTATTCTCAGCTTGTTAAATTACTCGGAAGTATGATTAAGAACTAAAAAACTTAACTCTTCCTTTTCTTAATGAAGAGTTCTGTTTCTTATATAAAAAGAGAAACATAAAACAAATTAAAATACATTAAGCCTCAACTTTCTTTGAATGTGCTCTACTCAACACTCCTCTTCAGACTCATAGGGAATAGATTTGTCATGAAGTTTAAACCAAACCTTTTAATTTTTTGATTCTGCTTAAGAAATATTAGTGTCCACTTCAACAACTTGGTATCGTGTGTCATTGGTGTACGTTTCACGTGCCTCACTACAATGGGCCTTGACACTTTTGGGCTGCAATACTTATCTATATTAATATTGTGGGTAAATATATCAGTAACCAAACTTCAATACCAAATCCAGCTAAAACACTAGGCTGAATTTGATAAATTCATTGAGTAACCAACATTCAATTATCAATGTAATCAATAATTTTCGTAACCATTTTGAGATAAATTAATCATAACCTGAAACAGTTAGATAGTGTATACCTCTAACCAAAATCGAAAACTAAATTATAACCGAATCAATTAATTTAGGAAGTAGTTCATGAATGTAGACAAATTGGGAAACTGAATACTTGGACTGAACAAAATACTTTTTATTCTCCAAAGATTTCCTTTATGCACATGCATACAGACTATACACATGCATACACACTATACACATGCATACATCATAATATGTCTCTCAGGACAGATTACAAATGTAATGAACTAGTATGTCTATATGTTTGGCGATCAATGTTCCAGTCAAACAATATAAGCAAATTTTGTGCTTTGTAGTTTCTTGTATAAGCAATAGCATGAACATCTTTTATACTGCACTTTGAATATGAGAAAAGTTGTCATTTATTATCTGATGGTTCTTAGTGTGATGCCAGCTGCCTTCCCACTTCTATGGCATTCATCATGGTAGAATGCCTTTGCCCCTTGAAATGGAAGAGGAGCCTGTTTATGTAAATGCCAAGCAGTACCATGGCATTTTGAGACGAAGACAGTCACGTGCTAAAACTGAGCTTGAAAAGAAACTTATAAAAGCTCGAAAGGTAAACTAATTTTAAATTTGTTCGTTCTGTGGACTCTAGTAGCATTTCCTAGAATTAGACATGAACACATTTGTGCGCACATATACATATACACAAGACACAAGACACAAGACACAAGACAATCTTAAAACTTAATGCTATTTGATTTTCTCTAGTTGAAAGTGAATCTAACATATGTTTGAGACATTATGATGGTCATGTTGAGTTAGGTACAAGCAACTAGCAAGGATGAATTATTGATATCAATGCATATGTTGGTGGTCTAGACTTATTGGAATTTTTGTTCACTATGTTTATAGGTAAAAATGGATCGAAATGTAGGTAATTCACCTCAAAACATTCCAAAATATAGAATTTCTGAATGTAACCAATTTAAAATGCTTCACAATTTCCCAAAATAAAGTTTTCTTGCCAAATATATGACATGTATCATTTGTTTGACAAAGTTTTACTATCTTGGAAGTTAGAAATATGGAAATATCTATGGAAACGCTAATGTTGGTAAATGTTGCTAAGTAGCAGTCTTTTAAGACTCTTCACCAATGTAGATTTATCCATGTATATGGGTAATATCAGAGAAATATTGGCAATATCCTGGAAGAAAATGTCTTTCGTTCATATCCAGAAAAAGAGATGAACTAAACATAGCTAGGGAATATGCTTGAATGGTGGGGAAAATAAAACAGACTTGCTAAGTTCTGGACGGGGATGATGAGAAATATACCCTTAGAGGAATGGCTGAAACCAATTATGAGGAATAAAGAATAAAATGACCCATGCACGCAAGCAGATACATACTTTATACATATGGACATGACTATGTACCTTTTCAAGGGGTGTTAAGCCTTTTGTTTTCAGCTGAGTGAATGATAGGACACACATGCGCATGCACATGCGCGCGCGCGCACACACTATATTGTGTATATGAATGATTAAATGACCATATAACTTTTCAAGGAGTGCTAACACACACACGCACGCGTATGTATGGCCATTTAACTTTTCGAGGTGTTTTAAGAACACTCACATTCTAGGGGTGTTTAACACACACACATAACTTTTCAAGGAGTGTTAACACACACATACAAACACACATGTATGTATGGCGCTTTAACTTTCGAGGGGTTTTAAGAAACATTCACATTCAAGGGGTGTTAAACACACACAAATACACACACTTTACGTATATGAATGACCATGTAACTTTCCAAGAGGTGTTAAGCTGTATTCAGGTAACTGAATGATTGTCTGTAAACAGCGTAAGGATTTGTTTGATATATGATTTTGTATTCATTCTACAGCCATATCTTCACGAGTCTCGGCACCTACATGCATTGAGGAGGGCAAGGGGTTGTGGAGGTCGTTTCCTTAATACAAAGAAACAAGATGATAATGACGAAAATTCCTCACCAGAAAAAGGCCTGAGTTTGGATGCAAATGATTCAGCCCAATCTGCCAAATCTAGTTTTCAGTGCTTTCCCAATACAAGCAATGGGAGTCTTGATTCCTCAAATGTCCAGCAAGAAGGATCAGATCCATGGTTCAGGGCACACAAGAACCACACACTCTCTAGTGATAATGGCAATGGCAATGGCCGTGGCCCATCATCAGCATACCCTTCCACGTTTGGTGACAGCAAGGAAAGTGTTTTCCTGGGTCAGCAGAGGGAAAACATGCAGTTGAATGGGTCGTCTCGTGGGGCCATCCCCAGTAAATGA

>PpNF-YA2

ATGCGTACGTATTTATGTATTTTATTTATTTATTTGGTTTTATTTTTAATTTAATTTTTGTTTTTCTGGGTGTAAATGAATTTTCTTTTTGTATGAAAATGAAAATTCAGAGTATTTATTTATTTTTTGAGGAGCAGAAGACGACAAAAAGCGAACTGCGAAGGAAAGAGACGAAGTACCAAACGAGAAAAATTTATCCAAAGAAAATGTTGGCAATAAATAATCACAAGTTAAATTAAATGACACGTAGAACAATATTAGTTTATATGAGAGGGAGAGAGAGAGAGATGGGAAGGTGGTGAAAGGAGGGAGGTGAGGTGGTGGGGTGAGGAGATTGAATTGTGACTTTGTGAGTGCGTTTAATGCAAGGAAATCTGTCGTTTAAGGAAATGAAAGATGGAGCCCTTGCACCAAATAAGCCAAACCAAATGAGAGTAAGTTTTTGAGAGAGAGAGAGAGAGAGAGAGAGAGAGAGAGAGAGTCTGCTTTGTGGGTTTAAGGAAGAAGAAGACATGCACAATTCAGAGCTTACAACAAAACCCATTTCTTAATTCCAATCACTTTACCATATTATCCCAAAGGTGTCCCTTCTCTCACTACTCACTCACTCTTTTTGCTTAAATTTCATGCCTTTTTTCTTGACCTGGGTTTGTTTTGTTTTTCTTTCTTTCCTTTTAGTTTGTCTGAACTTCACTGGTAAATATATATTTTTTCTTTAAAAAAAGTGAATTCTGTTATTCAAATTTTTCTGCTTGGTAGCGAGCCTGTGGTTTGTTTGCATGTGGAGCTTAAGATGGTGAGGTTGGTTTTCTACTGTTTTTTTTGGTTTGAGATGATGGGTTTCTTATTTTGGGTATGACTTTTGTTTTGTTTTTGGTTTTGATAATTATTTTGGGTAAGACTTCAGAGTCATATGTTTTCCTGATTTGGGAGGAGTTATTGTTGTTGGGCGCAATTGTCATTATTTTGTCCTTGTTTGGGATTTTAATTCTTGGTATTGCATGTTTAAAATGGTTTCCAATTTGAGAATGGGCTTTCTGATAAAGTATGGAGTTTTGTTGTGTGAGAGTTTTTCAACATTTTCAGCCCTGCCTTTTCCTTCTTTTGTGTTTTTTTCATTTCGTAATTGAAGTATATGTTTAAAAAAAAAAAAAAACTAAATTAATATTCATTTGCTTTACACATTCTGATATTTAACAAAATTCATAGTTGATTACCTAATTTGGTATTTTTGTTGTGGAGCATAATATATGCTTGTCTTTTCCCCCTTGATATAGAAAACAGTAGCGCTGTGTATATCTAGTGATTTGATTGGAGCTGTCTTCCATGCCTTTTAAGTACTTCAATGTTTTGTTGATATTAGGATTGAGGGAAAGAAGGGTGTTTGCATGTGTTTTGGGTTTCTGCATTTTATCAATATCCAAATTAGTACTATATATGGTTTAGAAATCATCACTACACCCAGTGACCGAGATTATAGCTTGGTACTTGTATCGAATTCTAACAGATGGAAGAGATGGAGGTCTGCCTTGCTGATATGCAATGCGGAGGATAAGTTGATCTGAATCCTATTTGGTCCCAGATATGTTTGAATATCTTTTTATCATCCCCTAGATAGTTTTCCCTCCACCTATGGTTCTTACTGGAGTTTTATATCCACCACTTAGGATCCTGTGTATGTGGCATGCCGTATAGTATACTAGTATATCTGCTGCATCAACTCTTGTTGGTGTGCCCTTTTGGGTACAGTGGATGTGATATATCTTCTGTTATGCATTATGTTTACTATCAGTACCGTAGTTCACTCAAGCAATGTTATATTGTTCTTTCCTCTTAAATTGATTTATTAATTTCGGTATTGCAATTTGCAGTGCATATGAAAAGTTAATTTACCAACGTTCTTTATGACTTGAATGTAGTAGCATTGTCGCAGTTTGCATTCCTGGTTGCTGATGAGTGGACAATTGAAGTAAAAGTTCAGGTTTTTCTCTTTTAACATATTTGAAGATCTATAAATTTTCAACGCCACAATTTGTAGACAACCTGAATCATTGTTTTAGACAATGCAGAATTTGTATAAGCAAAACTCTGATATTGGTTCTGCACATTCGACATTTCCATTCATTGTTGGATCATCATCATGGGAGTATTCTACTGATACACATGTCCAACAGTCTACCTCCAAAAGTTTAATCTTCAGGATGGGCGCACCTCTACAAAATTGTCATAACAGAAAGCAATCTGGAATTCACTTTCAAGATCAAGATTCGTCGTCAACTCAATCAACTGGTCAATCTCATTCTGAAGTGGATAGTATGAAAGAAGGCAACCCATGTGGGGAAGGCATAGTTTCAGCACAATCAGGTTCGTTAATCTCTATGTTTTCGTTCCCTTATTTTTCCTTTTACCTCCCTTCAAACCTATGCTTGTGGAAACTGGCTTCTCATTGATGCTTCTACGGATTTTTTGTTCCTTATATGTAGATATGTTCTGTATATATGGGGTTATTAGTGCTTTTTTTGTTTGTTTGACAAGAAACAGAATAATTGTGAAAGAGATAATCGATTCTACATTTATGTACCACTTGGAGTAATGTTCAGCAACAATGATCTGGAGATTAATGCTCCTTATTCCATTTGCCAAAACTTGTATCAATGTGGTCTGAGTTGGCTTACCACTGTACTTATCAGAGAACTTCCCCCAATAAAGTAATGGGATCTTCACATTGATTTCCTTTGTTACGACGCATGAACAATATCTAATTTGGTTAGTCTTGGTCATGCAGGATATAATGAAAGACAAGGGAAGCCTGTTGGTGGTCACCTGAAATCACTCTCGTCAATGGCAAGTCAGGGCTTTGTCTTCCCGTCACAACTTGATTTTAGCCACCCAATGGTAAGTTGAGATCTGCTTGTGTTTTGTCTTGCCGAAAGCTGGGAATCTTATACTTTTTCTGATTACATTCAGGGTCACATTCCATTCCACTATGCTGAACCATACTTTGGTAGCTTACTTGCTGCTGCTTGTGGGCCACAAGCTACGGTGAGTGCTTGAAAGTGAATACTAATAGAACCTCTTTTCTAATTTGTGTTAGCTTGCTTTCAATAGTATGTTGGGAACCTAAAAATTATGCTTTTCAATATTTTCCACATCAAATTATATCAATAGAGTTCAAATTATATTAGAACTTGAAAATTCTGAGTAGTAAAATGAGGCTTTGTCATTCCCTTCAATGTGATTGCGCAAAATCTTGAACAATATATGGTGCCTTTGGCATTAAACTATGTTTCTATGTTGTTAATAGAAGTAATCATGAAGGGCATGCCTTCACCCAGAGAGCACATAATAGATTCTTTCAATTTGCAGGATTTTCAAAACATGAAAATCAATCTTGGAGGAAGTTTTAAATGGCTGTTTTTTCCAATTATTCTGCAGATGCTACAACATTAGGTCCAGCTTTGAGGATGCATTAATTAAATTTTCTTCATTATGACAATTGCAACCTCAATACTGTCAAAATGAGCATAAAAGGAAATTTTGGAATCTTGTGTAGGACAGTGTTCATGCATGTTGCACTTCAAACTGTTCATTCTCTTTTTACAGTGCCAAGGCTATATGCCTGTCTTCATTATCAACTCATCATAAGAATTTTCAAGGTTTAGACCCTAAATATTTTTAGCCATGTAGCACCTTCCTAGGCATCTTTTCTTGCAAATTGTTTAAAGTCAATGTACAACAGAGAGTTTGATATTCTTAAAGTAGGTTTGCTAATGTGTTTTCTTGCTATCTTATAAATTTTGATTCTATTTTGCTTTGCATCTCCTTTGCTCTTATTCTTTTCTGTTCTCCTTTATTAATAATTATTTATAGTAATAAAGTTTCTTCTTGCGTATTTATTAACCCTAGACCAGTTCTTTATGAAGTTCAACCATCAAGGCATAGATGCAAAATGAGTGAGGAATCTTGAAGACCTATTATTGTTGGGATCAAAATACTAAAGTTTTATAAGATATTATATTATATCTCTTCTGTGGTTCTACCTTGTGGATTCTACAATTTAGTGGTTTTGATATAGATTCTGATTCTTTTGCCCTTTAATCGAACCATGACCTGAAGATTTATAAAAATAGACCACATCTGGTGGTTTTTGCATAAGAATGTGCCTCTTCTGTTTCCATTTTATGAGATATTCATCATTTAAATGTCTCCAGATTCATCATCCCCAGGTGATGGGGATTACTCCTGCTAGGGTTCCCTTGCCTCTTGATCTTACAGAAGATGAACCCATTTATGTAAATGCAAAACAGTATCGTGCAATCCTAAGGAGGAGACAATATCGTGCCAAGCTTGAAGCTCAGAACAAACTCGTCAAAATTCGTAAGGTATGTGAAGTGTTTTTTTATTGATTTGTAAATGTCTCTGTGCAGGGCTTTATGTTAAGGAGACTTGTACTGTTTTGACTGACAAAAGCATTTTCTCTAAGAAAGGCCATTCTGAAATATGTGCAGCCCTATCTTCACGAATCTCGGCATGTTCATGCATTAAAGAGGGCTAGAGGATCTGGTGGCCGCTTTCTCAACATGAAGAAGGTCCAAGATTCCAAGCCTAATACAACAAACAGAGTGGATGTTTCAGGCTCAGCTCAGCTACATTTGACCAGAAATATGTCCGAATCCGAAGTTCTTGAGCCTGACAACTATAGAGATGGTGCTTCCACTACTTCTTGCTCTGAAGTCACAAGCACTTCCAACAGTGATAATATCTTTCCACGGCAAGATTTCAGGTTCTCTGGCTACCCCTCTCACATTGGTGGGACCATGCAAGTTCCCTTTGTTGATGTGCGTGGTGGTGGGAACCAGCACCATATTTCCCTCCTCCGGTGA

>PpNF-YA3

ATGACTTCTTCTGTGCATGATCGTTCTGGTAATTTTCTTTCTTTCCAAAATACTTTAAATATTCAGTTGGTTTCAGAGTTGCTGTAAATGGATAAGACTTATGTTTCTGGCATATAACTGAGCAGGGATAGTTAATGTGACAAGAAACTGAATTGCTTTTTGTTTGGTGCTTTTGCGTCTTTGATATAATAATGGTGTAGATTTTATATTAAACTAGTTTAAGTAAGGGTTCTTTCCGTTTCTGTTGGTGGATTGTTCTACAAACCAATAACTTTTGCGGGGAGATTAACCTATGTTACCTTTGTAATACCTAAGACCGAGCATGGGCTTAGGTTGAAGTCTTGATTTGGTTTTAGCAAGATCCGTGTCAGATCCAACCCTTGACTTCCTTGGTCGTTCCTGATTTTGATTTATGGTTTACATAATTTACCATCTCACTAATTGCCCATTTTCCTTTTCATTTGACGTGGATCCTCAGGAAGCCTCTACCTTGTGAAAAAAAATAATTTCCTTGGTTGTTTTAGTCGGCTTTAAGGCTGTCTGGTAGTGCTGTTTTATTATGTTCTGGTTAAATCCTTCTGATATTGCTCCTATGCTACATATGATCAATTGCGGTGATGTAGGATTTACTACACTTTTCCCTCATATTTATGGTTTTATAGCACTGAACTTCAAAGCTTTAATTCTTTGTTATTTTATTCATTTATTCATTTTGGCTTTTGTTTTCCTTTTGTGAAGTGGCATCATGGTTTTTGTTTTTTCCTTTCTTCACTTGAGTAGTACAACCATTTCTTGAGCGCATTCTTATCTACTTTGAAATTTTGCTTTTGCAGATAATAGTGAATCTGATGACCAGCAAAGTCATTCTTCCTTGCCTGCAATTGGTATTTCTCATCCTGGAATTCCAACCCCAAATATTCAGTATGCAGCCCCTCCACAAGTTGGAACAGGACATGCAGTGGTAACATCTTCAACCTTAAACTTATCTAGAGTTGACGTTTTCTTTTCTTGCAAATTTCTCCTTTAAAAAAAGAAGAGGAAGTTTCCACTTTATTATTGTGAATTCCACTATTGTGTCATCAAGTTTGTGACTTCTTAGGTGCCATATATATACATATTTATATCATGTGTATATGTAGTTGCTGACGTGTATATGTTTGTGATCTCATATATCATGTTTGTATGTCAATATTTAGAATGTGTTGAAGCTTATATGTGGTACCATTTACTCAGTTCATCAAGTAATTTACAATTATTGTGATTTTAATGCTTTCTGAAGTAGGCACTTTATTAAAGATGAGATGCTTGTTTTGTTAACTTGGGTAACTGTGTAGTGAAGAAATGTGCAATTGCATGCTCCCAAAGAAATTGTGCGTATGGTTGCAAGGAAAGTAGATTCAACCTTCAAGACATGCATCCAAAACTGAGTAAATAAAAAACATTAGATGAATTAGGGACTAACACTTGTAAAGAAATTGGTGGGATCAGAGGACTAAATTACCGATATCATCCTGTTATGATCCCTTATCAGCATAATGGGTAATAATTATGAACATTCAGTGCAAAATTGGAACTGAAAATATATGCATGTGCTATGTGAACGGTAAGTTGTATGGGGGATACTTAGATGAAACTATAACTGGTGTAGGTGCATCTAGTGTTACATAGCATTGGTGGTAGTAATTATGGTGGCTAATATCAGAAGTTTCCCTAGGTGGATGATTAAGTAATTAAGAGTTAGTAGTGGTGGAAGGCATCACTTTTGTTAATGTTGAGTTGTGTTTCCTGCATTTTGAGAGTAACAGGAACTGCTGCTGGTGATGAAGCTGAATAAGTTGGTTGTGATGGTTCTGTTTCTCATGGTTTGTGGCTAGAGACAATGTATTTCCTTATAGCAGGTGTTCTTAGTGATGGCTACAAGAGATCCTTAATACAGGTCTAACTAGTGGTGGTGGTAGCAGCACTATTTAGGCCTGTATCAGAAAAGGGCATTCATGTGTGGCTTTTTTTGTTTGGGTGGGTGGGTTCGGGGAGGGAGACGATACCACCATCTAAAGATTGTTATTTATGTAATGAGGAGGTTTACAGGCCTAAATAAGGAGGAAATAACAAACCCCCATACAAGGGGAACTAAGGAGGTTTACCCCCATATAAAGAGTGTTATTTATTTAACGAACCCACAATTAACAAACCTTGATTGCATTAGGAAATAAGGGGTCTAAAGATTGTAAGATGATTTCTGATAACTAGACAATATTACAGAGTTTTTCAAGCTATACCTAAAATAAAAGATCCATGTGTGTTTTAAACTCTTCTTAATTTGAAGTTTTAAGATGCATTTTCTTTGGGAGACAACTATGTGATGTACTTTCCAAGGTATTTAGACAGCTTATATGTGGTGGGCTCTCCTAAATCTTATCTTCTATACATAGAGAGATTTTAAACTATGTTTTGTGGGGATGGAGATGGATGGGAGGAATAGTTTCAAATTGACCCATGAATATCCTTAGGTAAATGTAGTTTGGGTTCTAATCTCTAGTAGAAGTGATAGTTTCACTTTTGTCCCCAAGCAATTAAATTTGAATACGGGGTATAATTGTTAAAGTCTATGTTTACACTTCTCTTCGTCCTTTCCATCCCTTTTACCAAACAAGAGGAAGTAAAATTCTCCTTCTCTTCCTTTATCCTTCCTTTATGAATTACCATCTGCTTGCTCTTGCAACCTTCCTTCCAAACATAGTATCAGTGAAATTGTTGGTATTGTTTAAAAAAACATTATAGAATAAGAATGTTTAGTATCATTGGAAGTTTCTGTTTCTCAAGCTAATTTTCATGTAGTTGTTTTGCAATTTTTAATGTAAATGCTTTAAAAAGGCACCTAATTTAAGTTTAGAATTTTGATACATTATCATTCGTTTGTCTAATTGAAGGCACCAGCAGCTTACCCTTATCCAGACCCTTACTACAGAAGCATCTTTGCTCCCTATGACACACAACCATATCCTCCACAGCCCTATGGGGGGCAACCAACGGTATTTCTTCCGCCTTGTCTTAGATATAATTTCTTGATAATGGTACTTCTAATATTTTGAGCGTTGAGACCACATATTGCTTATGTGCATGAAATTTGTGGTTATTACTGTGTCCTCATTTCAGGTCCATCTTCAGTTAATGGGAATCCAGCAAGCAGGAGTTCCATTGCCATCAGATGCAGTTGAGGAACCGGTGTTTGTAAACGCAAAACAATATCATGGCATCTTGCGACGTCGGCAGTCTCGTGCTAAAGCAGAATCAGAAAATAAAGCTCTTAAGTCTCGAAAGGTAATATTCTGGACTGCAAATCCTTGCATCTCATGTTCAATGAGATGCTGCTATAGGTTTCATTTATTCATTGTCACTTCAAACTACTCTTGCAGCCATACTTGCATGAATCTCGACACCAGCATGCACTGAGAAGAGCTAGAGGATGTGGGGGTCGATTTCTTAATGCAAAGAAAAATGACAACCAGCAGGATGAAATGTCATCAGGCGACAAATCCCAGCCAAATATCAATTTAAACTCTGATAAAAGTGAGCTTGCTTCCTCAGATGGAACATCCTGA

>PpNF-YA4

ATGCAGTCAAAGTCTGAAACTGAAAATCGACTTCATGCTGATCATCATACCATCCCACCAAACAGTGTTTATTCAGAACCGTGGTGGCGGGGTGTTGGCTATAACCCCATCTCCCCAGCCGTTACAGGGAGGAATGCATCCAACTCATCTTCGTTAGAATGCCCAAATGGTGGTTCGGAGTCCAATGACGATCGATCATTGTCCAATGATGAGCCAAATGAAGATGATGATGATGATGCCACCAAAGAGTCACAGATTACGTCTCCAAGATCAGGTGACAATTCCTACAATATCTGGTGATTTGGTATAAATTTCACCTCTTTGTTGGTTTCACGGTTTAATATTTTATTTCAACTTCAGCTGGCAATGATGGGCAAGAGCGCCAAAATATGCCGCATGTTGGATCTACTGTACCTACAGTTCGTGATGATTGCCTTGCGCAACCGCCACAGCTTGAGCTTGTTGGTCACTCTATTGTAAGTTCATTTTACTTTGTTCCAAGATTTATTGCCCCCTGCATGCTGCAACTCCGTTGATAGTTCGTTAAATATTGAACAAGGTTCTGTAATTTTTAATTTAAAAGTGGGCACTCATACCTGCATGATTTTGGAATGTAAGATTCCTACCTTTCCCGCATTTGTTTTGTAGGCTTGTGCTTCAAATCCTTATCAGGATCCTTATTATGCGGGAATGATGGCGGCTTATGGACATCAGCCTTTGGTATGTTAAACAGTAATATCCTTGAGTGTAATAGAATTCTACATGCAATGTTGGATTTGTATGATATTATCTTTTGCGCAGTGATGCAATTTTAGAGTTTATGCAGATATCATACTTCCCTTATCCACTCTCTAAACAATAATTATATTTCTATTTTATATCTCTAAACAACTTCAAGGGTGGTTGTTGGTAGGAGTATTTAAATTCCCTTTATTGTTTCTTATCAAAACAAAAATATCAACGATGTTATATCATATTTGTGATGGTAGTGAAAGGGGGATGGGTAGTTGAGGTTTATCGGGACTTGTAACTTAGGTCACCTATGATCCTATGCAGTTAACATTTTGGATGGGGAAATTATTTGGTCTGATTGGGTAAATGAGGAAAGGATAATTTTTCCTTTGAAGTTGCTGTTTTCTGTTATTAGTAGCTGAAATCTAAGTTTTGGTACGGTCTTCTCATAATGTAATGGTCTTTCGGGGTTTGAATTTGAAGCTTTAAGTTTAAAATATTACTGATAGGATACTAGTGGTTTTTCTTAATTTCTCTTTGTAGCTAGTATTTACTTATTACTATTTCTACGGTTTGGTATTCCATTATCCTTATTAACCAAATCCCACATTGTCTATATGTTGCATGTTTTTACATCTTACTTTATAGGAAAATGATAATGATGATAATATGGTGGGAGTGAAGCACTAGCAACAACTTGCCAAGATATCGATCTTCTTTGAAAGCCCAAAGCCTGTTGGCAGAAATCCAATTTTAAAACCTTTTTATCTAAAGAGAAATATCCATCATACAAGAGTTGAATTGGTAAAACTATAAAATTACTAGGTTTAAAGGTCAATTGAAGAGTTAGTGTCGACTGTTATTAAGAATAGGTTTAGTTTGGGTCTTGGAAATACTGAATGTCTAAGTTTGCTTACGGTCTCCTACTATTTCTTGAGTTTCTAGTATCTATTACAAGTCTGGGTATTTCTTGGAGTCTATGAGAGTAATGTTTGAAGACTACAATTCTAAAGCAAATCAGATTTTGGATAAAAGAAATGAAGTGTTGTTGAAAGGCTTGGTGCCTTGATATCTCCATCTGCACTTTCCCTTTTATTTCTGTATCTCTCTAATTTGTCTAAGATTACAGCAGGCCTAAATCTTTTTTTTTTCCTGAACTTTTTTGACGACTGAAATAAAGCCTGCCACAGTCCTGGGCAAAAGATCACCTTGTGTAGTCATACATCAAAAGATCACCTTATGTTGTCGTACATCACGGTGGTGCTGGTGATAAAAAAAGATTAGTTATAATTTTCAGCCCTTTCAAGTTGTGTTTCTTTGGTATGATCTGAACTTATTCTGGGGTGGATTTGGAAGGTGAACAGTAGCAAATATCTTGATATGTACGTAATGCTGAACATCTGTTTCAATTGGTTAGGGCTATCCTCCTTTTCTTGGAATGCCACATGCTAGAATGCCATTGCCTCTTGAGATGGCACAGGAGCCTGTGTATGTGAATGCAAAACAATACCAAGGGATTCTAAGGCGAAGACAGGCACGTGCAAAAGCAGAACTTGAAAGGAAGCTAATTAAAGTCAGGAAGGTAGAAATTTAACTTGCAGACACAAAGCCTTTAACGCTATAATATACATGTTTTATTTCATATATGTGATGTGAATATGATATATGTATGAGTTGTTGTGCTCACACGCCTATATTTCTACTTCTAATCCCACCTCTTCAGACTGTGTACATCAATTTTAATAAGACATTCATAGAGTCTGTTCTCTCTGGCTAATAGGAAGTATTGACTTTGACATAATCACAATTTAATTGAAGACTTCTGATCTTGTGCAATGAGCTTTGAAAATTGTCGGCTTACACACTGCGACGTGCTTAAGAAGTGGGTTCCAATATTGTGATGTGCTCACTTGTGAGCATATCATGGAAACTTACAAAGTTGGTGTACATATGAGTTTTTGGGCTTTGATATACCCTCCATATCAGGAACAACTTATTTGACTTGCGCACTTTCTAGTCTCTATTGCTTCCATGAACTTATTTGACTTTCTACTCTCTCTCTTACCAATCAAAGATTTGCATGCACAAATTTTATCCCTTAGTGTTGATTATAATTGATTTTATAATCACGTAACATGGATAAATATACATTTTAGTGTTGATTACAATTGAGTTTACTATGAAGTAGTTCAACGATTGTGTTATTGATATTTGGTAACATTTTAGTAACCGTGTTTGTCTAATCGGCTTCTTGTGAGATTACTGTTTACAATAGCCATAGGATGTTTGTTGTAAGAGGCAGAGGATAAACAGTTGTAGATTTCGTTTTTTTGGGGAGATGAGTTGTATATAATATATATATATGTGTGTGTGTGTGTGTGTGTGTGTGTGTGTGTGTGTGTGTGTGTGTGTGTGGGTGTGGGTGTGATGATACTTCAGTTAACGAATTCCAACTCAAATTGTTAGTGTTGGTATTTATATTAAGTATGAATGTACCTGTGAGCCTAGCTGACTTTGGTAACTTATTAATTCTCATTTTCTATAGCCATATCTTCATGAATCACGACATCAACATGCTATGAGAAGGGCGAGAGGTACTGGAGGACGTTTTGCAAAGAAAACTAATGGTGATAAATCAAACAGCACTGGCCAAGAAAAGGGCACAGGCTCTGGTCCAGCCCACTCGTCACAATCTGGCAGTTCATCAGGTTCAGAACCCTTTCCCTCAGATTCTGCCGAAACATGGAATTCCTCCAATAGTCAGCAAGAAGGAAGAGGATCCCAAGTGCATGAAGCGTACCCAGGTCACAATTATGCAAATGGCAGTGGCTGCTTCCAGACTCATGGTGGCTTGCAGGCTTCGATGTATCCTTCATACTCGGGCAAGAGAGGGGAAGAAGGAGATTGTACAGGCCAGCAACGGGGAAGTATTTCTTCGAATCAGGCCTCACAGAGGCGTCTTGCCATCCAGTGA

>PpNF-YA5

ATGGCTATGCAGACTGTCTATTTCAAGGAACATGAAGGGATTACTAATCCCATGGGGCAGCTGTCATCAGCATTGTCAGGACCTTGGTGGAGTGCCTTCGGATCTCAACCGTCTTATGGAGAATCCTGTGGCCAGTCTAAACCTTTTACCATGGAACACCCCAGTAGTGGGGACCAACTCACTGCCACTAAACTTACAGGGCGAGGTACTGAACAAGGACTCGATAAAGGGAACACAAATCAGTTCACTATCTTTCCTGGTAATCTCCTCCTTTACTTTTGATGTATCTCTTTTTCCAAAAGATTTTTCTTTATTTAGTTGTTTGGTTAATAGCATTTACTTAGCGACTTTAAAAGGGAAAAGAAAAGATTTTCTGGCTTCCTTATCTTCATTTCAGTTTATGCCTTTGACATAAGGTTTGCATCCTTCGTCACGAAGGTATCTCTTTGTTTCCTTTTTCACCTTATACTGATCGGAAATAAGCTGGAATGTGGGGTCAGGAAAAGTTTTCGTGTAAAAAAGTTGGAGGATATAGCCCCTTTTCTGATAATTGAAGGCGATGAAGGATTAACCTTCTGTAGCCAAATTGCATGTGGAAGTAAAGGTTTAGTCATATAGTGTGAAGAGAGTTAGTGTACTTAATACGTAATAGATAGTATAGCTGGTACAATAATCTTTCTATGACATCCTCAAGGTGAATTTTGCTTCATGTGAGTTTGTTGATAAGTGGCTTAAACATCATAGATGATAACAGTACTGCTGCAAAAAAGGAACAAACTATAACTAGTTAAAAAGATTTGTGGAAAACCAGAACTGAGATAATAGGAATGGTTCTCCTTTTAGGACCACATTACAGTACTTATGCTATGACGATCAGAGCATTAGCCCTAAAGGTTTTCATCTGCTGGTGAAAATGGTTTTTCCAGTACCCTTTTTATTTGCAAATTCCTATTAGTTCTTGTTCAAGATAAGAAAGAATCTTTACGTGGTATAGTTGCAGTAATTGAAAAAACGTTTCTGTTTAGTTTAACTAGTGGACCTTGGGATGGAAAATAAGCAGTGGCCAACTGGAAATACAGTGTAACTGACTTATTGTGAAGTAAGAACAACAAAATGGGACTTAGTTGTTAAAGTAGATATAGACAAACATCACAAAAGGAACATCTTTTTCATGAAATGAATAACCGTCTTCTGTTTGAAGTTTGACTAAGCATTGACAGATGGCACAGTTGTGTGGTCATAGCACCCACATCTACTTGGTATCCAAAATTCTTCGTAACGTAAATCAAACATATTGCACATGTTATTTAGAAAGTTGGTTCTTGAAAATTTAATTTGTCATATCAAATGGTTTGAATGGTGCCACTGTATACCCTGAGTACCCATCTCTGTCCTGTAAATGTTGAAGTTGTTTTCTTCATCATGCATGGGAAATGTAAAGAAGGGCTTCTCTTCCCCAAAAGATCTGGTCTGGTTTGTTGAACTATCTAGTCCAGTGCTAATTTACAGAGGATGTAACGAATTGGACCTTCCGTTGGAATGTAATTACCAATTCTCAAAAATGCTAGGGAAAGGGTATGAAAATGATGAAATTGGTTGTACAGGGAAACAATGCGTACAAAATAAATGATAGCAAGAACCATGCTCTTTTTAAAATAGAAGTTATTAGATTGTAATCATTATTTAGTAATCAGGGAGTGGAAAATTTGAAGTACATGACACCTTTAAGGGCACCCAGGGGAGGAGAGGACTTTAGCGTTGATGACTGGTGAGGCTTATGAGCAGCCTATTTAAAGTTTGCTTGTGTTACTTTGTTTCACACGCTTCTGTTCCAAAACGCCCCCAAAAGCTGCCTTGTAGGGATTAGGTCCAAGGTAACAGCATTACTTGGGAGTATCCAACATTGGACTTGAATCAATTAGTACAAAGGTTGGGTGTTAGTTTTAGAAGCATTCTGGGGGTCATTTCCAGTACTATTGACCACAAATATAGAAAAATATCTGTAGAAGTGTGGGAAGTTAATTTGCAGTACCTCTACTGGGGTAGAAAATAAACTCTTGAAGATTCACCTGCTGAGTAGACATCCCAGTGTGTAGACATTACTTGATAAATAAATGATAATACTATTCTCTGACAATAGTATTAGAACATACTAGAATGCCTTTACTGAAAACTTTCTATTCTCCCTCACAAGCCCTTGTTAAGTAGAGGGGAAAACAAAACCTTACTGATGGATGAAATATCATCTAGTTCACATATCTTTGTTTTACAGCTTGTTCAAAATGATTTCCTTCTCCAATGAAACCCAGGTTTTCATTCTTTGATTGCACTTTTGGTTGTAGACTTAATTTGATTATCATCTTGACAGAAATATGTTTAACAAAATTTTGATACCCAAAATATGAAAATATGCACATAGAGGTTTTAATTTCTATCAAAGCTCTTTTTTGTTTTTGGAAAGGTTTTGATACCCAAAAGCTCTTTATACCCTGGGAACTTCTGTTCAATCATGGCTAACCAGCAAAGTAGTTTCTCAGTCGGGTTTAATTATTAATTTGCAGGGGATGGTCAAAAATCCCAGGCAGCAATCTCCCTGCAATCATCTCCGCAAAAATATCGTGCTCATTTTGAGCTGGGATTCAGCCAGCCTATGGTAAGCCTTTCTTTTCTTTTTTGTATTTTTTGAATTAATAACTTTTTAGGACTAGTGAATATGGTTTTGGTGACTCTTGCTTATTACTTGGTTCTTTGTTCTGGACTTGAACTGCAGATCTGTGCAAAATATCCATATATGGATCAATGTTATGGACTCTTCTCAACATATGGACCTCAAATTTCGGTGAGTCTTTTATCATTTCGCCTACCTCTTTGTATGTGGATCTACGCTATTGCAATTATGTTATTCTTGCAAAGAGATTTTCTTTTTCTTTTTTTTCTTTAACTTTAGAATATTCTATCTTTTACATAGAACTTGTTTTAGTACGAGTTTCATTCTATTAATTTCGTAGTGAAACAAGCTCATCTTTGTCTCTCCTGATACTATAGTTATGAGAACTAGAAACCATTTCTAGGATTAACTTTGAAAATCTGTCTTTGTTCCCCTGTCTGTAAACGAGCATGAGAATTAGCACGTCTCTGCTTGTCTTTGATATTTTACTCTGATTAAAAATTAAGCAATGTCAAGACAAGCAGAGAATTCATTTCTTAAGGAAAATAATAAGAAACCTTATATAGAAGCGAAAGAGAAATTCCACCTAAATATTAGCAAAGAAAAGAGCCAATTGAAGCCACATTATCAGTCAGTAACTGTTCATACATCTGAAAGTGCGGTTTCAAATGTCATCAGGGAAATATAAGGTTATGCAGGTTTTGTATATATGAGTTTTACTCTTCAAAGGATCTTTGTTGTTGCTTGGCTGTTGCTCTACATTTTTCATGATATGTTCTTAAGTTTGGCAAATTTACTAATGCACATACTCATCCATTTGCAATGGATTAGGGGCGCATTATGCTGCCGCTGAACTTGACTGCCGATGAAGGACCAATCTATGTAAATGCTAAGCAATACCATGGGATCATCCGACGCCGACAATCCCGTGCCAAGGCAGTCATGGAGAACAGAGCAGCTGCACTTCGTAAGGTATGATCTCTCAAAATTTCATTATCTGAAAGGGCGAAACATCAATATAATGTGATTGCTATAAAGGTATTCCAAAAGTTGATTACTGTCACTGGATGGAAAATCTGAATGTGTTACATGAAATATGAAGTGACAAGTTTGGGCCACCTAGATTCTTGGCTTCTCGTTGACAGATAAAACAACATAATTTAGCTACTTATGGAATTCTAGGATTGATAGTCATCCATCCACCCAATTGTTTGGCCTATTGGATATGGGTGGATGTCTTTATTCATTTGGTTTTTCAATTCAAGATCTTTTTGGTTTTCAAGGGAGTTTTAAAATTTGACCAGCTTTAATATTTTATAAAATAGGTATAAGTAAAATGACAACTTTACCAAATTTCCAATAGGATATATGATTCAAAACCAAATTGCACATCTGAGGAAAAGATTTTTCTCTTTTATTTAATGACCTATGATCTTTCTTCTTTATACAGCCATATATGCATGAGTCACGCCATCTTCATGCAATGCGTCGACCAAGGGGATGTGGTGGTCGTTTTCTGAACACAAAAACTATCAACAATGGGAAAAACAGAACTGAAGGGACGCAAGTTGGTGATGGACAGCTGTTTAGGCACTCTGGTTCTCATAGTTCTGAACTCCTGCAATCTGAAAGTGGAACCTTAAACTCCTCAAAGGAAACGAATGGCAGCAGTTCAAACATATCAGGGTCAGAGGTGACCAGCGTGTACTCTAGGAGAGATCTTGATCGTTTTTCCATCAATCATCTCCGTCCCTCTGTGCACTCTCTGTCAGACGTGATGGATAGTGTGCGTGGTATGGTCATTCCGACCAAATGGGTTGCAGCAGGAGATAACTGCTGCAACCTCAAAGTTTGA

>PpNF-YA6

ATGGCGGTGCATTTGCAACCCTTTCTAGTCAAAGGAGAGAGTAAACGTAAGCGTTGATGGAAGCAAATGGGCACAGTGGGGGGTGGAGCGTGGGATGTTTGAGCGTGGGGAACAAGTGATGAAAACAGTGAACGGCGGCTGAGATAGAGAAAAGTGAATGCAGCCAAGCGTTAAGGGTGTGTCTGGGGGCGTGCGTGTGGGCCACCACCATGGAAGAAAAAGACGACAGCTGGAAAAAATACAAGAAAAATAGAGAGAGAGAGTCAAGTCAGAGAGATGGGGTGGGTGGCAAGTGCTTTTATTAAGGAATTTATGATATTTTTTATTTTTTAATCTTTTGTGTGTGTCGAGGATTGTGTATTCTGTGTGTTGTGTTGTCACAGCAGCAAAGCCCAAGAAGAAGGAAAAAATATGGAGTGTGAGTATTTTTGAGAGTGTTTTTATTTACAGAGTTTGAAGAGAGGGGACATGTCCAAGTTCTTCCCAACAGCTATATTCTTTGCTTATTCATGCCTTACCAGATCCCAAAGGTTTCTCTTTCTCTCTCCTTCTCTTGATTATTTCTTACTTTTCTTCTTTGTCTCCTCCACTCTTCAACTTCCCTATTGCTTCATTTGCTCTTTTCCTTTTTTACTTTTATGTCTTTTTTTTTAAAATCAACTTTTATATATATATTTTTTTTTTCTGCTCTTTTTGTGGATGGTTCATTTCTAGAATTCAAGTGGGTTGCCTTCTATTTGCTATTTACTGACTGCATGTGGAGTTTGGGATCATTTTGATTTCGCTTTAGTTGTTGCTGAAGATGGGAAAGTAGTAAAGTTGTCTCTTTTATTGGTTTTCTGGTTCTATTGCTCCTTTGGGCACTTCGCTTTCTTGTGTTTTTGACACTTATTTTTTTTCTTTGTTATATTTTACATTTTTGCTCCACTTATATTATTGGATATGGTGGATAATGCCTTGTTGTGTTGGGCTTGAGTGAGTAAATGGGCAGACAGAGCAACTGATATGCTATATCTGGAAATATATATATATTTTTTCTTTTCATTTTTTGGTATGCTTGGAGTTGGTCTCGAAATCTCAAGTCTTTTACTGACTGATTGATTTGCCCGATTAATCTCTCATTGATCTGCTTCTGTTGCCATAAAAGGCTGTGATTTGATTTATTGCATACTGATGCTGCATGTAACTGGAAAGCTTTCTTCTCTGTAATGTTTACTGAGCTGAATAATCCCCCATACTTTTCATCTGGTTTTCTAAACCTAGTTTTTCCCTCACATAGTAAATGAGATATTAAATGCAGTCTGTTGAAACTGGGAAGAACCTCTTCTTGGTGTTCTCAGCATGTGGAGTCTGATTTTTCATTTAAGAAGTAGAAACAGTACCACCAAGTTGTTTGAGATTTGTTGAATGAGTGGTTAGAAGGCAATAATCTGTTGTGTATTTCAATTCATTTGTGAGTTTTTTTCTTTCTTTATTGTGTTGTTTGGGATATTCATTCGTGGTAATTGTATTAGAAGAGAATTTATTGTTGAGTAATCTTATTTTTGTGCTTTAATGCAGAATGTGATTGAATGGTTGCATACTCGCCGATTACATGTAAAAATTTTATGTGTAATTAGATAATCTGTCAGTATGCTTAATATAAGGTAGCCCTTAAGTCCTTATTAAGGATCTTATTATGGATTTTGCATAAAAATTCAGGATTCATTGATAGAATTATCTATGCCAACTCAAAACTTGGACAAGAAATTTTTCAGTCAAAGTTCTGTCCAGTCAATGCCTCTCTATACTACTAATTGCATGTCGTGGAATCCAATTGAACGAGAAATTCCACAATCTTCGTCCAAGGATGCAAGCTTTAAAGTGGAATTTACGCCACAAGTTCATCACGATGCAAAGCATTTAGGACTTCAACTGCTACCGGACCAGGGATCATCATCCACAACACAAACAATTAGCCAATCTCAAGATCGATGCAATTCATCAGAATCTGGTATAATGATCTTAGTCTAATACTCCGGTACACATTTTTTCGGGTTATCAGACTTTTGGTACATATGATACATGCTTCGTAGTCATATGCTTACCATTATAGCAATTTCTGTTCCCCTTGTTTTCTTTTACTTAGTTCTCATGTGCTTCTTGCCCTTGAACCCAGGTGAAGATGAAATATGTGGCAAGGGTGTTGAAGGCCAAATGAAGCCAGTTTACTTGCTGAATAATCTAGACCTCATGTCAAATCCTTCTCAAGTTGGTTACGGAAATTTAATGGTAGGTGGAGCTGTTATACTCAGGGATTAACTTGTTAGATGGTTGTTAGTTATGCTTCTCCCTCCTTTCAGGGTCGTGTACCGTATCCTTATGCCGATCCTTACTTCAGTGGGTTCTTGGCTGCATATGGACCACAGGCTATGGTATGTTTTGTTGAGTACATTGTTTTTCTTTGCTATTGATCTGTAGTTACAATTCTGCCCCTACTCAGTTATATCTAGAAGTAAATTCATAAAAGTTGACATCTCTAATTTGCAAAAAGGTGTTGGGGTATATTCTTTGACAGTATTGTGTCAAAGAGAATGTGGCCAATTATTTATTTTTAACGTTATCCAGATTGTACTTATTAATCTTTTTCTTCTTCTTCTTTTTGTGGTTGTTGATTGAAAATTAACTGAATTCAGCCCCAGATGATGGCACCTACGCGAGTTCCATTGCCTCTTGATCTGGCAGAAGATGGACCCATCTATGTCAATGCGAAACAGTACCATGGCATCCTCAGAAGGAGACAATCTCGTGCAAAACTGGAGGCTCAAAACAAACTTCTCAAAGCTCGAAAGGTATGTTAAGTCATCATAGATCTTTTCCTGCATTGGGTAATATGTGGAGAAATTTTTTCTTAGTTTTGGATGCATTGCAAGTATATTTCATCATGTCAAAGAAAGCGATTGCGTAATGAAATGATACTTCATCCATCAAAACCAGAGCTCTCCCTTTCTGAATTTATTCTCCTTTAGAATGAATAGAGCCTTAACCCACGTTTGTTGTGGCTTTTAAATGAATGCAGCCATATCTCCATGAGTCTCGTCATCTTCATGCCCTGAACAGGGTAAGGGGCTCTGGTGGACGTTTTCTCAGCACAAAGAGGCTCCAACAATCTGATCAGAACCCCTCCAGTAGCACCCATGATGTTCCGGACTCCATCAACTTACATCAGAAAGATACACAGGACACAGAAAGTCATCACTTGGGAAGCAGTGAATTCGTCACTGCTGTCGCAACCCACTCAGACATAGCAAGTGTTTCCCATACCAATGACATCTTTCGGCAGCAGGATCGCAGGTTCTCAGGCATCCCTTCCCACATGGGTGGAGCCATGCAATTCCGCGGGGGGCTTATGCGTGCTGGAACCCAGCATTGTGCTTCAGTTGTCCGGTGA

>PpNF-YB1

ATGGTCGACAATAATAATAATAATATTGGGGCAAGTGGTGCTGCAAATCATGATGAGGACGGCGCTATGATGAAAGAGCAAGAGCAGTTGCTGCCAATAGCCAACGTTGGGCGGATCATGAGGCAAATACTGCCACCCAACGCCAAGATCTCCAAGGAGGCCAAGGAAACCATGCAAGAGTGTGTCTCGGAGTTCATCAGCTTCGTCACCAGCGAGGCGTCGGAGAAGTGCCGCAAGGAGAGGCGAAAGACCGTGAACGGCGACGATGTCTCCTGGGCTCTCGGAGCCCTGGGTTTCGAAGACTACACCGGCCCATTGCGGAGGTTCTTACACAGGTACAGAGAGCAAGAAGGAGAGAGAATATCATCATCAGCAGCTAATAATAATAATAATAATAATGAAAATAATCAAGACAAGGATAACAACAACCCCGAAGATCAACAACAAAGGAAATTGCTCAACCATCCTCATATACAAAGACCTAATTTTTAA

>PpNF-YB2

ATGGCGGAGGCACCGACGAGCCCGGCAGGCGGGAGCCATGAAAGCGGCGGCGAGCAGAGCCCTCAGGGAGGCGGCGGCGGCGGCAGCGGCGTGCGTGAGCAGGACAGGTATCTGCCCATCGCCAACATCAGTCGCATCATGAAGAAGGCTTTGCCGCAAAACGGCAAGATCGCCAAGGACGCCAAGGACACCGTCCAGGAATGCGTCTCCGAATTCATCAGCTTCGTCACCAGCGAGTATTTTTTTTTCAGCTCCTTCTCTCTCTCTCCATTGATTTTGTTCTTGCCTTTTCGTTTTTCGCTGTTCAATCGCACCGCCTTTGTGTTCATTCTTTTGGGGGGTTTTGGGTGTTTAGGGCCAGTGACAAGTGCCAAAAGGAGAAGCGTAAGACCATCAACGGTGATGATTTGTTGTGGGCGATGGCCACGTTAGGGTTTGAGGACTATATTGAGCCCCTCCGGATTTACTTGGCTAGGTACAGGGAGGTAATTATTGTCCAACTTTTGCCCTATATTCTTTCTGTTGTAGTGTTCATGTTGATTTTAGATGTTAATTTTTGGCTGTAGCATTTATTTGAGTTCTATTGTAGATTTTAATAATGGAGGAGCCTCAATTGAATGAGGTTTTTTCCATCTTGCTTTCAATTCATGCAATGGGTTTACTCCTTATCAGTTCAATCTAACAATTCTCTTTCTTTTTGTTTGTTTTGGCCCTTCGTTTGATTTATGGGATCATTGCATTGTCATAGTTGGAGGTAACTAAACGTTCTTTAGTTCTGTTTAATATCTTTTTTTATCTTTGATTGAACTACATTTGAATTGTTTAGTCTATTCGTTCATATCTCTTCGGTGGGGTTAGAAATAACTTTCTTGCCAAACTTTGAACAGGGTGATGCAAAGGGATCTGCTAGGGGTGGAGATGGATCTGCTAAAGGGAATGCTATTGGAGCCATGCCTGGCCCAAGTTCACAGGTCTGATTGGCTTTAGAGTTGTTGTTGTTTTTTTTAAAAATTCCATTAAAATAGCTTAAACTTTGGATTATGACCTGACCCCATTCTCTTTTATTGCTGGTGGTGAATGAATACAGCAGTTTGTTCATCAGGGATCATTGAACTACGTCAATCCTCAAGTAACTCTCTCTTTCTCTCACTCTTTATATCTATGTACAAATATAATTATATAAACACACACACACACACACACACATATATATATATGTATGTATATGTATGTGTAACCCTTTGAAAAAATGCAATCCCAACCAACAAGATTGCACAATGGGCATATTTACCCTATGTTGCTTTAAGATGGCATGATTTATTTGACGTTTTAGTGCTCATGGGAAACATTATAAATAATTATTGAGCATTTTATGTATTCTTAGAATTTGGCAAAAAATTTACTAGATAAGGCACTGGTTTTATAGAAGTGCTTCCAATCCGCGTAGCCCATTTAAGCCACCAAACTGCTCTTATGCCAAATACAATATAGAATTACATTTTTTTTTTTTGTCTCTAGGTGAAGATGTAGTAATTCTAGGAAACATGAATTGAGTTAAGTTGTGTATAGCAAAACTTCAGTATATGGACAGTAAATCATTTGATTGATAGTGACTCAGAGAAATCTAATTTTCCCGCAAGAGGAAAGAAAAATAAATTAGAATGTGCAGCTGTTTTTAGAGTTCATACCTGCAACAAATCTCCTCCTTCCTATGAAAATATAATAGTAACAATTTAAAACTCTAAAGCAAGACTAGAGACTGTCCGTCACTTATGCAATGCTATATAGATGAATAAGTTTTTTAGTAACTTTATACATGAACTAATCTTTTAATTCTTGTATGCTTGGGAAGGATTTGATGCTTGCAATGCCAAACTGCCTTTTATTTTTGTCTTGTCCACAAAGGTTATTGGTAGGACTTAGATTATCCAATGAGACAATCTGAAGTGAAGAAGAGATAGCTTAGGCTTTACTATCTATAGGCTGGAGGTAGTTGTCAACATGTCAATGTAAATTCTAAGGCAGTTTGCAGCAGTTGTCTGTTTGAGTTTTGGGAAAAGATAGTTTAGATTTTCCCCGGCTATAAGCACTGGATTTCTTGAAGATGCTAATAAGATGTAGCATTTAGTCATCTCAAGCAGACTTCACATTATGGACTGATTTAAAATAGTCTTCAAATAGACACATGTCATATATAAATTTCTTAGTTCTTACTAATATTCTGTCTATTACAGTGGTAATGCCAATCCATATCAAGAACGTTTAAAGATGTTTTTTGAAGCCATTTTATGAAATGTTAATTAAAAGTGTTCAGGTAGCACTATAATATGTGTTTCTTTAGAATGCTTATTGATGCACCGCATTGCTATGTGCTTTCGAAAAATGATCCATCCATCTTTTATGATATCGTGTATTTGGTTAAAACATGATGTGACACTGATGTCTTTACATTTATTTTATTGCTTTTTATAAAGGATATGATTTCTTACTGCTTTATTTGGCTATTTTAGTTAAGAGAATGAATTCTTTTGCCTTATTTTTTTTCTTCTCAATTGTAGGAGGAATATTATGTGCCTTTGAACAATATGGGAAATGGAAATTGA

>PpNF-YB3

ATGGCCGATTCGGACAACGACTCGGGCGGGAACAACGACGGCAGCCACGCGCAGAGCAGGGAGCTGTCGGCGCGTGAACAGGACCGATTCCTGCCGATCGCTAACGTGAGCCGGATCATGAAGAAGGCGTTGCCGGCGAACGCGAAGATCTCGAAGGATGCGAAGGAGACGGTGTAGGAGTGCGTGTCGGAATTTATCAGCTTCGTCACCGGAGAGGCCTCCGACAAGTGCCAGAGGGAGAAGAGGAAGACGATCAACAGCGACGATTTGCTCTGGGCGATGACGACGCTAAGGTTCGAGGAGTACATGGAGCCGCTCAAGGTTTATCTGCATAAGTATCGGGAGCTGGAGGGCGAGAAGACCATCATGGGCGGCAGAGACAAGGATAGTGCAGGCGGAGGCGGTGGGGGACTTCCGGTGGAGGTGGCGGTGACGGTGTAA

>PpNF-YB4

ATGAGGGTGGCAGGGATGAATCAATTCAGCATGCAGCAGGGGGGAAAGAGTACTAGCTCCTCTACCACCAATATCAATATCAATAATGTTGTTAGCAACAACAACGTCGTTAACCACCCCACCAGTAGTAATGGTACTACTACTACTACTGATGATAACGAATGCGTGGTGAGAGAGCAAGACCGCTTCATGCCAATAGCCAATGTGATCAGAATCATGCGCAAGATCCTTCCCCCGCATGCCAAGATCTCAGACGATGCCAAGGAAACCATCCAGGAGTGTGTCTCCGAGTACATCAGCTTCATCACTGGGGAGGCCAACGAGCGTTGCCAGCGCGAGCAGCGCAAGACCATCACTGCTGAAGACGTCCTCTGGGCCATGAGCAAGCTCGGCTTCGACGACTACATTGAGCCCCTCACCCTCTATCTCCACCGCTACCGCGAGATGGAGGGGGAGCGTGGCTCTGGCTCCTCCACCACCTCCGTCATGAGAAGCGATACCTCAGTGAAGGGTGCTGCTAGCAGCAGTAGCAGGGCAATAGATCACCACCAGTACTATGCTGCTGCTGCTGCGGCGGCTGCTTTTCATCATCACCATGGCCATGCCTTTTTTGGATACTTAAAGCCATCCGATGCCTCCACCTCCACCAATAATAATATCAATGCTGCTGCTGCTGTCGCCGTGGCCGCTCTGCCATATTGTGATCAGCACCAGCCACATGCAAATGATCGAGCTTAA

>PpNF-YB5

ATGGATCATCAGAAGAAGAGAGGGTACCGTCCAAAGTGGGCAACAACAAAGTTTAAGTGCGAGATAAATTTGATATTCTTATTTTTCTACACTCACTCACTGTGGATGGATCATGGAATCATTGATGTATGGTTGGTCCCATGCAATTATGAAATGAAGTTGAAGGGATGCTCCTCTCCTCCTCCTCCTCATCAAATCGTAAGGCTGACGAAATGAGGTCAATGCCTGCCTACCCAATTGGTGAAAAAGGGTAACCCACCATCCATATCGATGGACCACGTTGTTTTGACTTCCATTACTTGTAAATATTAATATAAACTCGTCTCTCTAATGTGGCAGTGCGGTTGGATCGGGTTTAATTAGTACCCAAGAATCCAATTAAACAAGGCCCTGTGCCACAGCCACACACCCCTCGTGGTCGGCTTGTGCGTAAAAGTCATTGAAATTTTTAGCATCATTGTCGTATTTCTCTATATTTTATTATAACACGTCGTATACATTACGTGATGGTATGATCGTTATACTAACTCTTTTATAATCGAGCTTGAATACAAAGTGAGTGTCAAAATCTTATCGCGATAAATGAACGGTCCGAGACCATTCAACATATCAGAGACCCAGCAATATAATCCCTCAAGATCCCAGCTTAGCTAGGAGAAAAAGCAGTCCAACCCCTCCAGGAGGAGGGCAGAGCATTAGAAAAATTGAATTTAGCATATAAAACTTCCTTTGCTATAACTCTTGAATACATCTAGAATTCACATTCATTAATTCATTTTTAAATGAATCAGTTACTGTTTTCAATTGAAAAACCCTTGCCTATATCATATAGATATATATATCGCTGACTCGGACAACGACTCGGGAGCAGGAGGAGGAGGAGGAGGAGGAGAAACGAATTACACAAGATCTTCATCATCCTCAGCGAGATAGCACGACAGGTTTTTACCGATTGCAAACGTGAGCAGGATGATGAAGAAAGGTTTGCCAGGCAACGCCAAGATTTCAAAAGACGCTAAAGAGACGGTGCAGTAGTGTGTCTCAGAGTTCATCAGCTTCACCACGGGAGAGGCCTCAGGTAAGTGCCGGAGAGAGAAGAGGAAGACAATCAACGGTGACGATCTATTGTGGGCCATGACGAGGCTGGGTTTTAAGGAATACGTGGAGCCTCTCAAGATTTATTTGCACAAGTTCAGAGAGATGGAGGGTGAGAAGACTGCTGGAACAGGAATGGCTAGGCAGCTGCTGTACGCTGATCATCATCCTCATGAATATCATGATCAAAGGCAGAGAGAGCAGTTGCAGCACCTGCATGCACCTGGGATGAACATGTATGGTGGGATGATGATGGGTCATCAACGTCATCATCAAGGTCAAGGGCATCATCCTTATCTTTATGGATCTGGTAGTGGACCCTCTAGTCTATAA

>PpNF-YB6

ATGGCGGACTCCGACAACGACTCGGGCGGAGCGCACACGAAATCAAACAGCGAGCTCTCGCCTCGGGAACAGGACCGGCTGCTGCCGATTGCGAACGTGAGCCGGATCATGAAGAAGGCGCTGCCGGCGAACGCGAAGATCTCCAAGGACGCGAAGGAGACGGTGCAGGAGTGCGTGTCGGAGTTCATAAGCTTCATCACCGGCGAGGCCTCGGACAAGTGCCAGAGGGAGAAGCGGAAGACGATCAACGGCGATGATTTGCTCTGGGCGATGACGACGTTAGGGTTCGAGGAGTACGTGGAGCCTCTCAAGGTTTATCTGCAGAGGTTCAGAGAGATGGAGGGGGAGAAGAGCAGCGTGGCAGCGCGTGACAAGGACGCGAGTGGAGGTGGCGGGAATGGTGGGGTTGGGTACGAGAGTGGTGGTGGGAGTGGAGGAGCTGGGGTATATTGGCAGCAGCAACAGCAGCAGCAGCAGCATCAGCATCAGCATCAGCATCAGGGACACGTGTACGGCGGGAGCGGGTTTCATCAGATGGGTGTGAGTGGAGTTGGGTTGGGGAAGGGTGGGCCCGGATCTAACATGGGTAGACCCAGATAA

>PpNF-YB7

ATGAGAGGGCCTCCTAGCAGCAGGCATGCAAAATCGAGCTCTGGTAAAATCTCTCTGATAGCAAATGCCATAGGGCTTTTTTCTCTTTTATTAATTTATATATATATCTTCTTTTCTTTTCTGTTTTTTTATATGTTTTTTCTTGATGATGGTACATATGTATGCATAAAAAAATAATGGTTGCGAGATTTCAGATTTTTCTTTTTGGTTTTCTTATTTGTTTATGGATTTTTATTGCATGCATGTCTTTTTCTCCTCAAAATTCTGAGCACAAAATTTTCTTCCTCACTCCCTTTTTTTTCCAGAGAGTTCCAAGCCCGATCTCAAGGTTTCTGCTTTCGATCCTGCGTTTGTTGATGTTTTTGCATGTAAATCTATGTTTTGTCTCAGCTCCTCTCGATATCACATGTATATCTGTCTGATCTCATTAGGGTTTTCATTGTTGTGTCAAGATTTTGACACGAAACTTGTTGTAGCTGATTTTTGGGGCTTGAAAGAGTGTGTTTTTGTTTGTGTTTTGGAATTTCTGAAGGGATGTTATCTTTTTTTACCTTATCTTGCTGTATAGCTGGTTTCAGTGCTTCAATCACTGTAACGCTTGCTCAAATATTGGTTGTTTTCTGTATAGGCCGTCTCAGGAATATCTGCTTCTATTCTATATGGCTTCATTTTCGCATATCTCAAATGAACGTTTCCCTTCCGCGCCCTTTTTATTTATTTATTTATGTATTTGTTGTTTTTTACTGATGTGGCATTCATGCAGCAGCACTGTAGAGTCTCATCAAGACCTAGGCTTGCACCTGCTTTATATATCATCTATGAATCAGATATGATCTGTTATTTCTAACTCATATATCTCTACTGGCTTATTATTGTCTTATTTTTGTTTCTAATGTAAAAAAATATATATAGTTATTCTCTCGTCTGTATGTTCGCACATTTTATATATATATATATATTTCATGAGATGCCTTTTATTTTCATTGTCGCAAACACCACTAACTTGGTTTGCTTTCACTAAAAACATCTGTATCTTTTTGTCCAAAAAAATATACATATATATATATATATAGTTTTTTGGTGAAGAAAAGCCAAATCAAAGAGGCTTCAGTTACGTCCATTTTTCTTTCTCGAAAATTTTACAAATGTCAGAAAGATTTATGTCCTGTATATAATAATATATATTCGGATTGAAAATTTGAAATAGTATGATTAAATCCCAAGTAGACCAGCTAAAAAATAGCAGAGCACATGAAAAACCTTTGAGGAGCTGACAAGGTCTTTTGGTGTTTGGCGAAAAGGAAGAAGAAGAAGAAACGAGTTTTGAACAATGGGAGTAGGTTAGGTAGGGCCCACCTTCTGTTTCTTTAAAATGAACCGTTTTCACATACACGGTGCCACGTGGACATGCAAGCGCGTGAGGTGTCAGTTTATTCTTCTCTTTTGAGCTTAGCTGGAGCACTAACAGACAAACAAAACAGCGTGAGCATTGAGAAAAGACTTGGACCTTTTTCTTCTTTTGTCTTGACTAGAGTAAAAGGGAGTCTGATGACATGTCGACGCGCGCGTCCATGCGTGGGGTCACATTTGACGTCATCACCTCCTGAACACCAAACCCTATTTTGTTTCTCAATTTCTTCTCTCTTGTTAGTAGATTAGATTAGCGTTATACATGAGGGTTTGGTCAATAACTATGCTTATTATTTAGATGGTCAATTGTTTATGTGTGGTCAGTGACTAATTGTCTTTACTTGCGTGTGAATAATTAACTATTATTAATTGATATGAGTCTTACAATGAATTTTACGTCAGATTCAAAATCTTTGATATTTTTGACATACATTGTATTTAGTATTAGCATAGGCTGGATATAATGACTTGTAATTGCATGCATCTTGGTTAATGATCTTCCATATGGAGCTTTTATATCGTAGAAAGTGGCAAAACCATTTTTTTCTTTGGTCGAACATGGTTGGCTAAACCATCTTCCATTTCTGCACTGCACACACACACAGAGACAATTAATAAATTATTCACACCCATAAATGTACACCCTGATTACCATAACCATATCTTCCTCACCCATTAATAAACTGACCAATATTGTTACTTTCAAATAATATATATATAAATAAAAGCTTGTTAATTGGTCTCTCATTTATGAACGTATCTGTACAGTGATTAATATTGCTCGACTTACATGAACTGTTTTTTTCAACCTTGAACGCAGCACAAGTCAACAACTCCGAGAGGAGCAACTCCAAAGTCCCAATCAACGCCACCCCAGCAGCAACCAACAGCAACGCCGCTGCTGCCGCAGCCAACGCCGGTGATCCTGCACAGCTGGCCCCACAACAATCTGTTGTTCGCGAGCAGGACCAATACATGCCTATCGCGAACGTGATCCGCATAATGCGTCGTATTTTGCCACCCCATGCAAAAATATCTGACGACGCCAAGGAGACCATCCAGGAGTGTGTGTCCGAGTACATTGCCTTCATCACTGGGGAGGCCAACGAGCGCTGCCGGCGAGAGCAGCGCAAGACCGTCACTGCCGAGGACATCCTCTGGGCGATGGGGAAGCTTGGCTTCGACAACTACGTCGAGCCCCTCACTCTTTTCCTCCAGAAGCACCGGGAGTCGGAGAACAGCGACAGGTCCACTCTGAGAGCTGAGTTTATGAAGAGGGATCGTGCAGTGGATTTTGGACCTGCTGGGCCCCCGATTGCTCTAATGCCTCCTCCTCCACCGCCTTATGGGCCGGGCTATCCTTTTGGGCCCCAGCATGGTCCTGGGATGTTCGACCCATCCATGCTTGGGATGTTTAGGGATGGGTCGTCATCTGGCTCGGGCGGAGGAGCTGGGTCTTCTTCGGCTTCAGGGGATCAGGGCCAGAATTCCTTGGAGGGGTTTGATCCGTTTGCTCAGTTCAAGTGA

>PpNF-YB8

ATGGAAGATGAGAGGCTAAATGGGCCAAGTGGAGGGAGCCCAGAAAACCCATGTGGTGTAAAGAGCAGTGCAAGTAGCAATAACAACAACGGCAATAGCACTTACAATAACAATAACAATAGCAATAAAGAACAAGATAGATTCCTCCCCATAGCAAATGTTGGTCGCATTATGAAGAAAGTGATCCCAGGCAATGGAAAAATCTCAAAAGATGCAAAGGAGACTGTTCAAGAATGTGTCTCTGAGTTCATAAGCTTTGTCACAGGAGAAGCTTCTGATAAATGCCAGAGAGAAAAGAGGAAGACCATCAACGGTGAAGATATCATCTGGGCCATCACAACCCTCGGATTTGAGGACTATGTACATCCATTAAAGCTCTACCTCCAGAAATATAGAGAACTTGAAGGGGAAAAGCTAAATGTTCCAAAGCAACAAAGATTGGAACAAAGGCAGCTGCAGCAACACCAACAAAACCACCACCACCAGCAACCAGAACAACAAGAACAACAAACTTTAGTATCTGCTTACGATAATAGTGTATATTCTTCCACCAATCTCCTTTCTCAGCCATCTTTCATAGCCGCTGCCGCTGCGGATCATCATCAGACAGCCTTCTCTTTGCCCTTCTCCCCAACTTCAATTCAAAAACAATTGCACCCACAAGATCATATTGATTCAGTGGGGCATTGGTAA

>PpNF-YB9

ATGAGTGCAAGAGTGAGTGAACCGTTGAATTTACATCCAACAGTGAGTTAGCATAAATCTCTTAAACCTCTGTAGTTCAAGAGATCGGAATAATTAATTATGTATACCTTTTCTAAATATTTTTTTTACTCTTTGGGCGGAGGATATATTTTTTAATAAATATTACGATGTATCATCAAATCCGAAGAATGAGATTAATCCCCATCCAAAATTCAACTTGCCCCGGCTACAATGTTCTTCTAAGACCAACTCCAGCGGCACAGCCCAGCCCGAGGCTAAGGGAGAAAAAAAAAAAAAAAAGGTGTTCCAGCGCACAGCCCAGGCCCGAGCGCAAGGTGGGACCCAGCAACCTGGGCTGGCCCGAGCTCCAGCCCTCGTTTTGGTGCTGACGTCATCGCTACAGTGTCTGGCGCTACAGTACCACTAAAAGTCCACGTGTCGCACTCTGGGCTCTCCGATCAGATTTTTTTCTCCAATCCAACGGCAGCCAATTTTTGTGCAATAAAAAAATGAAAAAATTGAATTTTTTTTTAAAAAAATACCAAAAAAATACTCTTTTTTTTCTATAAATACCAAAATTTTATCCGATTGAGATATGAATTTTATAATAAATATTGATATGTACGAACCCAAAAATATTATCCGAAAATATTATCTAAATTAATTATTTTTATTAAAAAATTTGTGAAAAAAACAAAAAAATAAATAGTAATTGCCCTTAGCCATGGCAATGGGTGGAAACACAAAATACTATTTGTAAGGGCAGCCACTATTCACGTGAATAGTGGCTGCTCTAGCTCCACCCTTGTCATGGCTAAGGACAAATAGATGGAGTTGCTCTAAGGCGGAGAACATATTGATGAACCGGTACTCCATTTAATTTTTGTGAGAATTAACATTCCCATACTGCCATTAAGCAAGCCAAAATGAAAAGAACCCGACATGATTTGAAAAGCACTCCAGCCTTGTTGGCTCACTAGCTGCCAAGACATTTTCTTTCACTCTTTGTTTAGCTCATATGCTTTTCTTTCTTTTCTTCTTTTAATGGTCAAAACATGTTGGGGCATTATTAAAAGAGATATTTAGTTATAGTCCTGATCTCTTGGATTATAAAGGTTTAAGAAATTTGTGGTCACTCATCATTGGATATTTTTATGCCATATTAAGTATTTTGGGTTATTTTATTAGGTTTTAGAGTTGAGTCTTAGGCAAGGTTCTATCTCTTAATCGTTGGATCAGACTAACTAATCTGATCAAACGGCTAAGAGATAAAACCTCACCTAAGGACTATATATAAGGTCTTCATCATAGAATTCTTGTATTGAGCTATATCCTACATACCGTTTGAATTTTACGAGATATATTGACCCAACAAGTGATCTGATATATTATAGAGCTTAAAATTTGTGTTCGTACAAATTAGCTTAATATAATGCAGGATTGATCCTGCTGCAACCTTAATTGTCAAGCCCTATTACCTAATTTTTTAATTGCTATAGTTGTCGTGTGAATGATGGAGAAAACCAATGTAGAATAATGAACTCATATTATTTTTTCAGTTCATTAGCGGGACTAGCGGGACTGCAAGTCACCGAGTCAGCATGCACATAGACATGTCCAAAATTACAGAGAGGCATATAGGTAGAGAGGCGGTTCCTTAAGAAGCCATTTGTCGCAAGCCTGATCAAAAGGTATTTATTGAGTGTATTTTTATGCTTATTATTTAGTTTATTCAGATTTTTATGGTAAGATATACTGTTTTTAAAAGTTATAGGCAGAGATTATCAATGAACTGAACGAAATTAAAATACAATTACTTGACCAAGAACAGAAAAGGGACGACAGGTATAGTAATTATTAAACTAAGCAAGTACAAGCACATCATTTGCTAAATTTAATATGATGGATTGGGAGACAGCAGATTTGCACATTTCAGAATCGAAACTATATTAGAGAGAGGACAATCGAACACACGATTTAGAGTAAATAGTAAATATTCTTAACTATTTAAGCTACAATTCTCTTACATGATAAACTGTGTAATTAGGACCTAATTAGTTTGAGCACTCATTTCTAATAAATAATAATCTTTATTTGTAAGAAATAGCGGATGAACACGTGATAAAAAAAAGCATGCCTTCGGATGCATGAGTAACTGCTCTTAACTATTTGAGCTACTGACCCTTTTCACACAATAAGAAAAATCTTAAACGTGCACAAAGAGCTCTACTAAGAGGCAAGTTTAACTAATTAGGAGGAAGCTATTGAATTAAACTGAGATTGGAGGGATCCAATTGCATGTCTTGAGAGATTGAGAGTGGCAGGCAGAAGATAGTCTTTACGTCTATAGATGTGACAGAGGGAATCTTGGAAAGACATAGAGGAAGATAGAGCTAAGCTAAGCATTATATGCGCCTCCCTCTTTTTGCTTTGCCTTTTTAAGCCGTGCAAAGATACCAAGTCCTCCATTACAGAAAAACAGAAGATATTTCAATGATCCATTGGTATAAATTGAAGTCAAAAGATTTTTCAGTTTTTTTTATTTTTATTATGGTCAGTAAATTGAAGTCTAAAATTGATCAACAAAATATCTCCAAAGATTGTTGAAGACAGGAACATGGGATGGTTTCTGGCTCCTATGAATGTACCAAAACTTTTATTCAGGATATATGCCACCTATATTTGTTACTTAGCACTGAAGAAGGGCCTAGCTGCTCTAGAGACAAGGCCAACTGGTTGATGATGTGGCTGATTCCTTGGTTGACAACAAGGCATGTAAACTGTCACATCGATGTGTTATGTGCACTGGCTGGTGCTGACAAGGCGAGAAATTCTCATACAACGGAGTAGCACTTTTTGCTATTCTCGGCCTATAACACAATGACACATGAGATAAATTTTCCATGTGTCATTGTATTATTAGCAGAGGATAGCAAAACAGTGCTATTTCCGTTTAAAAAAAGTTTTTATTTTTGCCAGGAAGCCTTAAGGCAGTTGGGGTTCTCAACTGCTAGACTTAGCTGTTTTATACTTATTGGTTCAGCAACCATTGGGTAGATGGAGGGGGTGTCAATGGTAGAGAGGCAAGTAATTAATTAATTCAATACAAGTAAATTACACTTGAGAAAAGAGGCAATGTGACTTGTACATCAGTGCAATGGGTTGTGCTCTCTTTATTTTTTTTATTTTTATACGACTAATGTTAAGGGAAAATCGAACCTATGATCTCGGATGCAGAGTTACATACAAGCGATATATATATATATATATATGTATTTCTGATTAAGTAGTTGAGATCAAACTCTAGGTTAAGAAAGACATGCCTGGAAGAAATTGAAGTTGAGGTCAGTTCCGGCAGTGATAGCTTATTACACACTGCACCTATTGGACATGTTCAAACAAATTTAAGGTCTCCTGTGCAGGCTTTCTATTTTTCCATTGCCATTCCATTTGAATTTTAAATTATAGTCAAAGGTACAAAGGAAAAGGACCTGCAATGCATATAGCAAACAAGTGAAAATCAAACAGATTAAAGGAGAACCAATACATATATTTGGGAAGAGTTTGCTTATTTTTCATGTCCTACCTCCAATTCTTCTTCTTGGTCCCATGGAACTTCCACACCAGAAAAGGTCACAGTATCAATTCATTATTTATTTTCTTTTGTTATTATTTAACACCACATGTAGTTTTTTTTTTCTTCTTCCTCCAAAGATTCCACACCTTCCCACACCATCCTTTGCAGTTGTTCTAATCTCCCAGTGTTTGTTTTTCTTTTTTGTCCACATCAAACCGCCTCTCCACTCTCTTTTTTATTTAATTTTTTTTACCAAAGATAAAATAAACAATAATCTATTTTGCTTCCTTTTTCCCCCACTTCTGCTAGCTTTAAAACCCCAGACTCTTCCCCTTAGATTCCACAACAAACACTCAACACAAGAAAGAAAAAGGAAGAAAAAGAAAAACCAGTACACATTTTCCTTCACTTGCATTTCCCTTTTCTATTTCACACAATCACTCTGAGCTTAACTCTATGAGTGGAAGAAGAAACCAAACCAGCCCGGTTGGAAGCCCTTTGTCTGGGAATGTCTCAGACGGCTCTTCCAAAGAGCAAGACAAGTTCCTCCCCATTGCTAACGTGAGCCGTATCATGAAGAAGTCCCTCCCTGCAAATGCCAAGATCTCAAAGGAGGCCAAGGAAACCGTCCAGGAGTGTGTGTCTGAGTTCATAAGCTTCATCACGGGTGAAGCGTCTGACAAGTGCCTGAGGGAGAAAAGGAAGACTATCAATGGAGATGACCTTCTTTGGGCCATGACAACCCTTGGATTTGAGAATTATGTAGGACCCTTGAAAGGGTATCTCAACAAGTATAGGGAGACTGAGGGGGAGAAGAACTCCATGACTAGACAAGAAGAAGACCCTTCTCAACAACAGCAGCAACATCTCAACACTTCAAACACCAATATGCAGCATAGTTCTAACAATGAGCAAATGAACACAGTTCTTAATGCCAATAATATTTCTATGTCAACCTCTAAGGTGGATCTTTTCAATGGTGGCTTCTATTTTCTTGAGGGACAACAGCAACAGCAGGAAGTGACTCAAAACTACAATTTGGTGAGTGCTGGGGCTTATAATTTGAGTAGGATTAATGAGAGTGGAGATGTGAATGGAAACAGAGATTTGGCAACTCATCATCTTCATAATGGCATAGGATGGTAG

>PpNF-YB10

ATGGCCGAAGCTCCGGGAAGTCCAGGCGGCGGGAGCCACGAGAGCGGCGGCGACCAGAGCCCGCGCTCGAACGTTCGGGAGCAGGATCGGTATCTCCCGATCGCTAACATTAGTCGGATCATGAAGAAGGCGCTTCCTGCTAATGGCAAAATCGCCAAGGACGCCAAAGAGACCGTGCAGGAATGCGTTTCCGAATTCATCAGCTTCATCACTAGCGAGTACGACGCCGTTTCGTAACTCTGTTCATGATTCATGATTATGTTTGGAAATTGTATTTATCCTTTTTTGTTTTGATGTCGGGGTGGAATTGTGTGCAGGGCGAGTGACAAGTGCCAGAGAGAGAAGAGGAAGACAATTAATGGCGATGATTTGCTCTGGGCAATGGCTACTTTGGGCTTTGAAGATTATATCGACCCCCTTAAGGTTTACCTCACTAGATACAGAGAGGTAACGCTTTTTTGCTATTTGGTTTCGCCTTTTCTTAATTTGGTCTCTAATTTCGAGTTATTGTACCCAATTAAGCTCCGAAGTTCAGATTTTGTTGAGAAATGAGTGTTTGCTCATCTCAATACTGTTGCAGGCTCGGGTTTGTTTAACTATTTAATGACAAGATTTGACTAATACACTGTTTCTTTTCTGTTGTCTTCTGGGTCTATTCGGACACTGCAGATGGAGGTGAGTTACAATTGTTTTCTATTTATGCGCGGGTTTTTATTTTACTTTCTGCAGTTTGTTTTTATTTAACCTTCTCACTTACTTTTGGGTTTCGGCTTGTGACGTCTAATAGGGTGACACCAAGGGTTCAGGGAAGGGTGGAGATTCATCTTCTAAGAAAGATGCTCAGCCAAGTTCAAATGCTCAGGTTGAGTATGATTTTGTTTTATTAATGAAGCAAGGTTCTTTTTTTGTGTTTTGTTCTCGGGTTTTAATTTGTCTTATTTTCATTTGGCAGATTTCTCGCCAAGGTTCTTTCTCCCAAGGAGGGAATTACTCAAATTCTCAAGTAATTTCTTTCTCTTTGTCTCTCTATACCTATCTGTTTTAAAATATTATTATCTCTCTTCATCATGTTTGTGTAATACTTATCGGTGTTATAATATTAACCGGATTCCTTTATTGCCAACACATTTCCGTATGACTTATAATGATTTCCATCCATGTATACCATTTTCCTTTGATATTTTGAGCATGATTTCTGTATATTCAAATGATGGCCCTTCAGACTTAATCATTGACGCAATTTGTTTTCACAGCAGGATTTATCTTATTATTGGCGTGCGTTGGTTGGTTTTGTTTCCTTTGGTTTAAGTTAAAATTTTGTGGTCATTAGTGCCTAATGTTTGAAAAGTTGTGCTTTTCAATTTTATATGTAATAGTTGTGCATGTCATTTGTAGAATTGTGATGATATGCTCAATGATCATTTGTCTGTGGAAAGTGGATGACTTTTACTTTGCAGAATAAAGTGAAAGCTGCACAGTAGTAGGGTGCATTAAACCGTTAAACATATATGGTTTTCGTGTAGGTGCTCTTGTTTCTTGTACTTGTTCCATAGAAACGTTTGCATGCCCTGAAGCAGCAATGTAAATTTTGTCCCACTTGGAATCTCTTTTGGAAAGTTCCCAGTTTGCTTTTCTCCTAAGTTTTTACTTGTTGCTTTATAGTTGGTGCTTTGCTGCAGAGTTGTAGTTGCGGTTATTGGGTCAATAATATTATTCTTTATAGCATACAATGTTGGTAAGATTATAGCATTATAGCATTGTGGATTATATCAATGTTGGTGGATTATAGCATGCAATGTTGGTGGATCTATATGAGCAACACAAGGCATTGTATTTTCAACTTAGCTTTCCAATGGTTGGGACTACTGTACAATGTTAAAACTTCTCGATAGGGAGAGCATGAGATGTGGGAGTGGCAAAGCAACTGATGAATAGTAGTGTCATTATATTAGTATGTTTGGCTTGTTCTACAGTTTTATTTATTTTTCGGTTTTGGAAGGTGCTGTTTATGAAACTATTGAAATGTTTTTGCTAGTGGTGAGTAAGTATGCTGGTTTTCTGGTTCTTGTACTCAACATGGTCTAATGAATGATTTGTTATTGCAGAGCCAACATATGATGGTTCCTATGCAAGGGACAGAGTAG

>PpNF-YB11

ATGGTAGATGAACAAGATGGGTTGCTGCCAATTGCAAACGTGGGTCGCATCATGAAGCAAATTCTTCCCCAAAGAGCCAAAATTTCCAAGGAAGCCAAACAAAGAATGCAAGAGTGTGCAACTGAGTTTCTAAGTTTTGTGACGGCTGAGGCGTCTGACAAGTGTCACAAGGAGAATCGCAAGACCGTGAATGGAGATGACATCTGTTGGGCTCTGAGTGCTCTAGGTTTTGATAACTATGCCCAGGCTACTATAAGGTACTTGCATAAATATAGGGAAGCTGAGAGAGACAAAGCTGCTGCAAATGCTAACAACAACAACCAAAACAAAGCAGCAGATATTATTTTGATGAGCAGCGGCCAAGATATGAACGATATCGGAGATCGAGCAGCAAGTATCTACATGGCAAGTCAACAAGGCCTGCAGGTAGGGGAGCAAACCCAAACTCCAGATTTGGAGTTTCGGCTGCTTGGGAAAGGTGACGGCACCAAGCCATCTGTTGATCAAGAACATAATTAG

>PpNF-YB12

ATGGATAATAACATAGGCAATAATTCATCAGAGAGAGAAGGGTTTAAGTACAATTTCACAGGCAGTAGTATCTCTAGCGATCATCATCATCATCAAGAAGATGGGGTCATCAAGGAGCAAGATCGGCTGCTTCCGATTGCTAATGTTGGGCGGATCATGAAGCAAATTCTTCCCCCAAATGCAAAAATCTCGAAAGAAGCCAAAGAAACCATGCAGGAATGTGTTTCTGAGTTTATAAGCTTTGTGACTGGAGAAGCATCTGACAAGTGTCACAAAGAGAAGCGCAAGACAGTGAATGGCGATGACATTTGCTGGGCTCTTGCAACTCTTGGCTTCGATGATTATGCTGAACCATTGAAGAGGTATTTGCATAGGTACAGAGAATTGGAAGGAGAGAAGGCGGCTCACCAAGGCAAGGCCAACAGTAGTGAAGAAAAAAACGAACTGTCACCACCAAGGACAAGTACCTCTAGTCCCTTAAAGTTCAATGTGCTTGAAAGGGGAAATAGCTCCCTCTCCCGGCGATTTTGA

>PpNF-YC1

ATGGATCAGCAGGGGCATGGGCATCCCACAACAATGGGAGTGGCTGGTAGTGCAGCTCAAGCATCCTATGGTGTGAACCCATATCAGTCTGGCCAAATGATGGGGCTCTCTCCCACTGGATCAGTTGGATTGATGCAATCTCCTACTCAGCCGGCAGGTCCTCCAGCCTCTTCTCAGCTTGCACAACACCAACTAGCTTATCAGCACATCCACCAGCAACAACAACAGCAACTACAGCAACAACTCCAAAGTTTTTGGGCAGAACAGTATCAAGAAATTGAGGCGGTTATGGATTTCAAGAACCACAGTTTGCCATTGGCCAGGATTAAAAAGATTATGAAGGCTGATGAGGATGTGAGGATGATCTCAGCCGAAGCTCCAGTCATATTTGCCAGGGCCTGTGAGATGTTCATTCTAGAGTTGACACTGCGGTCTTGGAATCACACAGAGGAGAACAAAAGAAGAACACTGCAAAAGAATGACATTGCCGCAGCAATTACGCGGACTGATATATTTGATTTTCTGGTTGATATCGTCCCGAGAGAGGATTTGAAAGATGAGGTACTTGCGTCAATCCCAAGAGGTGGGAATCTTCCGGTTGGAAGTTCGACTGATGGTGTGCCTTACTATTACATGCCTGCTCAGCATGCTCCACAGGTTGGTGCTCCGGGGATGATAGTGGGAAAGCCTGTTATGGATCAAACTCTTTATGGCCAACAGACACATCCCTACATTGCTCAGCCAACATGGCCACAGCAACAGCAGCCGCACAAAGATTCTTGA

>PpNF-YC2

ATGGACATACCGATGGGGAAGTAAGTAGCACCTTTTTCTTTCAACTAAAATTCTAGATTTTGAAAATAAAATTCATTTCTATATAAGACTTTTGTTGCAGGATAATATGAATTATTGGATTTTGTCTAAAATAAAGTTCAACGGTTGATAATAACCAGAAAAACAATGGGCTACAAACACACAAAACGACTCTATTCATTGGATTCTCTTTTAATTATAAACACTATCCTGATTAGCAATATCTAGTTTCAGATTACATCGTGTCTCCTTTTTGACTTAAAACTTAATTGCTCATGGATTCTTCTAACTTAAAGACAAATAAAGTAAAAGCAGATTTAAAATGTTGCTTTCTCACTTCTGAAAACTGTTTAAAGTCTCACTTCACATGAAAATCAGGTGACAGAACTAGAAACATTTGAAGCCTTTGAAACCAGAAACGAGCTAGCTAGACAATTCAGGCCAGTGCTTATGGATGTGAACCATTCTATCAATATCACACCCTCTTCAAACACTTCTCCAGAAATGCACGGTTTCATGTCGACGGGCTCTTTCGAGCTACGCAATTACTACTCTCATCCGTCTAGCAGAGAGGTGAAGCTAATTGCCATCCATTTTCTATTAGACTAGAGATTGCATTCAAATAAATCAAGTATTTTCCTTCCAGTTTCTTATGCAAGATATATATCATGTGTTATTTTCTTCCTTCTGGTTTTTTTTTTTTCTTACTAATAATTAAGTAGGAGGCTGATCAGGAGGCTAAGCAATCCAGTTTTACGGAACTTCAGAAGGAAGAGATAGAGATATTCTGGAATCAGCAACTGTTTGAAATTCAAAACACAACAGGTTTGTTTTTTCTCTTGGTCCAAATTTTGATTTTTTTTTCTGTCTTTGGTCATTGCTACAGTTTTAAGCTAGTGCCCCAAAATAAAAACAAAAACATACAAAAGTTTAAAATTTTAAAAAAATAATAATAAAGGGATTGAACCCAAACTGTACTTTTATTGTACTTTTGAGTTAAAAGCATTACATATGATTTCTGTTTATTTATGTCGAAGTGTGCTTGTTTTAATTTTTTCTATCATAATTTGAAAACACCGTTAGCGCTAGCACACGTTAGTAGTTGTTAGCCATCATTTTTTTTCAATTTTTTGAATTGTTTTGAATTTTTTTTAGATGTTCCAAATCTTTTGTTTCTTTAAGAAGTAAAAATCATTACAAAAGGTTTTGATCAGGACCTACATTACCAAAGGGTTATATATATATATATATATAGATGGACCCGAAATTTTAAAATGAGCTGGACTAAATTTACTTTAGAGCAATTCCAGCAGTAAGGCTGAGGCGCGGGCTGGGGGGAAGTTTGGGCCAAAAAAGTGATTCCAACAACAGAGGCTTGCCCGGGCAACAGGTGGACCCTAGCAAACTGGGTTGGCCCTCAGGGGAGGCTGGCCCGACTTGGAGCCCGAGTTTTGGATGATGTCAGAGCGCTGACGTCAGTGCGCTACAGTGCCGCCCATAGCCAGCATCCACGTGGCGTTGTCTGGGCTCTCAGATCGGATTTTTTCCTCCAATCTAACGGCAGCGCATTTTTGTGCAATAAAATAATGAAAAAAATAGAAATTTTTTAAAAAAAATACCCAAAAATTAATGAATTTTTTTTTCTATAAATACCAACCAATACTCTTTACTTTTAACACCAAATCCTCATATATTTTCGTCTCTTTCAACTATTATTCACTTTCTATTTAATATTTTTTTCACTTTCAAGTTTTATTTTTTAAAAATCTTATCCGAAATTTTTGTCCGATTATGAGATATGAATTTTATAATAAAGATTGACATGTAGGAACCTAAAAATCTAATCTGAAAATATTATCCAAATTAATTATTTAGGTAAAAAAATTTGTGAAAAAAACAAAAAAATGAATAGTAATTGCCCTTAGCCATAGCAACGGGTAGAAACACAAAATACTATTTGCAAGGGCAACTACTATTCACGTGAATAGTGGCTGTCCTAGCTTCCCCTTTGCCATGGCTAAGGGAAAATGGGTGGAGTTGCTCTTATGTAAGCACTGGAGAGTGGAAGATAAAGGTGCAACTACTGCAGCATAGCAATTCGTTTTATTTATTTTCACTTAAAGGGATTATGTGTGAGGTCTATCAGTGAAAGGAAAATTAGTTAGGTTGTCCATAATAAAATAAAATAAATATAAACATAAATAAAAAGAAGTTGGCATGAGTATGCTAAAAAAGTAAGAACAATATGGAAATACTTTTCATTTTAGAGAGAACTTTACAGACATGTCCCACAAAAGAATGTCTATGAATATTCGAAGAGAATTCATCAATTCATTAATTTGTCTTGTCTTCTTTTCCTTGATTGTATTATTGAATAAGTGATGGCATGATTGTCATTAGTAGGCATGGGTGGGTTAAATTAAATTTTTTGGCCGATAAAATAACTACTCACCTGGGCTATAGCACATGTTAATCCCTTCTAGGGACCATCGGAAGCTATTCTTTAAATTTCTATGTAGTATAACTAATGATCACGTAATTCTTTATAATGTATGATACAGTGGCAAAAGCACATCACGAACTCCCTCTTGCCAGGGTCAAAAGAGTCATGAAATCTGATGGACAAGTCAAGGTAGATCTTCACATTCACCAATTGATATGCAAAACCCTTTTTATAACTTATTTATTTTTTATCTATTAATTAGAGAGTAATGTTTCAAAAAGATTAACTTGAAAGTGTGTTAATTTTCTTTGCAGAAGGTGAGCTCGGAGACTCCGGTTTTGTTTTCGAAAGCATGTGAGCTTTTCATTATGGAGCTCACACTTCGTTCATGGCTGCACACTGAAAGAAGCAAGAGGCGTACGTTGCAGCATTGTGACACTGCCAGGGCAATTATGCAGGATGAGCTTCTTCACTTTTTAGTTCACGCCGTTCCTCCGCTGAACTCCATCGCACGTGACTATTTTGAAGGTGACAAATTGCACTTTCTTCTCTTTCTCCTCCATCTTAATTTATTTATTTTTCTTCTGAATAATTTTATAAACAATGCAGATGATGAATGA

>PpNF-YC3

ATGAATCACTCAGAACAAACACTACAGCAACAGCAACAGGAGCAACACCATCAGCAGCAGCCTGTGGTGGGTGTTGTGGCAAGTGGAGGCCAAATGACATATGCACCTCCTTCCTATCAAACTGCTCCTATGGTAGCTTCTGGAACTCCTGCTGTGGCCGTCCCTTCCCCAACACAGCCCCCTGCCGCTTTCTCTAATTCCTCGCATCAGATTGCCTACCAGCAAGCCCAGCATTTCCACAACCAACAGCAGCAGCAACAACAGCAGCAGCTTCAAGTGTTCTGGGCCAACCAAATGCAAGATATTGAACAAGCATCTGACTTCAAGAATCACAGCCTTCCACTTGCAAGAATTAAGAAAATAATGAAAGCTGATGAGGATGTTCGAATGATATCTGCTGAGGCTCCAGTGATATTTGCAAAGGCATGTGAAATATTCATCCTGGAGCTGACTTTGCGCTCATGGATTCACACAGAGGAGAACAAAAGGAGGACGTTACAAAAGAATGATATTGCAGCTGCTATTTCGAGGACTGATGTCTTTGATTTTTTGGTTGATATTATCCCAAGAGATGAGTTGAAAGAAGAGGGACTTGGAGTGACTAAGGCTACAATCCCGGTGGTGGGCTCCCCAGCAGATATTCCATACTATTATGTACCACCACAACATCCTGTGGGAGCTCCTGGGATGATCATGGGGAAGCCAGTGGATCAAGCGGCAATCTATGCTGCTCAACAGCCTCGACCACCTATGGCTTTCATGCCGTGGTCTCAGCCTCAACCTCAGCAGCCGCAGCAACAGCAACAGCAACAAGAAGCCCAACATCAGCAGACAGATACTTAA

>PpNF-YC4

ATGAGGCAACCGGGAAGGTACTCAGGATTCATGATGCATGGAGGCATATCTGGGAGGACTGGGCCCCACTCATTGCCCTTGGCGAGGATCAAGAAGATCATGAAGAAGTCCGGGGAGGACGTGAAGATGATATCTGGCGAGGCTCCAATTGTGTTCTCAAAGGCATGTGAGCTGTTCATAGAGGAACTCACTCGGAGGTCTTGGATGACAACCCTGGAAGGGAAGAGAAGGACACTGCACAAAGACGACGTTGCCTCGGCCGTCGTAGGCACCGATATCTTTGATTTTCTTGTGAGTTTGGTTTCGGATTCTTGCCATTCTGAAGACATCACGCCGGCGGACAAGGAGGCATTGGGGGGATCATAG

>PpNF-YC5

ATGGACACCAACCCCAACACCACCACCACCACCACCACCCCCAACCCCACCCAGCAACAACAGCAAGCTCAATCCTCCTACCCACCAACCCAATCTTCAGTCCCTGCCCCTCCATTTCATCATCTCCTCCAGCAGCAGCAGCAGCAGCTGCAGATGTTCTGGACCTACCAGCGCCACGACATTGAACAAGTCAACGACTTTAAGAACCACCAGCTCCCATTGGCTCGCATCAAGAAGATCATGAAGGCGGATGAGGACGTCCGCATGATATCGGCCGAGGCCCCCGTTCTCTTCGCCAAGGCCTGCGAGCTGTTCATCCTCGAGCTCACAATCCGCTCTTGGCTCCACGCCGAGGAGAACAAGCGACGTACTTTGCAGAAAAACGACATTGCCGCCGCGATTACCAGGACCGATATATTCGATTTCTTGGTGGATATTGTGCCCCGAGACGAGATCAAGGACGAGGCGGTGGGGCTCGGGGGGATGGTGGGAGCCACGGCAAGTGGTGTGCCGTACTACTATCCGCCGATGGGTCAGCCGGCTGGTGGGCCCGGGGGGATGATGATTGGGAGGCCGGCGGTGGACCCCGCTGCAGTTTATGGAGTGCAGCCGCCGTCTCAGGCGTGGCAGTCGGTGTGGCAGACAGCGGCGGATGATGGGTCGTATGGGAGTGGTGGCAGCAGTGGACAGGGCAACCTTGACGGTCAAAGGTAATGTATGTGTCTATCAAAATGTGTGTTCTTTATGGTATGATTTGATTATTCTTGATGCGGGTATTGCTGGTTTATTTAATTGAGATTTCCGTGAGTTTAGTTATTGGGTTTGTGGCAATTTGATGCTTCTCTGATAAATTACCTTCTTGTAGGTGGTTTTTTGTTCTTATTAGGCTGATTAGGTAAAATGTGGACCTTCATTTGTGATTTTTGAATCCAATGGTTTCTTTCTCAAATAGTGCTAATGGAATTCGTTTCAAGGAGCTAAAGTTTTTAGCTTTTGGGTGCATAAATGTTGTTCATTCCCAAGGAGGCCTTTTGGCTTTTGAATCTGTAAACTTGAAATATTTCTGTTTCTCTTGCACCTGCTTGTGAGTTTTCATTGTTTATAGTGTGGGCCGCCTCGATGCTTCCTGCACTAAAGGAAAATGGAAATCCTAGATGCATGCAAAAATCTGGATATTAATTTCAGGGGCAATTTCATTAGGGTAAAAGGAAGCCACTTTTGTTTATTAGGTTATTCAAATTGATCAACGTGAAACTTGACTCAGCCTTTTGCATCCAACTCTTACAAGCCAAGTGTATTACTGTGCTTTTATGGAATTCAATCGCATGGTGGTTTATACGTATGCTTGTCTGGGGAAGAGAAGATCAAGGTGATTCCACTTGGTCTTCACATAACGCAAGAAAATTGGTTTAATTGATTTTAGGGCTGTGATTTATAGTTTTCGTGGGAGAATGCACCAAGAGATTTCATGGTTGTGACTTGAAACTGCTTGTGTTTAACACGATCAGTTGCTGGTAGCATGGACCTGTTTGTATTGTTAGTAGAAATTGATTCCCATGAGAGCCATGAAGTTTTCTTTTGTTCTCTGCATCCCCTCTAGGTGAAGAGGGTTTTTGGATTTTCTTGTTGTGGCACGTGGCACATATTTATAATACTCTTGTTTGTAGAATTCATTTCATGTTTTCTTTTTATCAATCACTTAGAGGAGCACTTTGCAGGGCAAAAAATTGAAATTAAACGGTTTTGTGTTTCCATGATGAATTAACTTTATCTTCCAAATGGGGTGCTTTTTTGTTGTTGTTGATGTATTTAAAGTAAGTATGCATTAAAATGCTTGTATTTCGATGCAATGGTTAAAAATGCTAGTTCAATTGCGACAAGCTTAATTTTGGCAACTGTTCTGGGATTTTGTTGTGAAATTTCCTTAGCTGTATTAGAAGTATGATGCTGCAAAGGGAATTTAGTTCCTTGGGTGCTTTAGTAAAGGTGACCTTACTAATTGAATGTATATTATTGAACTTTTAGTAGTATGCCTAAGTTGTAACTCCAAATAGTTACTAATTCCAACTTGTTTTTTTCTCAATTTTTCTATGTAGTTAA

>PpNF-YC6

ATGGATCCACAAGGGCATAACCAACCCCAATCTATGGGGATGGTAGGCAGTGGAGCTCAATTGACATATGGTACTAACCCATATCAGCATAATCAAATGGTTGGGAGCCCAAATCCGGGGTCAGTTGCCGGAACAGTGGGAGCTATTCAATCAACTAGTCAATCTGCTGGAGCTCAGCTTGCACAACACCAACTTGCTTATCAGCATATCCACCACCAGCAACAGCAACAACTTCAGCAACAGCTGCAATCTTTTTGGGCGAATCAGTATCAAGAAATTGACAAGGTAACTGATTTCAAGAACCATAGCCTTCCCTTAGCAAGGATCAAGAAGATTATGAAGGCTGATGAGGATGTGAGAATGATATCAGCTGAGGCACCTGTAATATTTGCCAGGGCATGTGAAATGTTCATATTGGAACTGACATTGCGGTCTTGGAATCATACGGAAGAGAATAAGCGGAGGACACTTCAGAAGAATGACATTGCAGCTGCAATCACAAGGACTGATATCTTTGATTTCTTGGTAGACATTGTGCCAAGAGAGGATCTGAAAGATGAGGTCCTTGCATCAATTCCAAGGGGAACAGTTTCTGTAGGAGGGCCGGGTGATGCACTTCCATACTGCTACATGCCGCCTCAGCATGCGCCTCAGGTTGGGGCTCCTGGGATGATTATGGGTAAGCCTATGATGGACCCATCTATGTATGCTCAACAGTCTCACCCTTACATGGGTCAGCCAATGTGGCAGCAGGCACCGGAGCAGCAGCAGTCACCATCAGATCATTAG
