# Supplementary material for: Genome-wide analysis of the NF-Y gene family in peach (Prunus persica L.)
Source: BMC Genomics. 2019 Jul 26;20:612. doi: 10.1186/s12864-019-5968-7 (PMC6660701; doi:10.1186/s12864-019-5968-7)
Supplement: Supplementary file 2 — Peach NF-Ys protein sequence. (DOCX 18 kb) [file 12864_2019_5968_MOESM2_ESM.docx]

>PpNF-YA1

MMPAKSKDEDPHIEHGAQTVLESAIYAQPWWRGVGNNSSSGESAPASSLVDHRDSMVMNGAMQSQANARLDGGANFNKELRTTGGSQSDQKNGCKNQQIKSVSSSVVPTMGEHLDPNSQMELVGHSIVLTSYPYSDPQYGGMLTPYGAQAMLPSHFYGIHHGRMPLPLEMEEEPVYVNAKQYHGILRRRQSRAKTELEKKLIKARKPYLHESRHLHALRRARGCGGRFLNTKKQDDNDENSSPEKGLSLDANDSAQSAKSSFQCFPNTSNGSLDSSNVQQEGSDPWFRAHKNHTLSSDNGNGNGRGPSSAYPSTFGDSKESVFLGQQRENMQLNGSSRGAIPSK

>PpNF-YA2

MGAPLQNCHNRKQSGIHFQDQDSSSTQSTGQSHSEVDSMKEGNPCGEGIVSAQSGYNERQGKPVGGHLKSLSSMASQGFVFPSQLDFSHPMGHIPFHYAEPYFGSLLAAACGPQATIHHPQVMGITPARVPLPLDLTEDEPIYVNAKQYRAILRRRQYRAKLEAQNKLVKIRKPYLHESRHVHALKRARGSGGRFLNMKKVQDSKPNTTNRVDVSGSAQLHLTRNMSESEVLEPDNYRDGASTTSCSEVTSTSNSDNIFPRQDFRFSGYPSHIGGTMQVPFVDVRGGGNQHHISLLR

>PpNF-YA3

MTSSVHDRSDNSESDDQQSHSSLPAIGISHPGIPTPNIQYAAPPQVGTGHAVAPAAYPYPDPYYRSIFAPYDTQPYPPQPYGGQPTVHLQLMGIQQAGVPLPSDAVEEPVFVNAKQYHGILRRRQSRAKAESENKALKSRKPYLHESRHQHALRRARGCGGRFLNAKKNDNQQDEMSSGDKSQPNINLNSDKSELASSDGTS

>PpNF-YA4

MQSKSETENRLHADHHTIPPNSVYSEPWWRGVGYNPISPAVTGRNASNSSSLECPNGGSESNDDRSLSNDEPNEDDDDDATKESQITSPRSAGNDGQERQNMPHVGSTVPTVRDDCLAQPPQLELVGHSIACASNPYQDPYYAGMMAAYGHQPLGYPPFLGMPHARMPLPLEMAQEPVYVNAKQYQGILRRRQARAKAELERKLIKVRKPYLHESRHQHAMRRARGTGGRFAKKTNGDKSNSTGQEKGTGSGPAHSSQSGSSSGSEPFPSDSAETWNSSNSQQEGRGSQVHEAYPGHNYANGSGCFQTHGGLQASMYPSYSGKRGEEGDCTGQQRGSISSNQASQRRLAIQ

>PpNF-YA5

MAMQTVYFKEHEGITNPMGQLSSALSGPWWSAFGSQPSYGESCGQSKPFTMEHPSSGDQLTATKLTGRGTEQGLDKGNTNQFTIFPGDGQKSQAAISLQSSPQKYRAHFELGFSQPMICAKYPYMDQCYGLFSTYGPQISGRIMLPLNLTADEGPIYVNAKQYHGIIRRRQSRAKAVMENRAAALRKPYMHESRHLHAMRRPRGCGGRFLNTKTINNGKNRTEGTQVGDGQLFRHSGSHSSELLQSESGTLNSSKETNGSSSNISGSEVTSVYSRRDLDRFSINHLRPSVHSLSDVMDSVRGMVIPTKWVAAGDNCCNLKV

>PpNF-YA6

MANVIEWLHTRRLHDSLIELSMPTQNLDKKFFSQSSVQSMPLYTTNCMSWNPIEREIPQSSSKDASFKVEFTPQVHHDAKHLGLQLLPDQGSSSTTQTISQSQDRCNSSESGEDEICGKGVEGQMKPVYLLNNLDLMSNPSQVGYGNLMGRVPYPYADPYFSGFLAAYGPQAMPQMMAPTRVPLPLDLAEDGPIYVNAKQYHGILRRRQSRAKLEAQNKLLKARKPYLHESRHLHALNRVRGSGGRFLSTKRLQQSDQNPSSSTHDVPDSINLHQKDTQDTESHHLGSSEFVTAVATHSDIASVSHTNDIFRQQDRRFSGIPSHMGGAMQFRGGLMRAGTQHCASVVR

>PpNF-YB1

MVDNNNNNIGASGAANHDEDGAMMKEQEQLLPIANVGRIMRQILPPNAKISKEAKETMQECVSEFISFVTSEASEKCRKERRKTVNGDDVSWALGALGFEDYTGPLRRFLHRYREQEGERISSSAANNNNNNNENNQDKDNNNPEDQQQRKLLNHPHIQRPNF

>PpNF-YB2

MAEAPTSPAGGSHESGGEQSPQGGGGGGSGVREQDRYLPIANISRIMKKALPQNGKIAKDAKDTVQECVSEFISFVTSEASDKCQKEKRKTINGDDLLWAMATLGFEDYIEPLRIYLARYRELEVTKRSLVLFNIFFYL

>PpNF-YB3

MADSDNDSGGNNDGSHAQSRELSAREQDRFLPIANVSRIMKKALPANAKISKDAKETVFVTGEASDKCQREKRKTINSDDLLWAMTTLRFEEYMEPLKVYLHKYRELEGEKTIMGGRDKDSAGGGGGGLPVEVAVTV

>PpNF-YB4

MERGGGGFHGYQKTSSGLKLSEINMRVAGMNQFSMQQGGKSTSSSTTNININNVVSNNNVVNHPTSSNGTTTTTDDNECVVREQDRFMPIANVIRIMRKILPPHAKISDDAKETIQECVSEYISFITGEANERCQREQRKTITAEDVLWAMSKLGFDDYIEPLTLYLHRYREMEGERGSGSSTTSVMRSDTSVKGAASSSSRAIDHHQYYAAAAAAAAFHHHHGHAFFGYLKPSDASTSTNNNINAAAAVAVAALPYCDQHQPHANDRA

>PpNF-YB5

MDHQKKRGYRPKWATTKFKCEINLIFLFFYTHSLWMDHGIIDVWLVPCNYEMKLKGCSSPPPPHQIIYISLTRTTTREQEEEEEEEKRITQDLHHPQRDSTTGFYRLQTMMKKGLPGNAKISKDAKETVQYFTTGEASGKCRREKRKTINGDDLLWAMTRLGFKEYVEPLKIYLHKFREMEGEKTAGTGMARQLLYADHHPHEYHDQRQREQLQHLHAPGMNMYGGMMMGHQRHHQGQGHHPYLYGSGSGPSSL

>PpNF-YB6

MADSDNDSGGAHTKSNSELSPREQDRLLPIANVSRIMKKALPANAKISKDAKETVQECVSEFISFITGEASDKCQREKRKTINGDDLLWAMTTLGFEEYVEPLKVYLQRFREMEGEKSSVAARDKDASGGGGNGGVGYESGGGSGGAGVYWQQQQQQQQHQHQHQHQGHVYGGSGFHQMGVSGVGLGKGGPGSNMGRPR

>PpNF-YB7

MRGPPSSRHAKSSSAQVNNSERSNSKVPINATPAATNSNAAAAAANAGDPAQLAPQQSVVREQDQYMPIANVIRIMRRILPPHAKISDDAKETIQECVSEYIAFITGEANERCRREQRKTVTAEDILWAMGKLGFDNYVEPLTLFLQKHRESENSDRSTLRAEFMKRDRAVDFGPAGPPIALMPPPPPPYGPGYPFGPQHGPGMFDPSMLGMFRDGSSSGSGGGAGSSSASGDQGQNSLEGFDPFAQFK

>PpNF-YB8

MRVAGMNQFSMQQGGKSTSSSTTNININNVVSNNNVVNHPTSSNGTTTTTDDNECVVREQDRFMPIANVIRIMRKILPPHAKISDDAKETIQECVSEYISFITGEANERCQREQRKTITAEDVLWAMSKLGFDDYIEPLTLYLHRYREMEGERGSGSSTTSVMRSDTSVKGAASSSSRAIDHHQYYAAAAAAAAFHHHHGHAFFGYLKPSDASTSTNNNINAAAAVAVAALPYCDQHQPHANDRA

>PpNF-YB9

MSARVSEPLNLHPTTNSSGTAQPEAKGEKKKKKVFQRTAQARAQDSTTNTQHKKEKGRKRKTSTHFPSLAFPFSISHNHSELNSMSGRRNQTSPVGSPLSGNVSDGSSKEQDKFLPIANVSRIMKKSLPANAKISKEAKETVQECVSEFISFITGEASDKCLREKRKTINGDDLLWAMTTLGFENYVGPLKGYLNKYRETEGEKNSMTRQEEDPSQQQQQHLNTSNTNMQHSSNNEQMNTVLNANNISMSTSKVDLFNGGFYFLEGQQQQQEVTQNYNLVSAGAYNLSRINESGDVNGNRDLATHHLHNGIGW

>PpNF-YB10

MAEAPGSPGGGSHESGGDQSPRSNVREQDRYLPIANISRIMKKALPANGKIAKDAKETVQECVSEFISFITSEASDKCQREKRKTINGDDLLWAMATLGFEDYIDPLKVYLTRYREMEGDTKGSGKGGDSSSKKDAQPSSNAQISRQGSFSQGGNYSNSQSQHMMVPMQGTE

>PpNF-YB11

MVDEQDGLLPIANVGRIMKQILPQRAKISKEAKQRMQECATEFLSFVTAEASDKCHKENRKTVNGDDICWALSALGFDNYAQATIRYLHKYREAERDKAAANANNNNQNKAADIILMSSGQDMNDIGDRAASIYMASQQGLQVGEQTQTPDLEFRLLGKGDGTKPSVDQEHN

>PpNF-YB12

MDNNIGNNSSEREGFKYNFTGSSISSDHHHHQEDGVIKEQDRLLPIANVGRIMKQILPPNAKISKEAKETMQECVSEFISFVTGEASDKCHKEKRKTVNGDDICWALATLGFDDYAEPLKRYLHRYRELEGEKAAHQGKANSSEEKNELSPPRTSTSSPLKFNVLERGNSSLSRRF

>PpNF-YC1

MDQQGHGHPTTMGVAGSAAQASYGVNPYQSGQMMGLSPTGSVGLMQSPTQPAGPPASSQLAQHQLAYQHIHQQQQQQLQQQLQSFWAEQYQEIEAVMDFKNHSLPLARIKKIMKADEDVRMISAEAPVIFARACEMFILELTLRSWNHTEENKRRTLQKNDIAAAITRTDIFDFLVDIVPREDLKDEVLASIPRGGNLPVGSSTDGVPYYYMPAQHAPQVGAPGMIVGKPVMDQTLYGQQTHPYIAQPTWPQQQQPHKDS

>PpNF-YC2

MDIPMGNLTSHENQVTELETFEAFETRNELARQFRPVLMDVNHSINITPSSNTSPEMHGFMSTGSFELRNYYSHPSSREEADQEAKQSSFTELQKEEIEIFWNQQLFEIQNTTVAKAHHELPLARVKRVMKSDGQVKKVSSETPVLFSKACELFIMELTLRSWLHTERSKRRTLQHCDTARAIMQDELLHFLVHAVPPLNSIARDYFEDDE

>PpNF-YC3

MNHSEQTLQQQQQEQHHQQQPVVGVVASGGQMTYAPPSYQTAPMVASGTPAVAVPSPTQPPAAFSNSSHQIAYQQAQHFHNQQQQQQQQQLQVFWANQMQDIEQASDFKNHSLPLARIKKIMKADEDVRMISAEAPVIFAKACEIFILELTLRSWIHTEENKRRTLQKNDIAAAISRTDVFDFLVDIIPRDELKEEGLGVTKATIPVVGSPADIPYYYVPPQHPVGAPGMIMGKPVDQAAIYAAQQPRPPMAFMPWSQPQPQQPQQQQQQQEAQHQQTDT

>PpNF-YC4

MRQPGRYSGFMMHGGISGRTGPHSLPLARIKKIMKKSGEDVKMISGEAPIVFSKACELFIEELTRRSWMTTLEGKRRTLHKDDVASAVVGTDIFDFLVSLVSDSCHSEDITPADKEALGGS

>PpNF-YC5

MDTNPNTTTTTTTPNPTQQQQQAQSSYPPTQSSVPAPPFHHLLQQQQQQLQMFWTYQRHDIEQVNDFKNHQLPLARIKKIMKADEDVRMISAEAPVLFAKACELFILELTIRSWLHAEENKRRTLQKNDIAAAITRTDIFDFLVDIVPRDEIKDEAVGLGGMVGATASGVPYYYPPMGQPAGGPGGMMIGRPAVDPAAVYGVQPPSQAWQSVWQTAADDGSYGSGGSSGQGNLDGQS

>PpNF-YC6

MDPQGHNQPQSMGMVGSGAQLTYGTNPYQHNQMVGSPNPGSVAGTVGAIQSTSQSAGAQLAQHQLAYQHIHHQQQQQLQQQLQSFWANQYQEIDKVTDFKNHSLPLARIKKIMKADEDVRMISAEAPVIFARACEMFILELTLRSWNHTEENKRRTLQKNDIAAAITRTDIFDFLVDIVPREDLKDEVLASIPRGTVSVGGPGDALPYCYMPPQHAPQVGAPGMIMGKPMMDPSMYAQQSHPYMGQPMWQQAPEQQQSPSDH
