# Supplementary material for: Genome-wide analysis of the NF-Y gene family in peach (Prunus persica L.)
Source: BMC Genomics. 2019 Jul 26;20:612. doi: 10.1186/s12864-019-5968-7 (PMC6660701; doi:10.1186/s12864-019-5968-7)
Supplement: Supplementary file 4 — Primer of Peach NF-Y family. (DOCX 19 kb) [file 12864_2019_5968_MOESM4_ESM.docx]

> AtNF-YA1

MQSKPGRENEEEVNNHHAVQQPMMYAEPWWKNNSFGVVPQARPSGIPSNSSSLDCPNGSESNDVHSASEDGALNGENDGTWKDSQAATSSRSVDNHGMEGNDPALSIRNMHDQPLVQPPELVGHYIACVPNPYQDPYYGGLMGAYGHQQLGFRPYLGMPRERTALPLDMAQEPVYVNAKQYEGILRRRKARAKAELERKVIRDRKPYLHESRHKHAMRRARASGGRFAKKSEVEAGEDAGGRDRERGSATNSSGSEQVETDSNETLNSSGAP

> AtNF-YA2

MAMQTVREGLFSAPQTSWWTAFGSQPLAPESLAGDSDSFAGVKVGSVGETGQRVDKQSNSATHLAFSLGDVKSPRLVPKPHGATFSMQSPCLELGFSQPPIYTKYPYGEQQYYGVVSAYGSQSRVMLPLNMETEDSTIYVNSKQYHGIIRRRQSRAKAAAVLDQKKLSSRCRKPYMHHSRHLHALRRPRGSGGRFLNTKSQNLENSGTNAKKGDGSMQIQSQPKPQQSNSQNSEVVHPENGTMNLSNGLNVSGSEVTSMNYFLSSPVHSLGGMVMPSKWIAAAAAMDNGCCNFKT

> AtNF-YA3

MMHQMLNKKDSATHSTLPYLNTSISWGVVPTDSVANRRGSAESLSLKVDSRPGHIQTTKQISFQDQDSSSTQSTGQSYTEVASSGDDNPSRQISFSAKSVVSLGSEITQRKGFASNPKQGSMTGFPNIHFAPAQANFSFHYADPHYGGLLAATYLPQAPTCNPQMVSMIPGRVPLPAELTETDPVFVNAKQYHAIMRRRQQRAKLEAQNKLIRARKPYLHESRHVHALKRPRGSGGRFLNTKKLLQESEQAAAREQEQDKLGQQVNRKTNMSRFEAHMLQNNKDRSSTTSGSDITSVSDGADIFGHTEFQFSGFPTPINRAMLVHGQSNDMHGGGDMHHFSVHI

> AtNF-YA4

NNESHAKKERPDSQTRPQVPSGRSSESIDTNSVYSEPMAHGLYPYPDPYYRSVFAQQAYLPHPYPGLMGMQQPGVPLQCDAVEEPVFVNAKQYHGILRRRQSRAKLEARNRAIKAKKPYMHESRHLHAIRRPRGCGGRFLNAKKENGDHKEEEEATSDENTSEASSSLRSEKLAMATSGPNGRS

> AtNF-YA5

MQVFQRKEDSSWGNSMPTTNSNIQGSESFSLTKDMIMSTTQLPAMKHSGLQLQNQDSTSSQSTEEESGGGEVASFGEYKRYGCSIVNNNLSGYIENLGKPIENYTKSITTSSMVSQDSVFPAPTSGQISWSLQCAETSHFNGFLAPEYASTPTALPHLEMMGLVSSRVPLPHHIQENEPIFVNAKQYHAILRRRKHRAKLEAQNKLIKCRKPYLHESRHLHALKRARGSGGRFLNTKKLQESSNSLCSSQMANGQNFSMSPHGGGSGIGSSSISPSSNSNCINMFQNPQFRFSGYPSTHHASALMSGT

> AtNF-YA6

MQEFHSSKDSLPCPATSWDNSVFTNSNVQGSSSLTDNNTLSLTMEMKQTGFQMQHYDSSSTQSTGGESYSEVASLSEPTNRYGHNIVVTHLSGPPLFNLFRLSYKENPENPIGSHSISKVSQDSVVLPIEAASWPLHGNVTPHFNGFLSFPYASQHTVQHPQIRGLVPSRMPLPHNIPENEPIFVNAKQYQAILRRRERRAKLEAQNKLIKVRKPYLHESRHLHALKRVRGSGGRFLNTKKHQESNSSLSPPFLIPPHVFKNSPGKFRQMDISRGGVVSSVSTTSCSDITGNNNDMFQQNPQFRFSGYPSNHHVSVLM

>AtNF-YA7

NIGSHEKQEQRDSHFQPPIPSARNYESIVTSLVYSDPGTTNSMAPGQYPYPDPYYRSIFAPPPQPYTGVHLQLMGVQQQGVPLPSDAVEEPVFVNAKQYHGILRRRQSRARLESQNKVIKSRKPYLHESRHLHAIRRPRGCGGRFLNAKKEDEHHEDSSHEEKSNLSAGKSAMAASS

>AtNF-YA8

MDKKVSFTSSVAHSTPPYLSTSISWGLPTKSNGVTESLSLKVVDARPERLINTKNISFQDQDSSSTLSSAQSSNDVTSSGDDNPSRQISFLAHSDVCKGFEETQRKRFAIKSGSSTAGIADIHSSPSKANFSFHYADPHFGGLMPAAYLPQATIWNPQMTRVPLPFDLIENEPVFVNAKQFHAIMRRRQQRAKLEAQNKLIKARKPYLHESRHVHALKRPRGSGGRFLNTKKLQESTDPKQDMPIQQQHATGNMSRFVLYQLQNSNDCDCSTTSRSDITSASDSVNLFGHSEFLISDCPSQTNPTMYVHGQSNDMHG

> AtNF-YA9

MHSKSDSGGNKVDSEVHGTVSSSINSLNPWHRAAAACNANSSVEAGDKSSKSIALALESNGSKSPSNRDNTVNKESQVTTSPQSAGDYSDKNQESLHHGITQPPPHPQLVGHTVGWASSNPYQDPYYAGVMGAYGHHPLGFVPYGGMPHSRMPLPPEMAQEPVFVNAKQYQAILRRRQARAKAELEKKLIKSRKPYLHESRHQHAMRRPRGTGGRFAKKTNTEASKRKAEEKSNGHVTQSPSSSNSDQGEAWNGDYRTPQGDEMQSSAYKRREEGECSGQQWNSLSSNHPSQARLAIK

> AtNF-YA10

MQTEELLSPPQTPWWNAFGSQPLTTESLSGEASDSFTGVKAVTTEAEQGVVDKQTSTTLFTFSPGGEKSSRDVPKPHVAFAMQSACFEFGFAQPMMYTKHPHVEQYYGVVSAYGSQRSSGRVMIPLKMETEEDGTIYVNSKQYHGIIRRRQSRAKAEKLSRCRKPYMHHSRHLHAMRRPRGSGGRFLNTKTADAAKQSKPSNSQSSEVFHPENETINSSREANESNLSDSAVTSMDYFLSSSAYSPGGMVMPIKWNAAAMDIGCCKLNI

> AtNF-YB1

MADTPSSPAGDGGESGGSVREQDRYLPIANISRIMKKALPPNGKIGKDAKDTVQECVSEFISFITSEASDKCQKEKRKTVNGDDLLWAMATLGFEDYLEPLKIYLARYREGDNKGSGKSGDGSNRDAGGGVSGEEMPGKLEISELFIVELIDNYFGFTLNFGY

>At NF-YB2

MGDSDRDSGGGQNGNNQNGQSSLSPREQDRFLPIANVSRIMKKALPANAKISKDAKETMQECVSEFISFVTGEASDKCQKEKRKTINGDDLLWAMTTLGFEDYVEPLKVYLQRFREIEGERTGLGRPQTGGEVGEHQRDAVGDGGGFYGGGGGMQYHQHHQFLHQQNHMYGATGGGSDSGGGAASGRTRT

>AtNF-YB3

MADSDNDSGGHKDGGNASTREQDRFLPIANVSRIMKKALPANAKISKDAKETVQECVSEFISFITGEASDKCQREKRKTINGDDLLWAMTTLGFEDYVEPLKVYLQKYREVEGEKTTTAGRQGDKEGGGGGGGAGSGSGGAPMYGGGMVTTMGHQFSHHFS

> AtNF-YB4

MTDEDRLLPIANVGRLMKQILPSNAKISKEAKQTVQECATEFISFVTCEASEKCHRENRKTVNGDDIWWALSTLGLDNYADAVGRHLHKYREAERERTEHNKGSNDSGNEKETNTRSDVQNQSTKFIRVVEKGSSSSAR

> AtNF-YB5

MAGNYHSFQNPIPRYQNYNFGSSSSNHQHEHDGLVVVVEDQQQEESMMVKEQDRLLPIANVGRIMKNILPANAKVSKEAKETMQECVSEFISFVTGEASDKCHKEKRKTVNGDDICWAMANLGFDDYAAQLKKYLHRYRVLE

> AtNF-YB6

MERGGFHGYRKLSVNNTTPSPPGLAANFLMAEGSMRPPEFNQPNKTSNGGEEECTVREQDRFMPIANVIRIMRRILPAHAKISDDSKETIQECVSEYISFITGEANERCQREQRKTITAEDVLWAMSKLGFDDYIEPLTLYLHRYRELEGERGVSCSAGSVSMTNGLVVKRPNGTMTEYGAYGPVPGIHMAQYHYRHQNGFVFSGNEPNSKMSGSSSGASGARVEVFPTQQHKY

> AtNF-YB7

MTEESPEEDHGSPGVAETNPGSPSSKTNNNNNNNKEQDRFLPIANVGRIMKKVLPGNGKISKDAKETVQECVSEFISFVTGEASDKCQREKRKTINGDDIIWAITTLGFEDYVAPLKVYLCKYRDTEGEKVNSPKQQQQRQQQQQIQQQNHHNYQFQEQDQNNNNMSCTSYISHHHPSPFLPVDHQPFPNIAFSPKSLQKQFPQQHDNNIDSIHW

> AtNF-YB8

MAESQAKSPGGCGSHESGGDQSPRSLHVREQDRFLPIANISRIMKRGLPANGKIAKDAKEIVQECVSEFISFVTSEASDKCQREKRKTINGDDLLWAMATLGFEDYMEPLKVYLMRYREGDTKGSAKGGDPNAKKDGQSSQNGQFSQLAHQGPYGNSQAQQHMMVPMPGTD

> AtNF-YB9

MTSSVVVAGAGDKNNGIVVQQQPPCVAREQDQYMPIANVIRIMRKTLPSHAKISDDAKETIQECVSEYISFVTGEANERCQREQRKTITAEDILWAMSKLGFDNYVDPLTVFINRYREIETDRGSALRGEPPSLRQTYGGNGIGFHGPSHGLPPPGPYGYGMLDQSMVMGGGRYYQNGSSGQDESSVGGGSSSSINGMPAFDHY

> AtNF-YB10

MAESQTGGGGGGSHESGGDQSPRSLNVREQDRFLPIANISRIMKRGLPLNGKIAKDAKETMQECVSEFISFVTSEASDKCQREKRKTINGDDLLWAMATLGFEDYIDPLKVYLMRYREMEGDTKGSGKGGESSAKRDGQPSQVSQFSQVPQQGSFSQGPYGNSQGSNMMVQMPGTE

> AtNF-YB11

MESEKVVVDELPLAIVRRVVKKKLSECSPDYDVSIHKEALLAFSESARIFIHYLSATANDFCKDARRQTMKADDVFKALEEMDFSEFLEPLKSSLEDFKKKNAGKKAGAAAASYPAGGAALKSSSGTASKPKETKKRKQEEPSTQKGARKSKIDEETKRNDEETENDNTEEENGNDEEDENGNDEEDENDDENTEENGNDEENDDENTEENGNDEENEKEDEENSMEENGNESEESGNEDHSMEENGSGVGEDNENEDGSVSGSGEEVESDEEDE

> AtNF-YB12

MDPMDIVGKSKEDASLPKATMTKIIKEMLPADVRVARDAQDLLIECCVEFINLISSESNEVCNKEDKRTIAPEHVLKALQVLGFGEYVEEVYAAYEQHKYETMQDSQRSVKMNSGAEMTEEEAAAEQQRMFAEARARMNGGVTVPQPEQLEEPQQQQQTSLQS

> AtNF-YB13

MDPMDIVGKSKEDASLPKATMTKIIKEMLPPDVRVARDAQDLLIECCVEFINLVSSESNDVCNKEDKRTIAPEHVLKALQVLGFGEYIEEVYAAYEQHKYETMQDTQRSVKWNPGAQMTEEEAAAEQQRMFAEARARMNGGVSVPQPEHPETDQRSPQS

> AtNF-YC1

MDTNNQQPPPSAAGIPPPPPGTTISAAGGGASYHHLLQQQQQQLQLFWTYQRQEIEQVNDFKNHQLPLARIKKIMKADEDVRMISAEAPILFAKACELFILELTIRSWLHAEENKRRTLQKNDIAAAITRTDIFDFLVDIVPRDEIKDEAAVLGGGMVVAPTASGVPYYYPPMGQPAGPGGMMIGRPAMDPNGVYVQPPSQAWQSVWQTSTGTGDDVSYGSGGSSGQGNLDGQG

> AtNF-YC2

MEQSEEGQQQQQQGVMDYVPPHAYQSGPVNAASHMAFQQAHHFHHHHQQQQQQQLQMFWANQMQEIEHTTDFKNHTLPLARIKKIMKADEDVRMISAEAPVIFAKACEMFILELTLRAWIHTEENKRRTLQKNDIAAAISRTDVFDFLVDIIPRDELKEEGLGVTKGTIPSVVGSPPYYYLQQQGMMQHWPQEQHPDES

> AtNF-YC3

MDQQGQSSAMNYGSNPYQTNAMTTTPTGSDHPAYHQIHQQQQQQLTQQLQSFWETQFKEIEKTTDFKNHSLPLARIKKIMKADEDVRMISAEAPVVFARACEMFILELTLRSWNHTEENKRRTLQKNDIAAAVTRTDIFDFLVDIVPREDLRDEVLGGVGAEAATAAGYPYGYLPPGTAPIGNPGMVMGNPGAYPPNPYMGQPMWQQPGPEQQDPDN

> AtNF-YC4

MDNNNNNNNQQPPPTSVYPPGSAVTTVIPPPPSGSASIVTGGGATYHHLLQQQQQQLQMFWTYQRQEIEQVNDFKNHQLPLARIKKIMKADEDVRMISAEAPILFAKACELFILELTIRSWLHAEENKRRTLQKNDIAAAITRTDIFDFLVDIVPREEIKEEEDAASALGGGGMVAPAASGVPYYYPPMGQPAVPGGMMIGRPAMDPSGVYAQPPSQAWQSVWQNSAGGGDDVSYGSGGSSGHGNLDSQG

> AtNF-YC5

MENNNNNHQQPPKDNEQLKSFWSKGMEGDLNVKNHEFPISRIKRIMKFDPDVSMIAAEAPNLLSKACEMFVMDLTMRSWLHAQESNRLTIRKSDVDAVVSQTVIFDFLRDDVPKDEGEPVVAAADPVDDVADHVAVPDLNNEELPPGTVIGTPVCYGLGIHAPHPQMPGAWTEEDATGANGGNGGN

> AtNF-YC6

MAENNNNNGDNMNNDNHQQPPSYSQLPPMASSNPQLRNYWIEQMETVSDFKNRQLPLARIKKIMKADPDVHMVSAEAPIIFAKACEMFIVDLTMRSWLKAEENKRHTLQKSDISNAVASSFTYDFLLDVVPKDESIATADPGFVAMPHPDGGGVPQYYYPPGVVMGTPMVGSGMYAPSQAWPAAAGDGEDDAEDNGGNGGGN

> AtNF-YC7

MEENNGNNNHYLPQPSSSQLPPPPLYYQSMPLPSYSLPLPYSPQMRNYWIAQMGNATDVKHHAFPLTRIKKIMKSNPEVNMVTAEAPVLISKACEMLILDLTMRSWLHTVEGGRQTLKRSDTLTRSDISAATTRSFKFTFLGDVVPRDPSVVTDDPVLHPDGEVLPPGTVIGYPVFDCNGVYASPPQMQEWPAVPGDGEEAAGEIGGSSGGN

> AtNF-YC8

MENNNGNNQLPPKGNEQLKSFWSKEMEGNLDFKNHDLPITRIKKIMKYDPDVTMIASEAPILLSKACEMFIMDLTMRSWLHAQESKRVTLQKSNVDAAVAQTVIFDFLLDDDIEVKRESVAAAADPVAMPPIDDGELPPGMVIGTPVCCSLGIHQPQPQMQAWPGAWTSVSGEEEEARGKKGGDDGN

> AtNF-YC9

MDQQDHGQSGAMNYGTNPYQTNPMSTTAATVAGGAAQPGQLAFHQIHQQQQQQQLAQQLQAFWENQFKEIEKTTDFKNHSLPLARIKKIMKADEDVRMISAEAPVVFARACEMFILELTLRSWNHTEENKRRTLQKNDIAAAVTRTDIFDFLVDIVPREDLRDEVLGSIPRGTVPEAAAAGYPYGYLPAGTAPIGNPGMVMGNPGGAYPPNPYMGQPMWQQQAPDQPDQEN

> AtNF-YC10

MVSSKKPKEKKARSDVVVNKASGRSKRSSGSRTKKTSNKVNIVKKKPEIYEISESSSSDSVEEAIRGDEAKKSNGVVSKRGNGKSVGIPTKTSKNREEDDGGAEDAKIKFPMNRIRRIMRSDNSAPQIMQDAVFLVNKATEMFIERFSEEAYDSSVKDKKKFIHYKHLSSVVSNDQRYEFLADSVPEKLKAEAALEEWERGMTDAG

> AtNF-YC11

MRKKLDTRFPAARIKKIMQADEDVGKIALAVPVLVSKSLELFLQDLCDRTYEITLERGAKTVSSLHLKHCVERYNVFDFLREVVSKVPDYGHSQGQGHGDVTMDDRSISKRRKPISDEVNDSDEEYKKSKTQEIGSAKTSGRGGRGRGRGRGRGGRAAKAAEREGLNREMEVEAANSGQPPPEDNVKMHASESSPQEDEKKGIDGTAASNEDTKQHLQSPKEGIDFDLNAESLDLNETKLAPATGTTTTTTAATDSEEYSGWPMMDISKMDPAQLASLGKRIDEDEEDYDEEG

> AtNF-YC12

MRRPKSSHVRMEPVAPRSHNTMPMLDQFRSNHPETSKIEGVSSLDTALKVFWNNQREQLGNFAGQTHLPLSRVRKILKSDPEVKKISCDVPALFSKACEYFILEVTLRAWMHTQSCTRETIRRCDIFQAVKNSGTYDFLIDRVPFGPHCVTHQGVQPPAEMILPDMNVPIDMDQIEEENMMEERSVGFDLNCDLQ

> AtNF-YC13

MEEEEGSIRPEFPIGRVKKIMKLDKDINKINSEALHVITYSTELFLHFLAEKSAVVTAEKKRKTVNLDHLRIAVKRHQPTSDFLLDSLPLPAQPVKHTKSVSDKKIPAPPIGTRRIDDFFSKGKAKTDSA
